# Predictive gene expression signatures for Alzheimer’s disease using post-mortem brain tissue

**DOI:** 10.3389/fnagi.2025.1591946

**Published:** 2025-10-02

**Authors:** Ashley H. Duche, Oliver Tan, Andrius Baskys, Rachita K. Sumbria, Moom R. Roosan

**Affiliations:** ^1^Pharmacy Practice Department, School of Pharmacy, Chapman University, Irvine, CA, United States; ^2^College of Health Sciences and Specialty Clinics, Clinical Genomics and Pharmacogenetics Service, Western University of Health Sciences, Pomona, CA, United States; ^3^Department of Neurology, University of California, Irvine, Irvine, CA, United States

**Keywords:** gene expression signatures, predictive gene signatures, differential gene expression, transcriptomics, Alzheimer’s disease, differentially expressed genes

## Abstract

**Background:**

Alzheimer’s Disease (AD) is a progressive neurodegenerative disorder characterized by amyloid-beta (Aβ) plaques and tau protein aggregates in the brain. These pathological features manifest in specific regions, but mechanisms rendering some areas more susceptible to early AD-related changes remain poorly understood. To address this, we developed predictive gene expression signatures to explore molecular mechanisms underlying regional vulnerability to AD pathology.

**Methods:**

Post-mortem brain tissues from participants of the Religious Orders Study and Memory and Aging Project (ROSMAP), Mayo Clinic, and Mount Sinai Brain Bank (MSBB) were used to derive gene expression signatures from six brain regions affected at varying stages of AD progression. Differential gene expression analysis identified genes with altered expression patterns which were used to develop predictive gene signatures using Adaptive Signature Selection and InteGratioN (ASSIGN) to predict pathway activity. Predictions of AD activity were validated against known AD status across clinical markers of AD pathology, including Aβ plaque deposition, tau aggregates, cognitive assessments, and clinical diagnoses. Dysregulation of key biological pathways was then analyzed using g: Profiler and ClueGO. Additionally, genetic and sociodemographic factors impacting AD prediction were assessed, and potential drug repurposing candidates identified using Connectivity Map (CMAP).

**Results:**

Predictive gene expression signatures from six AD-affected brain regions distinguished AD activity in control and AD post-mortem brain tissue, corresponding to clinical markers of disease severity. The signatures revealed common underlying mechanisms of regional vulnerability, including upregulation of extracellular matrix (ECM)-related processes and downregulation of hormonal signaling pathways. Notably, *S100A4* was consistently upregulated across all regions, while *CRH* expression was downregulated except in the cerebellum. Additionally, findings underscored the influence of APOE genotype (e3/e4) and sex on disease progression. Drug repurposing analysis identified FGFR inhibitors, specifically orantinib and bromodomain inhibitors, as promising therapeutic candidates.

**Conclusion:**

Molecular signatures underlying regional vulnerability to AD provide a framework for understanding genetic and systemic factors in disease progression. Findings highlight specific molecular pathways, including ECM-related processes and hormonal regulation, as key drivers of susceptibility. Finally, identified drug repurposing candidates present promising therapeutic avenues for further investigation.

## Introduction

Alzheimer’s disease (AD) is a complex neurodegenerative disorder that affects various brain regions at varying stages of progression (Monteiro et al., 2023). The hippocampus is among the earliest regions impacted, while the frontal cortex is affected in later stages (Monteiro et al., 2023). This regional variability is a hallmark of AD and contributes to the heterogeneity in cognitive decline and clinical outcomes. Investigating gene expression changes across brain regions throughout disease progression can provide crucial insights into the molecular mechanisms underlying AD.

To study these AD-related changes across brain regions, this study aims to capture region-specific gene expression signatures from post-mortem brain (PMB) tissue using differential gene expression (DGE) analysis. Furthermore, the predictive ability of these signatures to detect AD-related changes will be validated by comparing them with established clinical markers of AD, such as cognitive assessments, amyloid-beta (Aβ) plaque deposition, and tau protein aggregates, measured through the Consortium to Establish a Registry for Alzheimer’s Disease (CERAD) scoring, Thal phases, Braak staging systems, and clinical diagnoses. This combined approach enhances our understanding of the molecular underpinnings of AD and could provide more reliable biomarker for diagnosis.

Previous studies successfully identified gene expression signatures related to inflammation, immune response, synaptic function, and neural signaling (Kodam et al., 2023). However, the ability of these signatures to predict AD status and progression across independent datasets and validate those predictions with known AD markers remains underexplored. This highlights a critical gap in our ability to predict the extent of disease progression from gene expression data and validate these predictions using known clinical and pathological markers.

This study addresses this gap by developing region-specific gene expression signatures that are not only predictive of AD but also validated against established clinical markers, ensuring that the predictions correspond to clinical AD diagnosis. Although the region-specific signatures are developed using PMB tissue, our goal is to capture the most severe gene expression changes which will allow us to use the signature to predict less severe cases effectively.

Moreover, imaging and CSF analysis for Alzheimer’s diagnosis such as PET scans and CSF tests remain expensive, invasive, and inaccessible for many patients. Gene expression signatures from PMB tissue could pave the way for developing more accessible biomarkers, enabling earlier and potentially real-time AD predictions from blood or other bodily fluids. Thus, by identifying molecular signatures that mirror disease progression, we aim to create predictive tools that complement current diagnostic approaches.

To perform this analysis, three distinct studies were utilized including the Religious Orders Study and Memory and Aging Project (ROSMAP), Mayo Clinic, and Mount Sinai Brain Bank (MSBB). These studies provide comprehensive gene expression data from various brain regions affected by AD allowing us to investigate how AD-related changes in gene expression vary by region. This offers an opportunity to predict disease status based on these molecular signatures. By comparing gene expression signatures between early-affected regions (e.g., hippocampus), mid-affected regions (e.g., association cortices) and later-affected regions (e.g., frontal lobe), we aim to capture the progression of AD across the brain.

This approach will also quantify the magnitude of AD’s impact across different brain regions. For example, it is hypothesized that certain brain regions, like the hippocampus, will exhibit more pronounced gene expression changes early in the disease, while others, such as the frontal cortex, will show more significant changes in later stages. These region-specific gene signatures could provide insights into the differential vulnerability of brain areas to AD pathology. Although the PMB tissue is not ideal in assessing real-time gene expression changes, these available datasets can be used to identify the extent of changes from terminal AD in each brain region. Furthermore, by validating the predicted AD status with clinical markers, this study aims to ensure that these gene expression signatures are not only reflective of molecular changes but also predictive of clinical diagnosis.

**Figure 1:**
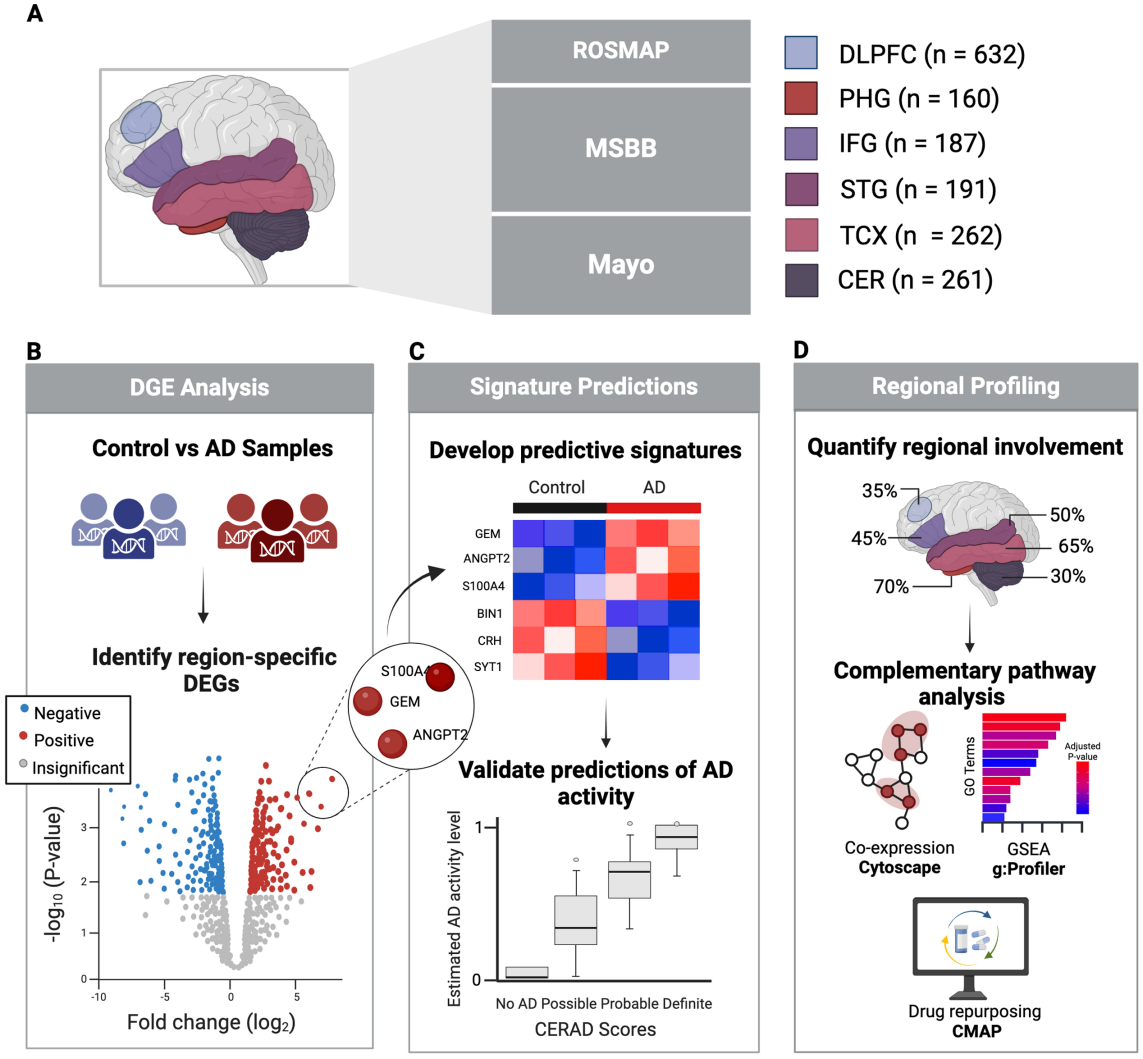
Study overview. **(A)** Healthy and AD PMB tissue samples collected from six regions sourced from three major studies. **(B)** Identification of significant DEGs in each region comparing AD vs. control using clinical and pathological markers. **(C)** Development of predictive signatures from DEGs to predict AD activity, validated against clinical and neuropathological AD status. **(D)** Regional profiling and pathway analysis to explore regional variability in AD involvement. Created in BioRender (Duche, 2025).

Thus, this study seeks to develop predictive gene expression signatures from PMB tissues that are region-specific and validated by clinical markers of AD (Figure 1). Through comprehensive analysis of multiple datasets, we aim to provide robust tools for predicting AD status, quantifying the magnitude of disease impact across brain regions, and ultimately improving early diagnosis and therapeutic intervention.

## Materials and methods

### Data and preprocessing

Bulk RNA-seq gene count data of PMB tissue samples were collected from The RNAseq Harmonization study, aimed to uniformly process gene expression data generated by the Accelerating Medicines Partnership for Alzheimer’s Disease (AMP-AD) cohort studies including ROSMAP (Bennett et al., 2018), MSBB (Wang et al., 2018), and the Mayo Clinic RNA Sequencing Study (Allen et al., 2016). ROSMAP provided 632 samples from the dorsolateral prefrontal cortex (DLPFC), MSBB contributed 160 samples from the parahippocampal gyrus (PHG, BM36), 187 from the inferior frontal gyrus (IFG, BM44), and 191 from the superior temporal gyrus (STG, BM22). Mayo supplied 262 samples from the temporal cortex (TCX) and 261 from the cerebellum (CER). Additionally, bulk RNA-seq FPKM expression data was obtained from the Aging, Dementia, and Traumatic Brain Injury Study (Accession GSE104687) through Gene Expression Omnibus (GEO). This included 98 TCX and 94 hippocampus samples, used for external validation of region-specific signatures. Respective meta data was acquired for each dataset.

To ensure consistency across the ROSMAP, MSBB, Mayo, and GSE104687 datasets for downstream analysis, CERAD scores (neuritic plaque density) and Braak stages (neurofibrillary tangle severity) were standardized (Mock et al., 2020; Supplementary Table 1). Datasets were further refined by excluding genes not common across all datasets or those with fewer than 10 counts per sample, across all samples. Next, samples were excluded based on RNA Integrity Number (RIN) thresholds, missing metadata, or outlier identification via Principal Component Analysis (PCA). Batch effects, such as sequencing batches, were then corrected using *sva* package (version 3.50.0; Zhang et al., 2020; Supplementary Table 2). All analysis steps were performed using R version 4.3.3 (Team, 2024).

### Differential gene expression analysis

Control and AD training groups were determined for each region using a combination of CERAD scores, Braak stages and study-specific diagnostic criteria (Supplementary Table 3). Patient cohort demographics were also assessed between control and AD groups to evaluate potential confounding differences (Supplementary Table 4). Control and AD samples were compared to identify differentially expressed genes (DEGs) using DESeq2 (version 1.42.1; Love et al., 2014). Statistically significant DEGs were identified using the Benjamini–Hochberg procedure to control for false positives, with a p adjusted (*p*-adj) ≤ 0.05, and region-specific log2 fold change (LFC) thresholds to optimize the downstream predictive performance (Supplementary Table 5).

### Development of gene expression signature predictions

Next, the DESeq2-normalized data was log2-transformed to stabilize variance and improve interpretability. The Adaptive Signature Selection and Integration (ASSIGN; version 1.38.0) toolkit was then used to develop predictive gene expression signatures for each region using their DEGs (Shen et al., 2015). This required region-specific control and AD training groups using stricter criteria than in DGE analysis (Supplementary Table 6). These training sets were used to train the ASSIGN model to improve prediction accuracy. Finally, the DEGs from each region were used to estimate AD activity on a scale from 0 (no activity) to 1 (highest activity) on the remaining samples.

### Validation of signature predictions across neuropathological and clinical measures

To validate each signature’s ability to predict AD status, predicted AD activity was compared to known AD status across meta categories including Braak stages, Aβ plaque measures (e.g., CERAD scores, Thal phases), and dataset-specific diagnoses (e.g., Cogdx, CDR, Diagnosis). Additionally, for the DLPFC (ROSMAP), Mini-Mental State Examination (MMSE) scores were used as an additional measure to assess cognitive impairment on a scale of 0–30 (0–12: Severe; 13–19: Moderate; 20–24: Mild; 25–30: Normal; Truong et al., 2024). For PHG, IFG, and STG, plaque mean was applied as a continuous measure of the average Aβ plaque density on a scale of 0–28 (higher scores represent higher plaque burden). Independent Student’s t-tests were used to determine significant differences in mean predicted AD activity levels between control and most severe AD groups, across all meta categories while Pearson’s correlation was applied for plaque mean with significance determined using *p* < 0.05 for all validation.

### Identifying key predictors of AD status using generalized linear modeling

A binomial generalized linear model (GLM) was constructed for each region using *stats* package (version 4.3.3; Team, 2024). This was used to quantify the influence of tissue-specific effects and external variables on predicting AD status. Models included region-specific signature predictions along with known associated factors such as age of death, sex, APOE genotype, and race (if available). Odds ratios (ORs) were calculated to assess the strength and direction of each predictor’s effect on AD status while the best-fit model was selected based on the lowest Akaike Information Criterion (AIC) using the stats package (version 4.3.3).

The goodness-of-fit of the predictive model was assessed using McFadden pseudo-R^2^, which represents the proportion of variability explained by all factors. This was computed using the *pscl* package (version 1.5.9) where pseudo-R^2^ values between 0.2–0.4 were moderate, 0.4–0.5 was strong, and > 0.5 was very strong model fit (Zeileis et al., 2008). This analysis helped identify which brain regions and clinical factors most effectively predict AD status.

To further evaluate the model’s predictive accuracy and reduce the risk of overfitting, Leave-One-Out Cross-Validation (LOOCV) was performed using the *caret* package (version 6.0.94; Kuhn, 2008). LOOCV was set up for binary classification (No AD or AD) with class probabilities, and the area under the Receiver Operating Characteristic (ROC) curve (AUC) was used as the primary performance metric. Models with AUC > 0.7 were considered to have acceptable predictive performance. This step ensured the reliability of the predictive signatures and factors included in the best-fit models.

### External validation of signatures

TCX and hippocampus FPKM datasets from the Aging, Dementia, and TBI study (GSE104687) was filtered to retain genes common across all signature development datasets, excluding samples with RIN < 5 or reported traumatic brain injury (TBI), and including only those diagnosed with “Alzheimer’s Disease Type” or “No Dementia,” followed by log2-normalization (Supplementary Table 2). Control and AD training sets for each region were defined using CERAD scores, Braak stages, and DSM-IV clinical diagnosis (Supplementary Table 5). Signature performance in predicting AD status from ASSIGN was validated using independent Student’s t-tests to compare control and severe AD groups.

### Gene and connectivity analysis

Genes included in the region-specific signatures were compared across regions to identify overlapping genes revealing common molecular patterns and region-specific differences.

Next, gene co-expression networks were generated for each brain region using GeneMANIA (Montojo et al., 2010) in Cytoscape (Shannon et al., 2003). Genes were ranked by degree, betweenness, and closeness centrality via CytoHubba (Chin et al., 2014). The top 10 genes for each measure were identified, and those overlapping across all three were classified as hub genes, representing key coordinators of molecular interactions.

Connectivity Map (CMAP) was then used to compare region-specific signature genes with expression profiles from various perturbations, including drugs, gene overexpression, knockdowns, and CMAP classes (Subramanian et al., 2017). Upregulated and downregulated gene lists from each region were queried to compute connectivity scores (CS; −100 to 100), with values above 90 indicating similarity and below −90 indicating dissimilarity. This allowed the identification of potential drug repurposing candidates.

### Pathway and network analysis

Gene Set Enrichment Analysis (GSEA) was conducted separately for each region using the full signature gene list, with analyses performed independently on upregulated and downregulated genes to identify enriched functional annotations and pathways. Analysis was performed using g: Profiler, which integrates data from Gene Ontology (GO), KEGG, Reactome, WikiPathways, CORUM, and Human Phenotype Ontology (Raudvere et al., 2019). Statistically significant pathways (*p* < 0.05) were identified and compared to determine shared and unique molecular mechanisms upregulated or downregulated across regions.

Next, using the gene co-expression networks in Cytoscape, MCODE (version 2.0.3) was used to identify tightly interconnected groups of genes, referred to as functional modules, using default parameters (Bader and Hogue, 2003). Functional modules identified with higher MCODE scores indicate more densely clustered and interconnected genes that may participate in critical biological processes or functions. A higher number of functional clusters indicates greater dysregulation in a region, providing a quantitative measure of the extent and coordination of changes occurring across regions.

ClueGO (version 2.5.10) in Cytoscape was then used for functional enrichment analysis of genes from functional module clusters from each region. ClueGO also integrates GO, KEGG, Reactome, WikiPathways, and CORUM data sources (Bindea et al., 2009). Results were then visualized as networks with nodes representing enriched annotations, and processes connected by edges and grouped by color based on gene overlap revealing functionally related processes. A *p* ≤ 0.05 was used for all regions, except PHG, which used *p* ≤ 0.01 to improve interpretability of dysregulated processes. A two-sided hypergeometric test with Bonferroni step-down correction was applied.

## Results

### Differential gene expression across AD-affected brain regions

**Figure 2:**
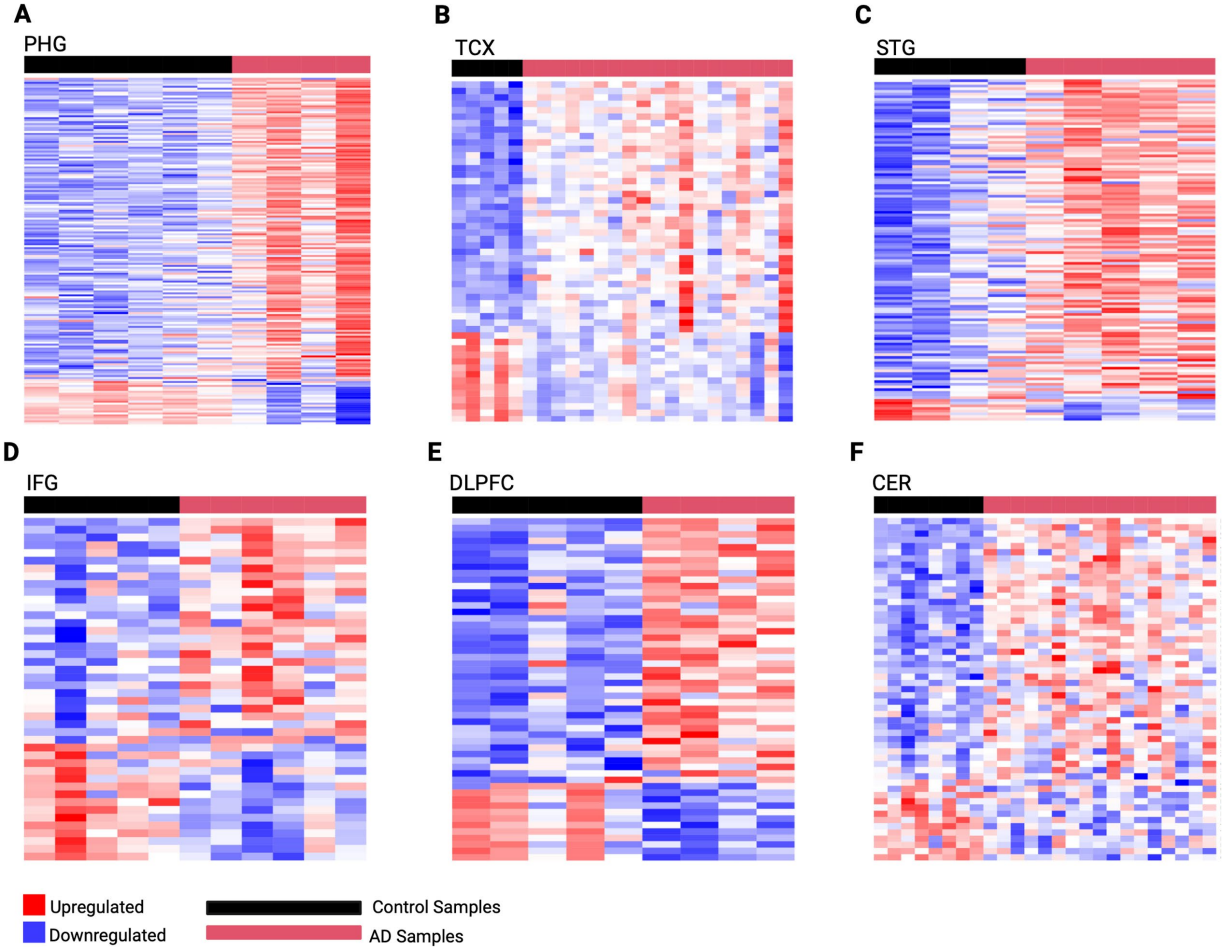
Predictive gene expression signatures developed from region-specific DEGs. **(A)** PHG 176 gene signature **(B)** TCX 53 gene signature **(C)** STG 127 gene signature **(D)** IFG 44 gene signature **(E)** DLPFC 53 gene signature **(F)** CER 55 gene signature. The horizontal red bar represents AD samples, while the horizontal black bar represents control samples for each region. Genes are rows with upregulated expression shown in red, and downregulated expression in blue, with deeper colors indicating greater levels of upregulation or downregulation, respectively.

To better understand the mechanisms driving AD pathology, this analysis captured gene expression changes across brain regions affected at different stages of the disease using PMB tissue (Supplementary Tables 7A–F). This revealed the highest dysregulation occurring in early-affected regions such as the PHG which we identified 176 DEGs (154 upregulated and 22 downregulated). This was followed by mid-affected regions including STG with 127 DEGs (119 upregulated, 8 downregulated), and TCX with 53 DEGs (39 upregulated, 14 downregulated). While mid-to-late affected regions showed less dysregulation, including the IFG with 44 DEGs (29 upregulated, 15 downregulated), DLPFC with 53 DEGs (40 upregulated, 13 downregulated), and CER with 55 DEGS (42 upregulated, 13 downregulated). Using these DEGs, predictive gene expression signatures were developed for each region representative of AD activity (Figure 2).

### Development of gene expression signatures predictive of AD status

These region-specific gene expression signatures were then used to predict the extent of AD, i.e., AD activity, in their respective PMB tissue. Predictions of AD were validated against the known AD status of established neuropathological, clinical, and cognitive assessments (Table 1; Supplementary Figure 1). Specifically, we assessed the biological relevance of our gene expression signatures with postmortem neuropathological measures, including Braak staging—which captures the distribution and severity of neurofibrillary tangles composed of hyperphosphorylated tau—and CERAD scores, which quantify the density of neuritic amyloid plaques. Both are widely recognized as robust markers of AD pathology. The observed alignment between our predictive signatures and these independent, pathology-based indicators underscores the disease relevance of the captured transcriptional changes. Notably, mean predicted AD activity levels in control groups were significantly different than in severe AD groups (Table 1). Additionally, a significant positive association was observed between predicted AD activity and plaque burden in PHG (Pearson r = 0.560, *p*-adj < 0.005, 95% CI [0.322, 0.731]), IFG (Pearson r = 0.566, *p*-adj < 0.005, 95% CI [0.366, 0.715]), and STG (Pearson r = 0.387, *p*-adj < 0.005, 95% CI [0.0495, 0.644]). These measures reflect the expected Aβ and tau burden at different stages of the disease, along with cognitive scores and clinical diagnoses, offering a comprehensive view of how the predicted AD activity aligns with the expected progression of real-world AD.

**Table 1:** Validation of region-specific signatures by comparing predicted AD activity with known AD status using neuropathological, cognitive, and clinical data.

Study	Region	Criteria	Comparison groups (control vs. AD)	Sample size (*N*)	Mean	Standard deviation (SD)	Adjusted *p*-value (*p*-adj)	95% Confidence interval (CI)
ROSMAP	DLPFC	Braak	0	5	0.0223	0.050	<0.005	[−1.03, −0.389]
			VI	5	0.733	0.261		
		CERAD	No AD	47	0.203	0.271	<0.005	[−0.478, −0.236]
			Definite AD	58	0.560	0.354		
		Cogdx	NCI (1)	64	0.204	0.259	<0.005	[−0.422, −0.235]
			AD (NINCDSADRDA) (4)	95	0.532	0.338		
		MMSE	Normal (25−30)	65	0.197	0.259	<0.005	[-0.520, −0.267]
			Severe CI (0−12)	44	0.590	0.362		
MSBB	PHG	Braak	≤ I	8	0.0632	0.131	<0.005	[−0.842, −0.488]
			VI	13	0.728	0.255		
		CERAD	No AD	22	0.161	0.219	<0.005	[−0.708, −0.364]
			Definite AD	16	0.697	0.279		
		CDR	NCD/Questionable (≤0.5)	27	0.164	0.219	<0.005	[−0.799, −0.363]
			Severe to terminal (≥3)	10	0.744	0.290		
	IFG	Braak	≤I	12	0.0994	0.233	<0.005	[−0.601, −0.250]
			≥V	36	0.525	0.313		
		CERAD	No AD	29	0.0752	0.186	<0.005	[−0.626, −0.360]
			Definite AD	7	0.568	0.335		
		CDR	NCD/Questionable (≤0.5)	36	0.0753	0.185	<0.005	[−0.857, −0.372]
			Severe to terminal (5)	8	0.690	0.287		
	STG	Braak	≤I	12	0.107	0.178	<0.005	[−0.797, −0.433]
			VI	23	0.727	0.337		
		CERAD	No AD	17	0.141	0.202	<0.005	[−0.748, −0.409]
			Definite AD	27	0.720	0.355		
		CDR	NCD (0)	10	0.246	0.274	<0.005	[−0.826, 0.325]
			Severe to terminal (≥4)	8	0.821	0.227		
Mayo	TCX	Braak	I	9	1.13×10^−5^	2.18×10^−5^	<0.005	[−0.881, 0.705]
			VI	20	0.793	0.189		
		Diagnosis	"Control"	34	0.0559	0.156	<0.005	[−0.814, −0.617]
			"Alzheimer’s Disease"	34	0.771	0.240		
		Thal	No Aβ plaques (0)	21	0.0526	0.164	<0.005	[−0.836, −0.618]
			Pons and cerebellum (5)	32	0.779	0.231		
	CER	Braak	≤I	12	1.15×10^−4^	2.79×10^−4^	<0.005	[−0.782, −0.490]
			VI	22	0.636	0.329		
		Diagnosis	"Control"	37	0.0844	0.197	<0.005	[−0.621, −0.352]
			"Alzheimer’s Disease"	38	0.571	0.362		
		Thal	No Aβ plaques (0)	24	0.112	0.228	<0.005	[−0.608, −0.299]
			Pons and cerebellum (5)	36	0.565	0.370		

Independent Student’s *t*-tests showed significant differences in mean predicted AD activity between control and severe AD groups, confirming their ability to distinguish AD in PMB brain tissue. AD, Alzheimer’s Disease; ROSMAP, The Religious Orders Study and Memory and Aging Project; DLPFC, Dorsolateral prefrontal cortex; CERAD, Consortium to Establish a Registry for Alzheimer’s Disease; Cogdx, Final consensus cognitive diagnosis; NCI, No cognitive impairment; MSBB, Mount Sinai Brain Bank; PHG, parahippocampal gyrus; IFG, Inferior frontal gyrus; STG, Superior temporal gyrus; CDR, Clinical Dementia Rating; NCD, No cognitive Deficits; TCX, Temporal cortex; Aβ, Amyloid-beta; CER, Cerebellum.

External validation of our gene expression signatures was performed using an independent dataset from the Aging, Dementia, and TBI study (GSE104687). The PHG signature successfully distinguished AD activity levels between control and severe AD groups in hippocampal PMB tissue across neuropathological and clinical measures (Table 2). However, external validation was restricted to these two regions due to unavailability of match PMB tissue to other regions. Although the TCX signature detected higher AD activity in the AD samples compared to control samples, the difference was not statistically significant.

**Table 2:** External validation of PHG and TCX signatures for predicting AD activity in matched PMB tissue from an independent cohort of aging, dementia and traumatic brain injury patients (GSE104687).

Region	Criteria	Comparison groups (control vs. AD)	Sample size (*N*)	Mean	Standard deviation (SD)	*p*-value	95% Confidence interval (CI)
PHG	Braak	≤ I	6	0.0252	0.0538	<0.05	[−0.900, −0.386]
		≥ V	8	0.668	0.306		
	CERAD	No AD/Possible AD	23	0.193	0.327	<0.05	[−0.711, −0.279]
		Definite AD	8	0.688	0.219		
	DSM-IV	“No Dementia”	28	0.216	0.336	<0.05	[−0.836, −0.618]
		“Alzheimer Disease Type”	11	0.551	0.348		
TCX	Braak	≤ I	6	0.134	0.302	0.301	[−0.614, 0.208]
		≥ V	7	0.337	0.372		
	CERAD	No AD/Possible AD	23	0.247	0.320	0.609	[−0.162, 0.272]
		Probable AD/Definite AD	16	0.192	0.333		
	DSM-IV	“No Dementia”	23	0.184	0.313	0.268	[−0.369, 0.108]
		“Alzheimer Disease Type”	16	0.315	0.337		

Mean predicted activity was compared between control and severe AD groups using Student’s *t*-tests. The PHG signature was validated across all measures, while TCX showed no significant differences. AD, Alzheimer’s Disease; CERAD, Consortium to Establish a Registry for Alzheimer’s Disease; PHG, parahippocampal gyrus; TCX, Temporal cortex; DSM-IV, Diagnostic and Statistical Manual of Mental Disorders-Fourth Edition.

### Influence of sociodemographic factors on disease progression

Signature predictions, along with genetic and sociodemographic factors, were evaluated for their influence on AD status (Supplementary Table 8). Signature predictions were the strongest AD predictors across all regions, except TCX where significance was limited by a small sample size (n = 23) after filtering for required meta-criteria.

In PHG, CER, and IFG, male sex was associated with a lower likelihood of AD compared to females, reaching significance in IFG (OR = 0.092, 95% CI [15.9, 1.49 × 10^4^], *p* < 0.001) but not in PHG or CER. In later-affected regions, APOE e3/e4 significantly increased AD likelihood compared to e2/e3 in DLPFC (OR = 9.58, 95% CI [1.89, 56.5], *p* < 0.01) and CER (OR = 27.9, 95% CI [14.4, 1.30 × 10^3^], *p* < 0.05). However, APOE genotype in TCX and age of death in DLPFC were not a statistically significant predictor of AD status.

Next, McFadden’s pseudo-R^2^ was calculated to assess model fit for predicting AD status (Supplementary Table 8). PHG showed the best model fit among significant predictors (very strong fit). TCX had the highest pseudo-R^2^ but should be interpreted with caution, as its predictors were not statistically significant in the GLM analysis. CER and IFG demonstrated strong fit, while STG and DLPFC had a moderate fit, suggesting external influences may still be contributing that is not explained by variables in the model. Despite this variation, all models demonstrated at least moderate fit, with LOOCV-confirmed AUCs ranging from 0.729 to 0.995 confirming their accuracy in predicting AD status.

### Unraveling shared gene networks and potential therapeutics

**Figure 3:**
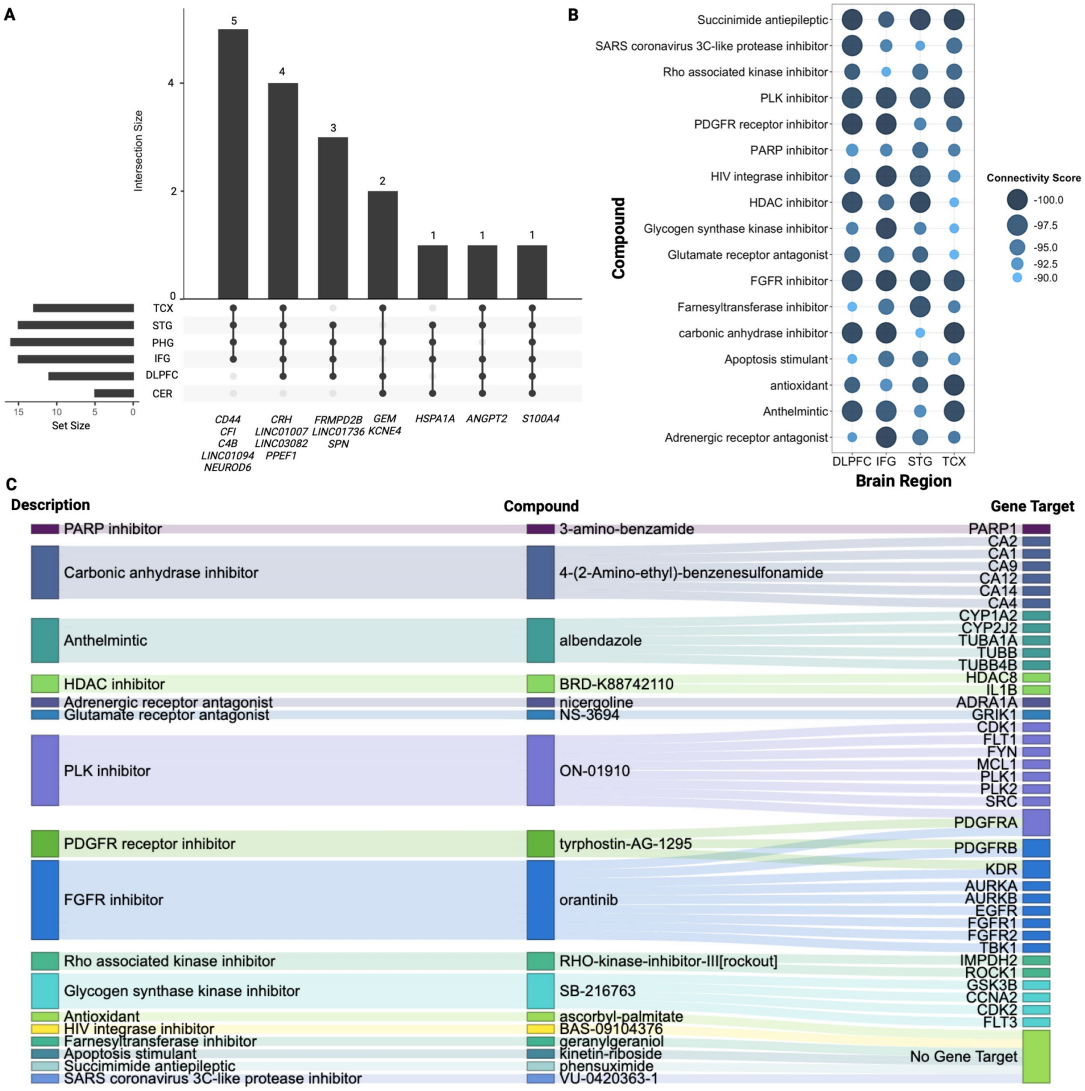
Signature gene analysis. **(A)** Upset plot displaying overlapping genes identified across four or more regions highlighting the number of genes shared between different combinations of regions. Vertical bars highlight the number of genes shared across the specified regions mentioned below, while horizontal bars represent how many genes from each individual region contribute to the overlapping gene sets. **(B)** Summary of CMAP analysis highlighting 17 compounds shared across TCX, STG, IFG, and DLPFC. Larger and darker blue bubbles indicate lower connectivity scores, representing a stronger reversal of the AD signature expression. **(C)** Sankey plot illustrating the drug class descriptions for the 17 shared compounds across TCX, STG, IFG, and DLPFC, along with their returned gene targets from CMAP.

To further understand molecular changes underlying dysregulation across regions, this analysis examined overlapping signature genes revealing notable commonalities, as shown in Figure 3A (Supplementary Figure 2; Supplementary Table 9). Specifically, the *S100A4* gene was consistently overexpressed in all six evaluated regions, indicating its potential universal role in the pathogenesis of AD. Additionally, five genes were found across five of the six regions including upregulation of *ANGPT2*, *LINC03082,* and downregulation of *CRH, LINC01007,* and *PPEF1*, suggesting their significant but less ubiquitous involvement. Next, fourteen other genes were found across four of the six brain regions with upregulation of *KCNE4, GEM, HSPA1A, SPN, LINC01736, CD44* and downregulation of *FRMPD2B* and *NEUROD6* (Figure 3; Supplementary Table 10). Notably, *CRH*, *ANGPT2*, *S100A4*, and *PPEF1* showed overlap with hub genes identified across regions further supporting their importance in dysregulation (Supplementary Table 10).

CMAP analysis was then conducted to identify potential drug repurposing candidates (Figures 3B,C). The results revealed the class of FGFR inhibitors shared across all regions except PHG and CER with orantinib emerging as a notable compound shared amongst these regions. As shown in Figure 3B, 17 compounds were found in common across TCX, STG, IFG, and DLPFC. Bromodomain inhibitors were another common class shared across TCX, STG, and DLPFC. While no specific bromodomain inhibitor was identified, knockdown of bromodomain targets like *BRD2* and *BRD4* reversed the AD expression captured in our signatures, suggesting their potential to reverse AD-specific effects in the TCX, STG and DLPFC. Lastly, the PHG returned only two compounds, while the CER yielded six, though neither region returned associated CMAP classes highlighting potentially tailored treatment approaches for PHG and CER.

### Comprehensive characterization of network dysregulation across brain regions

Pathway enrichment analysis also identified significant variability across regions (Supplementary Tables 11A–F). The STG had 663 enriched pathways, while the DLPFC had only 22. This stark contrast suggests possible differences in underlying biological or pathological mechanisms influencing AD progression and regional susceptibility. Despite this, extracellular matrix (ECM)-related processes were consistently upregulated across all regions, particularly in the extracellular matrix, external encapsulating structure, and collagen-containing ECM.

Similarly, network analysis identified functional modules revealing early-to-mid affected regions (PHG, TCX, STG) showing greater network dysregulation than mid-to-late affected regions (IFG, DLPFC, CER; Supplementary Table 12). For example, PHG had six functional clusters (MCODE score = 7.8), while DLPFC had two (MCODE score = 4.2), quantifying reduced dysregulation in later-affected regions. ClueGO functional enrichment further confirmed ECM upregulation across all regions and revealed downregulation of neuropeptide hormone activity and hormonal-related signaling in all regions except the CER. This may suggest a possible protective mechanism (Supplementary Tables 13A–F).

**Figure 4:**
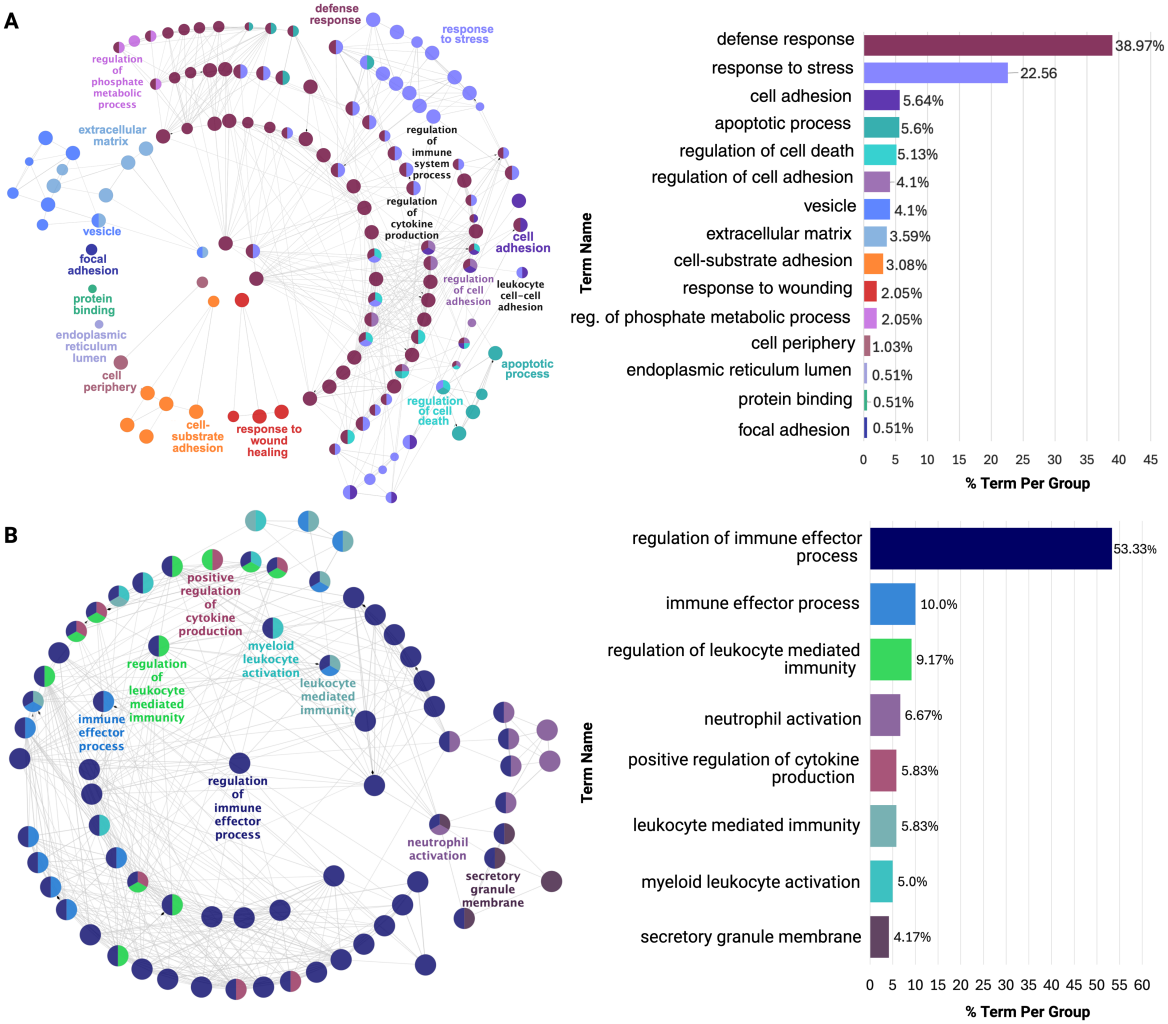
ClueGO functional enrichment analysis of dysregulated processes in earlier affected regions: **(A)** PHG and **(B)** STG. Both show overlap in stress and immune responses, but PHG is enriched for neuroinflammatory processes, while STG is enriched for immune effector processes. Nodes, color-coded by functional similarity, represent enriched pathways, with edges indicating gene-based functional relationships. The most significant terms are highlighted based on gene involvement, and the bar chart shows the proportion of each dysregulated term within the network.

Notably, the PHG and STG exhibited overlapping cytokine and immune defense responses, but PHG showed predominant neuroinflammatory upregulation, aligning with early AD pathology (Figure 4; Supplementary Tables 13A,B). In contrast, STG was upregulated for mainly immune effector activity, indicative of a later-stage neurodegenerative response. This may also be related to the upregulation of *CD44* gene expressed on immune cells observed across all regions except the DLPFC and CER (Wang et al., 2022). Next, in the TCX, downregulation of glutamate decarboxylase and upregulation of collagen fibril organization was revealed which could impair inhibitory neurotransmission, contributing to excitotoxicity, while altering the ECM structure may increase regional vulnerability (Supplementary Figure 3; Supplementary Table 13C; Ali et al., 2023).

**Figure 5:**
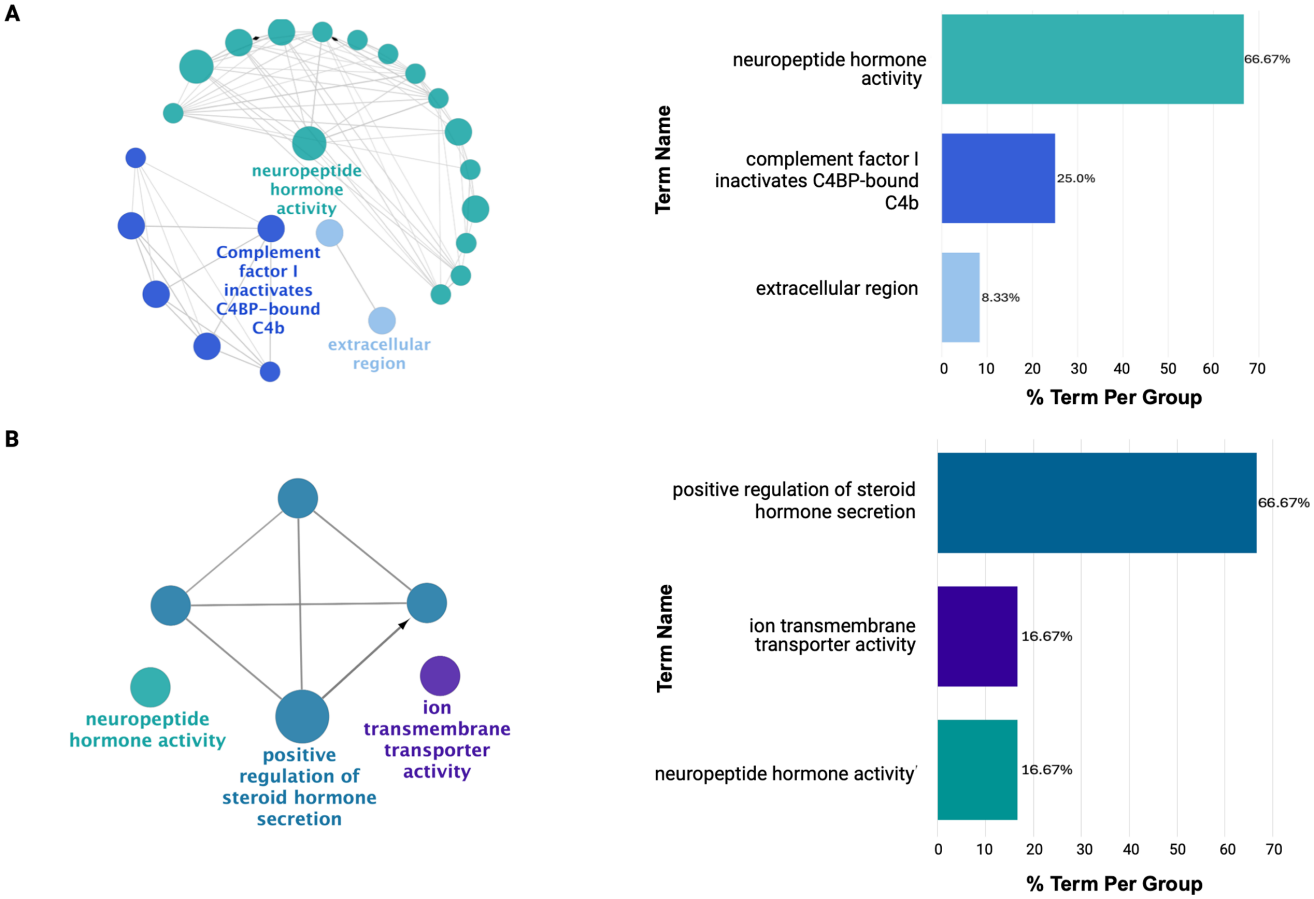
ClueGO functional enrichment analysis revealing dysregulated processes in mid-to-late affected regions **(A)** IFG and **(B)** DLPFC. Here, these regions show downregulation in hormonal-related signaling with limited diversity in dysregulated processes. For details on the network representation, node color-coding, and enrichment analysis, see Figure 4.

As shown in Figure 5, the IFG and DLPFC were predominately enriched for hormonal-related activity (Supplementary Tables 13D,E). This captured dysregulation from PMB tissue in mid-to-late affected regions may have highlighted downregulation of hormonal-related processes as a source of susceptibility for regions to AD pathology. This is supported by the consistent downregulation of hormonal signaling observed across all brain regions studied, except for the CER (Supplementary Table 13F). Finally, the CER showed heightened immune and inflammatory responses, including Toll-like receptor 4 upregulation, potentially driving increased chemotaxis and angiogenesis regulation (Supplementary Figure 4; Supplementary Table 13F).

## Discussion

Predictive gene expression signatures representative of AD for early-to-late affected regions were developed using PMB tissues. These signatures not only distinguished AD pathology from healthy controls but also correlated with key disease markers, including Aβ and tau burden, cognitive impairment (MMSE scores), and clinical diagnoses. Notably, our gene expression signatures were validated against Braak staging and CERAD scores. These metrics are widely used to quantify tau and amyloid pathology, respectively, and their alignment with our signatures reinforces the biological validity of the transcriptional patterns we identified as reflective of AD processes.

In addition to these findings, earlier affected regions, PHG and STG, exhibited the greatest dysregulation, reflected by the highest number of DEGs and functional clusters. From functional enrichment of these clusters, it was revealed that the PHG was dominated by neuroinflammatory processes while STG was enriched for immune responses, with both regions exhibiting overlapping processes. TCX followed with more limited dysregulation, primarily in ECM-related processes, which were upregulated across all regions, supported by the consistent upregulation of *S100A4*. IFG and DLPFC displayed even fewer molecular alterations, primarily affecting hormonal processes, a pattern observed across all regions except CER, where *CRH* downregulation was absent.

These findings align with the current understanding of the pattern of progression associated with AD pathology characterized by the accumulation of Aβ and tau phosphorylation first impacting regions such as the PHG followed by TCX and STG in temporal regions (Oostveen et al., 2021). As the disease progresses, pathology extends to mid-to-late affected regions, including IFG, eventually impacting DLPFC and CER in late stages (Oostveen et al., 2021). While this pattern of progression is well-characterized in AD, the molecular mechanisms underlying the differential susceptibility of specific regions remain elusive.

To characterize these molecular mechanisms, recent transcriptomic and single-cell studies have identified gene regulators consistent with our findings. One of the most interesting overlaps is CRH, which we found consistently downregulated in five of six regions, which was also shown to have reduced expression across multiple neuronal populations (Li and Larsen, 2023). This aligns with CRH’s role as a stress-related neuropeptide in early-affected regions like the entorhinal cortex (Li and Larsen, 2023). Additional work has also reported widespread CRH loss alongside glial overactivation and depletion of immune-regulatory neuropeptides in the parahippocampal and temporal cortices, mirroring the inflammatory profiles we observed in these same regions (Li et al., 2024). Beyond individual genes, our pathway enrichment results align with prior reviews identifying ECM remodeling as a driver of neuroinflammation and impaired Aβ clearance (Sun et al., 2021). Likewise, regional analyses of MSBB data support our findings by identifying the PHG as a hotspot of immune dysregulation and transcriptional activity during early AD progression (Neff et al., 2021). However, while these studies provide insights into AD’s molecular underpinnings, many fail to translate findings to clinical utility and are often limited to single regions or lacking correlation with disease severity or progression markers.

To address this gap, this study validated the predictive gene expression signatures from each region against established measures of AD pathology, as previously mentioned. The ability of these signatures to correlate with real-world measures of AD and distinguish AD activity in PMB tissue, highlights their potential clinical utility as diagnostic and prognostic tools, complementing current clinical assessments and improving our understanding of AD pathology.

Additionally, a hallmark of AD pathology is the persistent influence of Aβ and tau aggregates on affected brain regions throughout disease progression, driving the accumulation of molecular changes in these areas (Cai et al., 2023). This persistent dysregulation reinforces a cycle of neurodegeneration, with different brain regions undergoing various stages of disruption based on their involvement in AD progression. Our analysis captured this evolving molecular landscape, showing earlier affected regions such as the PHG and STG, showing extensive dysregulation exhibited by their number of DEGs and functional clusters, reflecting a greater extent of molecular changes. This aligns with their role as some of the first sites of Aβ and tau accumulation along with neurodegeneration (Aksman et al., 2023).

The predictive strength of our gene expression signatures in these regions further highlights their role as key markers of AD pathology, suggesting that the molecular changes are primarily driven by AD pathology. However, in the STG, some variability was captured in our gene expression but was not accounted for sociodemographic or genetic factors included in our predictive model. This may indicate that the variability captured in our signature reflects STG’s response to AD pathology in neighboring regions rather than intrinsic dysregulation alone. Supporting this, Mitra et al., (2024) found that STG gene expression patterns closely align with those of adjacent areas like the PHG and entorhinal cortex, suggesting that molecular changes in the STG are influenced by disease progression in these interconnected regions. Similarly, Wang et al., (2016) demonstrated that STG alterations align with broader pathological interactions in the brain, reinforcing the idea that its molecular variability reflects the regional spread of AD pathology. These findings suggest that while PHG and STG exhibit early molecular disruption, the STG’s gene expression changes may be shaped by its proximity to other affected regions.

Notably, these region-specific signatures exhibited similar dysregulation reflected by their overlap in affected processes. However, the PHG was predominantly enriched for neuroinflammatory processes while STG showed dysregulation in immune effector processes. This supports evidence that early neuroinflammation, particularly microglial activation, accelerates neurodegeneration in vulnerable cortical areas (Frigerio et al., 2019; Keren-Shaul et al., 2017). Additionally, these shared molecular alterations suggest immune activation in AD is not isolated but part of a broader interconnected response across affected cortical regions.

In contrast, the TCX, a mid-affected region in AD, exhibited limited dysregulation, primarily marked by downregulation of glutamate decarboxylase activity, consistent with decreased GABA and glutamate levels in AD patients. However, the interpretability of the TCX signature’s influence on AD status is constrained by inflated ORs, SE, and CIs in the predictive model due to the small sample size (n = 23), resulting in perfect separation within subcategories such as APOE genotype — this limitation affects the model’s predictive accuracy. While LOOCV confirmed the model’s ability to predict AD, the lack of statistically significant variables necessitates cautious interpretation. Moreover, the perfect separation of AD status based on APOE genotype suggests that APOE alone may drive prediction in the TCX region. This also explains why the TCX signature was not validated in the independent dataset, as the limited availability of representative control and AD samples compromised model robustness and external predictive performance.

Nevertheless, as AD spreads to mid-to-late affected regions such as IFG and DLPFC, a shift in dysregulation was observed exhibiting less diversity in dysregulated processes compared to early-affected regions. This likely reflects the later impact of AD pathology on these regions capturing their involvement at an earlier stage of molecular disruption compared to PHG and STG. This was highlighted by the predominant downregulation of hormone-related processes in both regions which has been implicated in early AD-related dysfunction. This may suggest these pathways serve as an early vulnerability to AD pathology.

Interestingly, our analysis suggests that AD may affect the IFG differently in males compared to females. This was highlighted from our predictive model that revealed males were less likely to develop AD than females, indicating higher AD susceptibility in females, consistent with other studies (Zhu et al., 2021). This may be due to estrogen decline, which has been linked to an increased risk of AD by disrupting hormone-related processes (Wang et al., 2024). These findings suggest that hormonal differences between sexes could play a key role in IFG vulnerability to AD.

Meanwhile, genetic predisposition played a larger role in DLPFC and CER, where APOE genotype significantly influenced development of AD supporting previous studies (Klyubin et al., 2022). Specifically, APOE e3/e4 carriers showed a heightened risk of AD compared to e2/e3, particularly in CER, where genetic influence appeared even more pronounced. This supports APOE e4 association with increased amyloid accumulation and neuroinflammatory responses in later-affected cortical and cerebellar regions (Klyubin et al., 2022).

While these findings reinforce that as AD progresses, molecular dysregulation shifts from being primarily tissue-specific to increasingly shaped by genetic and systemic influences. Common mechanisms were found across brain regions with upregulation of ECM remodeling, specifically in collagen-containing ECM components and structural organization. This suggests widespread alterations in brain architecture, further supported by the upregulation of *S100A4* across all regions in this study. This gene is a critical regulator of ECM turnover which modulates matrix degradation, remodeling, and cellular interactions, implicating it in pathological ECM remodeling (Gonzalez et al., 2020). This dysregulation likely leads to excessive collagen deposition, resulting in extracellular stiffening negatively impacting synaptic plasticity and compromising BBB integrity. Ultimately, dysregulation in these processes further exacerbate disease progression by impairing the clearance of Aβ and tau aggregates due to increased matrix rigidity. Alongside these structural changes, *ANGPT2*, a key regulator of blood vessel remodeling, was upregulated in all regions except the PHG (Van Hulle et al., 2024). Studies suggest *ANGPT2* is linked to vascular dysfunction in AD, contributing to BBB breakdown, inflammation, and impaired blood flow. Its widespread increase may reflect ongoing vascular instability, further compounding ECM-related disruptions (Van Hulle et al., 2024).

In parallel with the observed vascular and structural alterations, a consistent downregulation of neuropeptide hormone activity and hormonal regulatory processes was evident across all regions except for the CER. Notably, the gene *CRH* (corticotropin-releasing hormone), a key regulator of the hypothalamic–pituitary–adrenal (HPA) axis, exhibited widespread downregulation across all regions except the CER (Sukhareva, 2021). Given the role of *CRH* in stress adaptation and neuronal resilience, its downregulation suggests a weakened ability to regulate neuroinflammatory responses, leaving neurons more vulnerable to degeneration. This aligns with findings that HPA axis dysfunction in AD disrupts stress-response mechanisms, leading to hypothalamic atrophy causing increased susceptibility of amyloid-β accumulation (Sukhareva, 2021).

Interestingly, while many brain regions exhibit hormonal dysregulation in AD, the CER appears resistant, maintaining structural and functional integrity despite widespread cortical pathology. This resilience may stem from its preserved hormonal regulation, as studies suggest stable endocrine signaling contributes to neuroprotection (Radaghdam et al., 2021). In contrast, the hypothalamus, a region responsible for regulating hormones, metabolism, and stress responses, is one of the earliest regions affected in AD, showing dysfunction before cognitive impairment. As a key modulator of CRH, its breakdown disrupts HPA axis regulation, exacerbating stress-related neurodegeneration (Watermeyer et al., 2021). This early hormonal imbalance may destabilize other brain regions, increasing their susceptibility to AD pathology, while the CER’s preserved hormonal landscape could underlie its resistance.

Finally, the identification of region-specific therapeutic targets through CMAP analysis highlights promising avenues for AD intervention. Notably, FGFR inhibitors like orantinib, linked to TCX, STG, IFG, and DLPFC, target FGF signaling to potentially restore ECM integrity and reduce inflammation, aligning with disruptions observed in these regions (Alam et al., 2022). Bromodomain inhibitors were also shared across multiple regions which is a modulator of transcriptional regulation and neuroimmune activity, offering potential to counteract widespread epigenetic and inflammatory dysfunction (Rosenthal et al., 2024). While these drugs have been explored in other conditions, their potential in AD warrants further investigation, particularly considering their alignment with region-specific transcriptomic changes. Moreover, limited BBB permeability by these drugs remains a challenge, necessitating improved delivery strategies.

While this study provides key insights into region-specific molecular disruptions in AD, several limitations should be acknowledged. The use of PMB tissue primarily reflects late-stage disease changes, potentially overlooking early molecular alterations that could be better captured in longitudinal transcriptomic studies. Additionally, to ensure direct comparability across datasets, genes not shared across each study were excluded, which may have resulted in the omission of biologically significant signals. Another limitation was the signature generation and validation cohorts were constrained by publicly available gene expression and clinical data, and drug treatment information, limiting the ability to account for potential confounding factors such as comorbidities and drugs that may influence gene expression in PMB tissues. Variability in tissue collection and analysis across the three studies introduces methodological inconsistencies, impacting the precision of dysregulation quantification across regions. External validation of gene expression signatures was feasible only for the PHG and TCX due to the limited availability of matched PMB tissues from other regions, underscoring the need for larger, more diverse datasets to enhance robustness. Furthermore, CMAP-based drug predictions rely on transcriptional signatures rather than direct functional validation, necessitating further studies to confirm therapeutic relevance. Although our predictions offer promising therapeutic direction, their clinical applicability remains dependent on validation in early-stage or longitudinal cohorts to support utility in identifying at-risk individuals before clinical symptoms emerge. Despite these limitations, this study provides a strong foundation for understanding region-specific molecular alterations in AD and highlights potential therapeutic targets. Future studies leveraging PMB tissues from the same patients across multiple brain regions could offer a more comprehensive molecular landscape of AD pathogenesis.

## Conclusion

This study identified predictive gene expression signatures that correlate with real-world AD pathology, providing reliable biomarkers that could complement current diagnostic approaches. Using these signatures, dysregulated processes shared across regions were highlighted as potential drivers for regional vulnerability revealing potential therapeutic targets while quantifying regional impact of AD across brain regions. While our gene expression signatures were derived from PMB tissues, future studies should investigate whether these signatures—or functionally relevant subsets—can be detected in blood-derived RNA samples.

## Data Availability

Bulk RNA-seq gene count data of postmortem brain tissue used in this study are publicly available through the AD Knowledge Portal (Synapse). Additional bulk RNA-seq expression data were obtained from the Aging, Dementia, and Traumatic Brain Injury Study (NCBI-GEO, Accession GSE104687). The analysis code is available at: https://github.com/ashleyduche/AD_Predictive_Signatures.

## References

[ref1] Alam R. Mrad Y. Hammoud H. Saker Z. Fares Y. Estephan E.. (2022). New insights into the role of fibroblast growth factors in Alzheimer’s disease. Mol. Biol. Rep. 49, 1413–1427. doi: 10.1007/s11033-021-06890-0, PMID: 34731369

[ref2] Ali A. B. Islam A. Constanti A. (2023). The fate of interneurons,GABAAreceptor sub‐types and perineuronal nets in Alzheimer's disease. Brain Pathol. 33:13129. doi: 10.1111/bpa.13129, PMID: 36409151 PMC9836378

[ref3] Allen M. Carrasquillo M. M. Funk C. Heavner B. D. Zou F. Younkin C. S.. (2016). Human whole genome genotype and transcriptome data for Alzheimer’s and other neurodegenerative diseases. Sci Data 3:160089. doi: 10.1038/sdata.2016.89, PMID: 27727239 PMC5058336

[ref9001] Aksman L. M. Oxtoby N. P. Scelsi M. A. Wijeratne P. A. Young A. L. Alves I. L.. (2023). A data-driven study of Alzheimer’s disease related amyloid and tau pathology progression. Brain, 146. 10.1093/brain/awad232 PMC10690020 37433038

[ref4] Bader G. D. Hogue C. W. (2003). An automated method for finding molecular complexes in large protein interaction networks. BMC Bioinform. 4, 1–4. doi: 10.1186/1471-2105-4-2, PMID: 12525261 PMC149346

[ref5] Bennett D. A. Buchman A. S. Boyle P. A. Barnes L. L. Wilson R. S. Schneider J. A. (2018). Religious orders study and rush memory and aging project. J Alzheimer's Dis 64, S161–S189. doi: 10.3233/JAD-179939, PMID: 29865057 PMC6380522

[ref6] Bindea G. Mlecnik B. Hackl H. Charoentong P. Tosolini M. Kirilovsky A.. (2009). Cluego: a Cytoscape plug-in to decipher functionally grouped gene ontology and pathway annotation networks. Bioinformatics 25, 1091–1093. doi: 10.1093/bioinformatics/btp101, PMID: 19237447 PMC2666812

[ref7] Cai Y. Du J. Li A. Zhu Y. Xu L. Sun K.. (2023). Initial levels of β-amyloid and tau deposition have distinct effects on longitudinal tau accumulation in Alzheimer’s disease. Alzheimer's Res Ther 15:15. 36750884 10.1186/s13195-023-01178-w PMC9903587

[ref8] Chin C.-H. Chen S.-H. Wu H.-H. Ho C.-W. Ko M.-T. Lin C.-Y. (2014). cytoHubba: identifying hub objects and sub-networks from complex interactome. BMC Syst. Biol. 8:S11. doi: 10.1186/1752-0509-8-S4-S11, PMID: 25521941 PMC4290687

[ref8001] Duche A. (2025). Available online at: https://BioRender.com/tqixkvc

[ref9002] Frigerio C. S. Wolfs L. Fattorelli N. Thrupp N. Voytyuk I. Schmidt I.. (2019). The Major Risk Factors for Alzheimer’s Disease: Age, Sex, and Genes Modulate the Microglia Response to Aβ Plaques. Cell Reports, 27. 10.1016/j.celrep.2019.03.099 PMC7340153 31018141

[ref9] Gonzalez L. L. Garrie K. Turner M. D. (2020). Role of S100 proteins in health and disease. Biochim. Biophys. Acta Molecul. Cell Res. 1867:118677. doi: 10.1016/j.bbamcr.2020.118677, PMID: 32057918

[ref9003] Keren-Shaul H. Spinrad A. Weiner A. Matcovitch-Natan O. Dvir-Szternfeld R. Ulland T. K.. (2017). A Unique Microglia Type Associated with Restricting Development of Alzheimer’s Disease. Cell, 169. 10.1016/j.cell.2017.05.018 28602351

[ref10] Klyubin I. Ondrejcak T. Hu N.-W. Rowan M. J. (2022). Glucocorticoids, synaptic plasticity and Alzheimer's disease. Curr. Opin. Endocr. Metab. Res. 25:100365. doi: 10.1016/j.coemr.2022.100365

[ref11] Kodam P. Sai Swaroop R. Pradhan S. S. Sivaramakrishnan V. Vadrevu R. Kodam P.. (2023). Integrated multi-omics analysis of Alzheimer’s disease shows molecular signatures associated with disease progression and potential therapeutic targets. Sci. Rep. 13:13. 36879094 10.1038/s41598-023-30892-6 PMC9986671

[ref12] Kuhn M. (2008). Building predictive models in R using the caret package. J. Stat. Softw. 28, 1–26. doi: 10.18637/jss.v028.i0527774042

[ref13] Li M. Flack N. Larsen P. A. (2024). Multifaceted role of specialized neuropeptide-intensive neurons on the selective vulnerability to Alzheimer’s disease in the human brain. Biomolecules 14:1518. doi: 10.3390/biom14121518, PMID: 39766225 PMC11673071

[ref14] Li M. Larsen P. A. (2023). Single-cell sequencing of entorhinal cortex reveals widespread disruption of neuropeptide networks in Alzheimer's disease. Alzheimers Dement. 19, 3575–3592. doi: 10.1002/alz.12979, PMID: 36825405

[ref15] Love M. I. Huber W. Anders S. (2014). Moderated estimation of fold change and dispersion for Rna-seq data with Deseq2. Genome Biol. 15:550. doi: 10.1186/s13059-014-0550-8, PMID: 25516281 PMC4302049

[ref9004] Mitra S. Bp K. CR S. Saikumar N. V. Philip P. Narayanan M.. (2024). Alzheimer’s disease rewires gene coexpression networks coupling different brain regions. npj Systems Biology and Applications, 10, 1–10. 38724582 10.1038/s41540-024-00376-y PMC11082197

[ref16] Mock C. Teylan M. Beecham G. Besser L. Cairns N. J. Crary J. F.. (2020). The utility of the National Alzheimer's coordinating center's database for the rapid assessment of evolving Neuropathologic conditions. Alzheimer Dis. Assoc. Disord. 34, 105–111. doi: 10.1097/WAD.0000000000000380, PMID: 32304374 PMC7242145

[ref17] Monteiro A. R. Barbosa D. J. Remião F. Silva R. (2023). Alzheimer’s disease: insights and new prospects in disease pathophysiology, biomarkers and disease-modifying drugs. Biochem. Pharmacol. 211:115522. doi: 10.1016/j.bcp.2023.115522, PMID: 36996971

[ref18] Montojo J. Zuberi K. Rodriguez H. Kazi F. Wright G. Donaldson S. L.. (2010). Genemania Cytoscape plugin: fast gene function predictions on the desktop. Bioinformatics 26, 2927–2928. doi: 10.1093/bioinformatics/btq562, PMID: 20926419 PMC2971582

[ref19] Neff R. A. Wang M. Vatansever S. Guo L. Ming C. Wang Q.. (2021). Molecular subtyping of Alzheimer’s disease using RNA sequencing data reveals novel mechanisms and targets. Sci. Adv. 7:eabb5398. doi: 10.1126/sciadv.abb5398, PMID: 33523961 PMC7787497

[ref20] Oostveen W. M. V. Lange E. C. M. D. Van Oostveen W. M. De Lange E. C. M. (2021). Imaging techniques in Alzheimer’s disease: a review of applications in early diagnosis and longitudinal monitoring. Int. J. Mol. Sci. 22, 2110–2122. doi: 10.3390/ijms22042110, PMID: 33672696 PMC7924338

[ref21] Radaghdam S. Karamad V. Nourazarian A. Shademan B. Khaki-Khatibi F. Nikanfar M. (2021). Molecular mechanisms of sex hormones in the development and progression of Alzheimer's disease. Neurosci. Lett. 764:136221. doi: 10.1016/j.neulet.2021.136221, PMID: 34500000

[ref22] Raudvere U. Kolberg L. Kuzmin I. Arak T. Adler P. Peterson H.. (2019). G:profiler: a web server for functional enrichment analysis and conversions of gene lists (2019 update). Nucleic Acids Res. 47, W191–W198. doi: 10.1093/nar/gkz369, PMID: 31066453 PMC6602461

[ref23] Rosenthal Z. C. Fass D. M. Payne N. C. She A. Patnaik D. Hennig K. M.. (2024). Epigenetic modulation through bet bromodomain inhibitors as a novel therapeutic strategy for progranulin-deficient frontotemporal dementia. Sci. Rep. 14:9064. doi: 10.1038/s41598-024-59110-7, PMID: 38643236 PMC11032351

[ref24] Shannon P. Markiel A. Ozier O. Baliga N. S. Wang J. T. Ramage D.. (2003). Cytoscape: a software environment for integrated models of biomolecular interaction networks. Genome Res. 13, 2498–2504. doi: 10.1101/gr.1239303, PMID: 14597658 PMC403769

[ref25] Shen Y. Rahman M. Piccolo S. R. Gusenleitner D. El-Chaar N. N. Cheng L.. (2015). Assign: context-specific genomic profiling of multiple heterogeneous biological pathways. Bioinformatics 31, 1745–1753. doi: 10.1093/bioinformatics/btv031, PMID: 25617415 PMC4443674

[ref26] Subramanian A. Narayan R. Corsello S. M. Peck D. D. Natoli T. E. Lu X.. (2017). A next generation connectivity map: L1000 platform and the first 1,000,000 profiles. Cell 171, 1437–1452.e17. doi: 10.1016/j.cell.2017.10.049, PMID: 29195078 PMC5990023

[ref27] Sukhareva E. V. (2021). The role of the corticotropin-releasing hormone and its receptors in the regulation of stress response. Vavilov J. Genet. Breed. 25, 216–223. doi: 10.18699/VJ21.025, PMID: 34901719 PMC8627883

[ref28] Sun Y. Xu S. Jiang M. Liu X. Yang L. Bai Z.. (2021). Role of the extracellular matrix in Alzheimer’s disease. Front. Aging Neurosci. 13:707466. doi: 10.3389/fnagi.2021.707466, PMID: 34512308 PMC8430252

[ref29] Team R. C. (2024). R: A language and environment for statistical computing.

[ref30] Truong Q. C. Cervin M. Choo C. C. Numbers K. Bentvelzen A. C. Kochan N. A.. (2024). Examining the validity of the mini-mental state examination (MMSE) and its domains using network analysis. Psychogeriatrics 24, 259–271. doi: 10.1111/psyg.13069, PMID: 38131467 PMC11577997

[ref31] Van Hulle C. Ince S. Okonkwo O. C. Bendlin B. B. Johnson S. C. Carlsson C. M.. (2024). Elevated Csf angiopoietin-2 correlates with blood-brain barrier leakiness and markers of neuronal injury in early Alzheimer’s disease. Transl. Psychiatry 14:14. 38182581 10.1038/s41398-023-02706-w PMC10770135

[ref32] Wang X. Feng S. Deng Q. Wu C. Duan R. Yang L. (2024). The role of estrogen in Alzheimer’s disease pathogenesis and therapeutic potential in women. Mol. Cell. Biochem. 480, 1983–1998. doi: 10.1007/s11010-024-05071-4, PMID: 39088186

[ref9005] Wang M. Roussos P. Mckenzie A. Zhou X. Kajiwara Y. Brennand K. J.. (2016). Integrative network analysis of nineteen brain regions identifies molecular signatures and networks underlying selective regional vulnerability to Alzheimer’s disease. Genome Medicin, 8, 1–8. 10.1186/s13073-016-0355-3 PMC5088659 27799057

[ref33] Wang X. Wang D. Su F. Li C. Chen M. (2022). Immune abnormalities and differential gene expression in the hippocampus and peripheral blood of patients with Alzheimer’s disease. Annal. Transl. Med. 10:29. doi: 10.21037/atm-21-4974, PMID: 35282083 PMC8848377

[ref34] Wang C. Yin R. Dai J. Gu Y. Cui S. Ma H.. (2018). Whole-genome sequencing reveals genomic signatures associated with the inflammatory microenvironments in Chinese NSCLC patients. Nat. Commun. 9:2054. doi: 10.1038/s41467-018-04492-2, PMID: 29799009 PMC5967326

[ref35] Watermeyer T. Robb C. Gregory S. Udeh-Momoh C. (2021). Therapeutic implications of hypothalamic-pituitaryadrenal-axis modulation in Alzheimer’s disease: a narrative review of pharmacological and lifestyle interventions. Front. Neuroendocrinol. 60:100877. doi: 10.1016/j.yfrne.2020.100877, PMID: 33045258

[ref36] Zeileis A. Kleiber C. Jackman S. Zeileis A. Kleiber C. Jackman S. (2008). Regression models for count data in R. J. Stat. Softw. 27:8. doi: 10.18637/jss.v027.i08

[ref37] Zhang Y. Parmigiani G. Johnson W. E. (2020). ComBat-seq: batch effect adjustment for Rna-seq count data. Nar. Genom. Bioinform. 2:78. doi: 10.1093/nargab/lqaa078, PMID: 33015620 PMC7518324

[ref9006] Zhu D. Montagne A. Zhao Z. Zhu D. Montagne A. Zhao Z. (2021). Alzheimer’s pathogenic mechanisms and underlying sex difference. Cellular and Molecular Life Sciences, 78, 11–78. 10.1007/s00018-021-03830-w PMC8720296 33844047

