# Supplementary material for: Predictive gene expression signatures for Alzheimer’s disease using post-mortem brain tissue
Source: Front Aging Neurosci. 2025 Oct 2;17:1591946. doi: 10.3389/fnagi.2025.1591946 (PMC12528040; doi:10.3389/fnagi.2025.1591946)
Supplement: Supplementary file 1 [file Data_Sheet_1.pdf]

Supplementary Figures **2**
Supplementary Figure 1. 3
Supplementary Figure 2. 4
Supplementary Figure 4. 5

Supplementary Tables **6**
Supplementary Table 1. Description of dataset-specific metadata. 6
Supplementary Table 2. Description of data filtering steps. 7
Supplementary Table 3. Description of control and AD training characteristics. 8
Supplementary Table 4. Cohort demographics stratified by diagnosis (Control vs. Alzheimer's Disease). 8
Supplementary Table 5. Summary of data for DGE analysis. 10
Supplementary Table 6. Metadata categories across datasets. 10
Supplementary Table 7A. 176 DEGs identified for PHG 11
Supplementary Table 7B. 127 DEGs identified for the STG 16
Supplementary Table 7C. 53 DEGs identified for the TCX 19
Supplementary Table 7D. 44 DEGs identified for the IFG 20
Supplementary Table 7E. 53 DEGs identified for the DLPFC 21
Supplementary Table 7F. 55 DEGs identified for the CER 23
Supplementary Table 8. Summary of the best-fit GLMs. 24
Supplementary Table 9. Signature gene overlap. 25
Supplementary Table 10. Identification of hub genes across region. 32
Supplementary Table 11A. PHG significant enrichment terms from g:Profiler. 32
Supplementary Table 11B. STG significant enrichment terms from g:Profiler. 39
Supplementary Table 11C. TCX significant enrichment terms from g:Profiler. 53
Supplementary Table 11D. IFG significant enrichment terms from g:Profiler. 56
Supplementary Table 11E. DLPFC significant enrichment terms from g:Profiler. 58
Supplementary Table 11F. CER significant enrichment terms from g:Profiler. 59
Supplementary Table 12. Identification of functional modules for each region. 61
Supplementary Table 13A. ClueGO functional enrichment analysis of PHG. 62
Supplementary Table 13B. ClueGO functional enrichment analysis of STG. 65
Supplementary Table 13C. ClueGO functional enrichment analysis of TCX. 68
Supplementary Table 13D. ClueGO functional enrichment analysis of IFG. 68
Supplementary Table 13E. ClueGO functional enrichment analysis of DLPFC. 69
Supplementary Table 13F. ClueGO functional enrichment analysis of CER. 69

Supplementary Figures

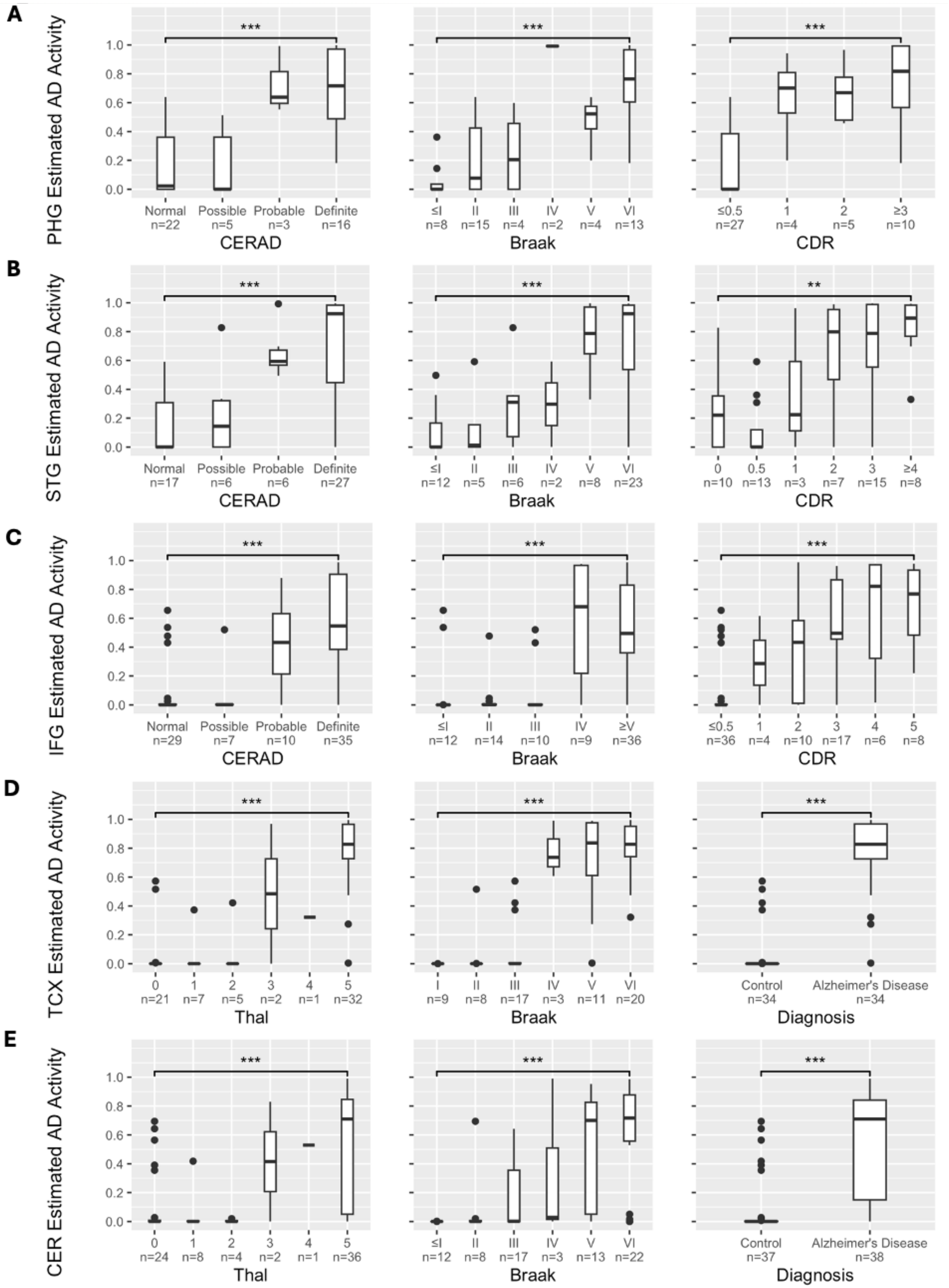

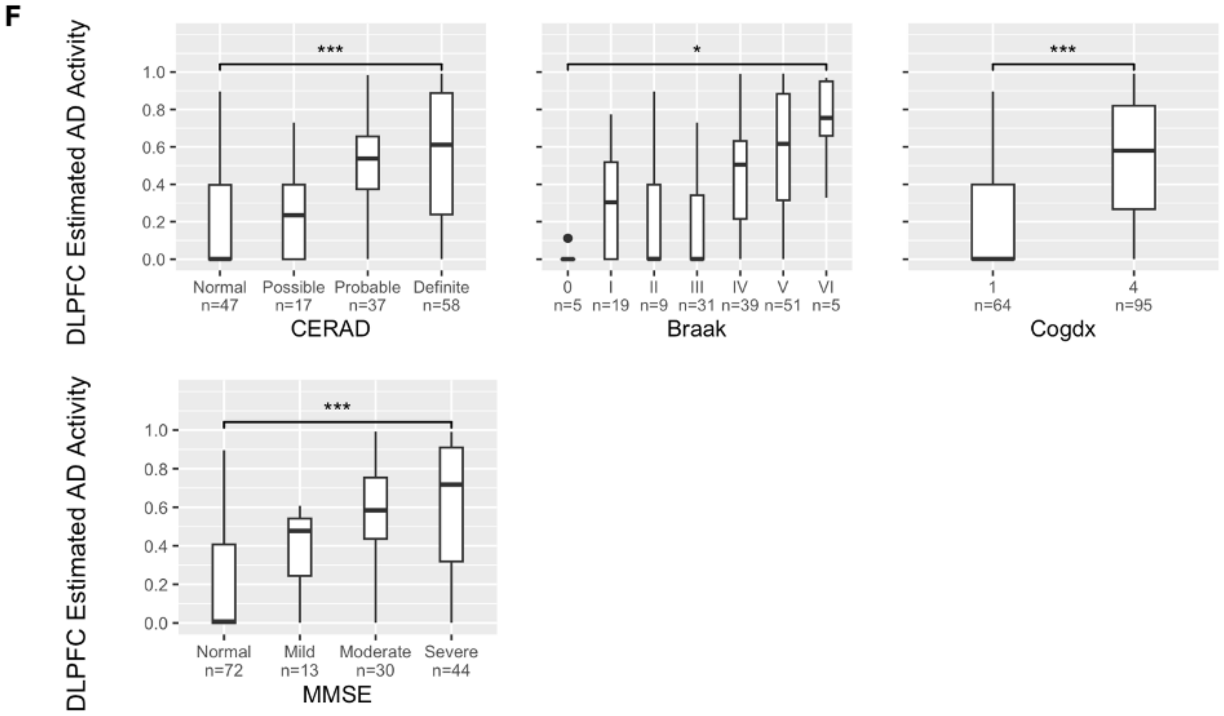

Significance codes: *** p < 0.001, ** p < 0.01, * p < 0.05

Supplementary Figure 1. Validation of gene expression signature predictions, displayed as boxplots, for the **(A)** PHG **(B)** STG **(C)** IFG **(D)** TCX **(E)** CER and **(F)** DLPFC across established neuropathological and clinical AD markers provided from each study. Signature predictions are plotted on y-axis predicting AD activity in samples which are compared to their known AD status across meta categories on the x-axis. Student's t-test were used to validate that mean predicted AD activity was significantly higher in severe AD samples compared to controls, across all regions and measures of AD.

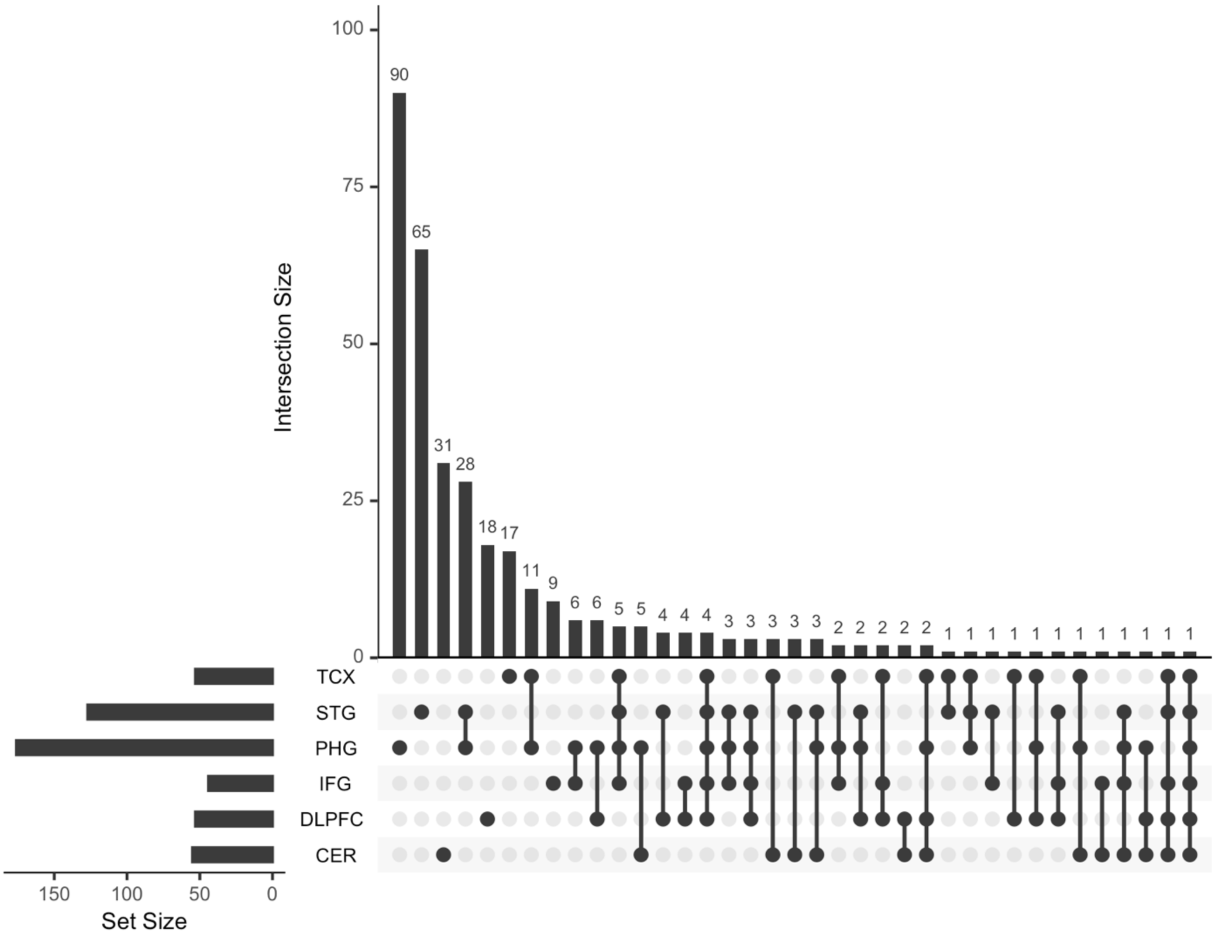

Supplementary Figure 2. Upset plot displaying overlapping genes identified across three or more regions highlighting the number of genes shared between different combinations of regions. Vertical bars highlight the number of genes shared across the specified regions mentioned below, while horizontal bars represent how many genes from each individual region contribute to the overlapping gene sets.

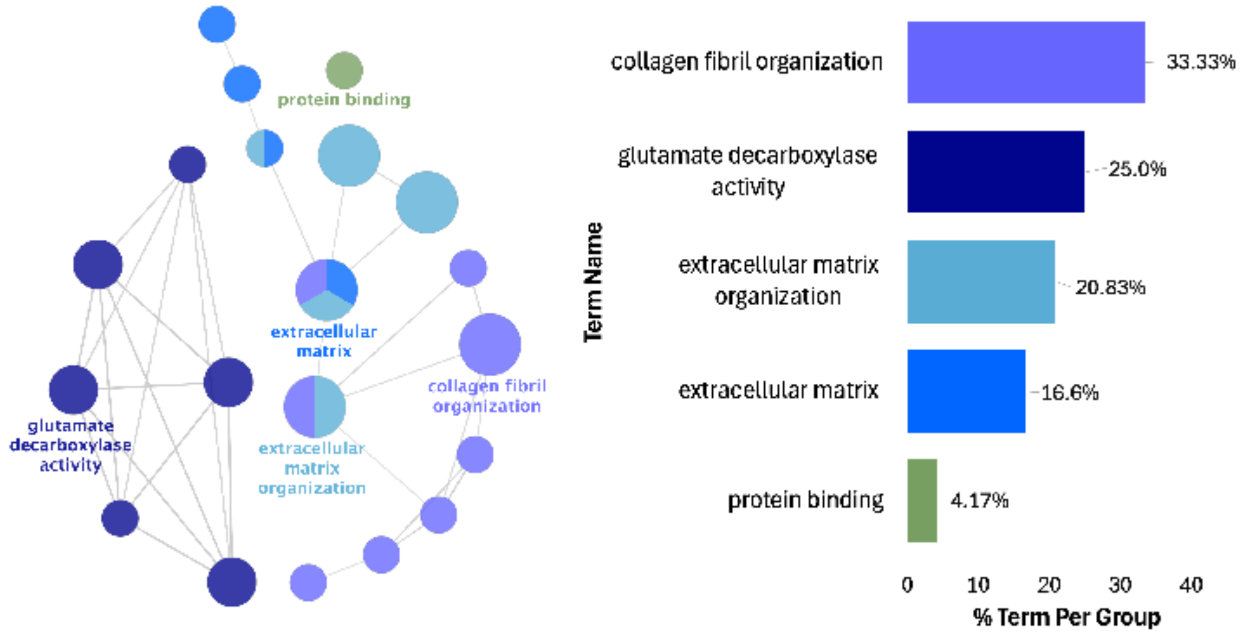

Supplementary Figure 3. ClueGO functional enrichment analysis of dysregulated processes in the TCX. Nodes, color-coded by functional similarity, represent enriched pathways, with edges indicating gene-based functional relationships. The most significant terms are highlighted based on gene involvement, and the bar chart shows the proportion of each dysregulated term within the network

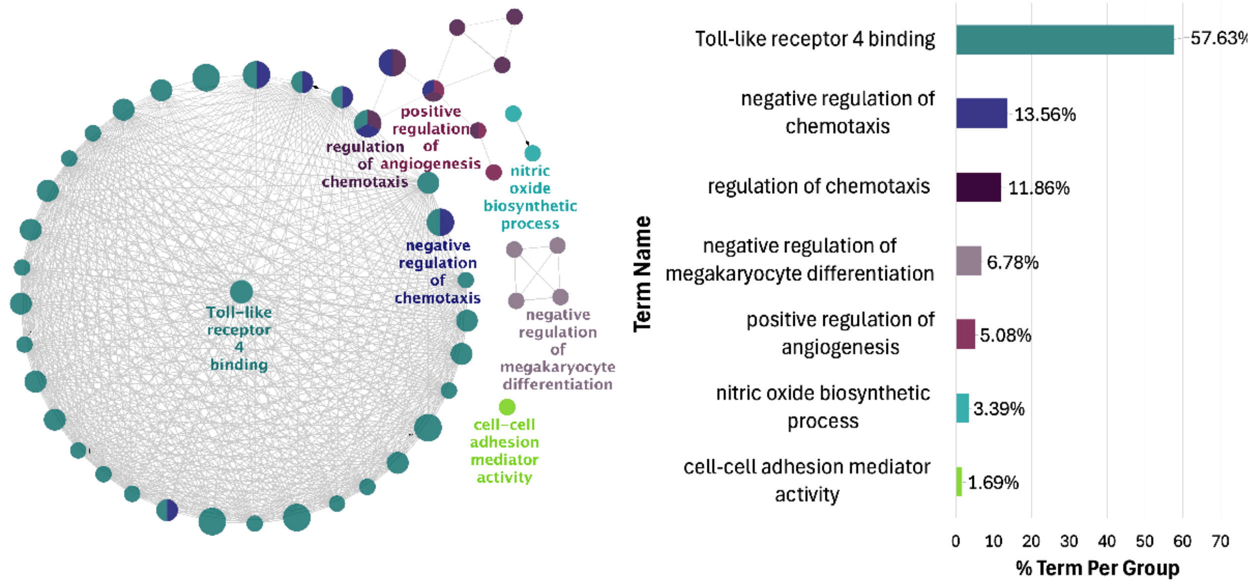

Supplementary Figure 4. ClueGO functional enrichment analysis of dysregulated processes in the CER. Nodes, color-coded by functional similarity, represent enriched pathways, with edges indicating gene-based functional relationships. The most significant terms are highlighted based on gene involvement, and the bar chart shows the proportion of each dysregulated term within the network

Supplementary Tables

Supplementary Table 1. Description of dataset-specific metadata. From each study (ROSMAP, MSBB, Mayo, and GSE104687), neuropathological and clinical metadata were used to define control and AD training groups for DGE analysis and signature generation using ASSIGN, as well as DGE signature validation on independent datasets. Neuropathological measures assessed postmortem included Braak stages (NFT severity), CERAD scores (neuritic plaque density), Thal phases (amyloid plaque distribution), and a combined Diagnosis variable from Mayo based on Braak stages and CERAD scores. Clinical variables assessing cognitive function during life were also incorporated from each study to define training groups.

Filter	Variable Name	Assessment Type	Data Variable	Definition
Braak Stages	Braak	Neuropathological	0	No NFTs
			I	NFT confined to entorhinal region
			II	NFT confined to entorhinal region
			III	NFT involvement in limbic regions & hippocampus
			IV	NFT involvement in limbic regions & hippocampus
			V	NFT moderate to severe neocortical involvement
			VI	NFT moderate to severe neocortical involvement
CERAD Scores	CERAD	Neuropathological	No AD	None: No neuritic plaques
			Possible AD	Sparse: Occasional neuritic plaques
			Probable AD	Moderate: Neuritic plaques in 1 or more neocortical regions
			Definite AD	Frequent: Neuritic plaques in 1 or more neocortical regions
Thal Phases	Thal	Neuropathological	No Aβ plaques	No Aβ plaques
			Isocortical	Aβ plaques present in isocortical (neocortical) regions
			Allocortical/limbic	Aβ plaques extend to allocortical (limbic) areas
			Subcortical	Aβ plaques spread into subcortical structures
			Mid-brain medulla Oblongata	Aβ plaques involve the midbrain and medulla oblongata
			Pons and cerebellum	Aβ plaques reach the pons and cerebellum
Diagnosis	Cogdx (ROSMAP)	Clinical	NCI	No cognitive impairment (No impaired domains)
			MCI	Mild Cognitive Impairment (1 impaired domain), no other cause of CI
			MCI+	Mild Cognitive Impairment (1 impaired domain), another cause of CI
			AD	Alzheimer's dementia (NINCDS-ADRDA Probable AD), no other cause of CI
			AD+	Alzheimer's dementia (NINCDS-ARDA Possible AD), another cause of CI
			Other dementia	Other primary cause of dementia
	CDR (MSBB)	Clinical	NCD	No cognitive deficits (CDR = 0)
			Questionable	Questionable dementia (CDR = 0.5)
			Mild	Mild dementia (CDR = 1)
			Moderate	Moderate dementia (CDR = 2)
			Severe to terminal	Severe to terminal dementia (CDR = 3)
			Severe to terminal	Severe to terminal dementia (CDR = 4)
			Severe to terminal	Severe to terminal dementia (CDR = 5)
	Diagnosis (Mayo)	Neuropathological	"Control"	No other pathological diagnosis, Braak ≤III, CERAD of No AD/ Possible AD
			"Alzheimer Disease"	Alzheimer dementia diagnosis (NINCDS-ADRDA), Braak ≥ 4, neuritic plaques present
	DSM-IV Clinical Diagnosis (GSE104687)	Clinical	"No Dementia"	No cognitive decline or impairment
			"Alzheimer's Disease Type"	Decline in memory and learning and in at least one other cognitive domain

NFTs: Neurofibrillary tangles; CERAD: Consortium to Establish a Registry for Alzheimer's Disease; AD: Alzheimer's Disease; Aβ: Amyloid -Beta; Cogdx: Final consensus cognitive diagnosis; CDR: Clinical Dementia Rating; NINCDS – ADRDA: National Institute of Neurological and Communicative Disorders and Stroke and the Alzheimer's Disease and Related Disorders Association

Supplementary Table 2. Description of data filtering steps. Each dataset underwent standardized processing, including filtering to retain only genes present across all datasets, applying RIN thresholds to exclude low-quality samples, and removing samples with missing metadata or those not relevant to Alzheimer's or control groups. Outliers were identified through PCA and subsequently removed, and batch effects were corrected where necessary to ensure consistency across datasets. This was performed on datasets used for the development of DGE signatures across all regions, as well as for an independent dataset (GSE104687) used for external validation of two regional DGE signatures.

Study	Brain Region	RIN Filter	Dataset Processing Steps
DGE signature development			
ROSMAP	DLPFC	> 6.5	1. Filter dataset to include genes common across all datasets.<br>2. Exclude samples missing meta data for CERAD scores, Braak stages, and Cogdx criteria.<br>3. Remove outlier samples and batches based on PCA.
MSBB	PHG	> 6.5	1. Filter dataset to include genes common across all datasets.<br>2. Exclude samples missing meta data for CERAD scores, Braak stages, CDR, and Plaque Mean.<br>3. Remove outlier samples based on PCA.<br>4. Performed batch correction adjusting for sequencing batches using ComBat-seq from sva package (version 3.50.0).
	IFG	> 6.5	1. Filter dataset to include genes common across all datasets.<br>2. Exclude samples missing meta data for CERAD scores, Braak stages, CDR.<br>3. Remove outlier samples based on PCA.
	STG	> 6.5	1. Filter dataset to include genes common across all datasets.<br>2. Exclude samples missing meta data for CERAD scores, Braak stages, CDR.
Mayo	TCX	> 7	1. Filter dataset to include genes common across all datasets.<br>2. Exclude samples missing meta data for Thal and PMI.
	CER	> 7	1. Filter dataset to include genes common across all datasets.<br>2. Exclude samples missing meta data for Thal and PMI.
External DGE signature validation (Independent dataset)			
GSE104687	PHG	> 5	1. Filter dataset to include genes found in the datasets used to developed DGE signatures.<br>2. Exclude samples reported to have TBI.<br>3. Include samples reported to have "Alzheimer's Disease Type" or "No dementia".
	TCX	> 5	1. Filter dataset to include genes found in the datasets used to developed DGE signatures.<br>2. Exclude samples reported to have TBI.<br>3. Include samples reported to have "Alzheimer's Disease Type" or "No dementia".

RIN: RNA Integrity Number; ROSMAP: The Religious Orders Study and Memory and Aging Project; DLPFC: Dorsolateral prefrontal cortex; MSBB: Mount Sinai Brain Bank; PHG: parahippocampal gyrus; IFG: Inferior frontal gyrus; STG: Superior temporal gyrus; TCX: Temporal cortex; CER: Cerebellum; CERAD: Consortium to Establish a Registry for Alzheimer's Disease; Cogdx: Final consensus cognitive diagnosis; PCA: Principal Component Analysis; CDR: Clinical Dementia Rating; PMI: Postmortem interval; DGE: Differential Gene Expression TBI: Traumatic Brain Injury.

Supplementary Table 3. Description of control and AD training characteristics. Control and AD training groups for each brain region were defined for DGE analysis, independently. This included using a combination of filters including Braak stages, CERAD scores, and clinical diagnosis variables for ROSMAP and MSBB datasets while and Mayo datasets applied only neuropathological data (Braak stages and CERAD scores) to define the diagnosis variable as shown below.

Study	Brain Region	Training Filters	Control Criteria	AD Criteria
ROSMAP	DLPFC	Braak stages	≤ III	≥ IV
		CERAD scores	No AD/ Possible AD	Probable AD/ Definite AD
		Cogdx	NCI	AD
MSBB	PHG, IFG, and STG	Braak stages	≤ III	≥ IV
		CERAD scores	No AD/ Possible AD	Probable AD/ Definite AD
		CDR	NCD/ Questionable	Mild/ Moderate/ Severe to terminal
Mayo	TCX and CER	Braak stages	≤ III	≥ IV
		Diagnosis	"Control"	"Alzheimer Disease"

AD: Alzheimer's Disease; ROSMAP: The Religious Orders Study and Memory and Aging Project; DLPFC: Dorsolateral prefrontal cortex; CERAD: Consortium to Establish a Registry for Alzheimer's Disease; Cogdx: Final consensus cognitive diagnosis; NCI: No cognitive impairment; MSBB: Mount Sinai Brain Bank; PHG: parahippocampal gyrus; IFG: Inferior frontal gyrus; STG: Superior temporal gyrus; CDR: Clinical Dementia Rating; NCD: No cognitive Deficits; TCX: Temporal cortex; CER: Cerebellum

Supplementary Table 4. Cohort demographics stratified by diagnosis (Control vs. Alzheimer's Disease).

Region	Variable	Control	AD	p-value
DLPFC	Sample Size, n	64	95	NA
	Age of Death (years)	85.1 (78.6-89.1)	90.0 (87.9-90.0)	< 0.0001 [1]
	Sex, n (%)			0.060 [2]
	Male	28 (43.8)	27 (28.4)	
	Female	36 (56.3)	68 (71.6)	
	Ethnicity, n (%)			1.000 [3]
	White	63 (98.4)	94 (98.9)	
	Other	1 (1.56)	1 (1.10)	
	APOE Genotype, n (%)			< 0.001 [3]
	e2e3	9 (14.1)	6 (6.31)	
	e2e4	1 (1.56)	2 (2.11)	
	e3e3	50 (78.1)	52 (54.7)	
	e3e4	4 (6.25)	34 (23.8)	

	e4e4	0 (0.00)	1 (1.05)	
PHG	Sample Size, n	27	19	NA
	Age of Death (years)	78.0 (71.5-89.0)	88.0 (83.5-90.0)	0.013 [1]
	Sex, n (%)			0.002 [3]
	Male	19 (70.4)	4 (21.0)	
	Female	8 (29.6)	15 (78.9)	
	Ethnicity, n (%)			0.149 [3]
	White	16 (59.3)	17 (89.5)	
	Other	11 (40.7)	2 (10.5)	
	APOE Genotype, n (%)			0.939 [3]
	e2e3	3 (11.1)	2 (10.5)	
	e2e4	0 (0.00)	0 (0.00)	
	e3e3	11 (40.7)	9 (47.4)	
	e3e4	4 (14.8)	4 (21.1)	
	e4e4	0 (0.00)	1 (3.70)	
	Unknown	9 (33.3)	3 (15.8)	
STG	Sample Size, n	23	33	NA
	Age of Death (years)	83.0 (74.5-90.0)	89.0 (84.0-90.0)	0.037 [1]
	Sex, n (%)			0.394 [2]
	Male	9 (39.1)	9 (27.8)	
	Female	14 (60.9)	24 (72.7)	
	Ethnicity, n (%)			0.638 [2]
	White	16 (69.6)	26 (78.8)	
	Other	7 (30.4)	7 (21.2)	
	APOE Genotype, n (%)			1.00 [3]
	e2e3	2 (8.70)	2 (6.06)	
	e2e4	0 (0.00)	0 (0.00)	
	e3e3	6 (26.1)	9 (27.3)	
	e3e4	1 (4.35)	2 (6.06)	
	e4e4	0 (0.00)	1 (3.03)	
	Unknown	14 (60.9)	19 (57.6)	
IFG	Sample Size, n	36	45	NA
	Age of Death (years)	82.5 (72.3-90.0)	87.0 (83.0-90.0)	0.025 [1]
	Sex, n (%)			0.081 [2]
	Male	19 (52.8)	14 (31.1)	
	Female	17 (47.2)	31 (68.9)	
	Ethnicity, n (%)			0.291 [2]
	White	27 (75.0)	39 (86.7)	
	Other	9 (25.0)	6 (13.3)	
	APOE Genotype, n (%)			0.514 [3]
	e2e3	3 (8.33)	2 (4.44)	
	e2e4	0 (0.00)	0 (0.00)	
	e3e3	11 (30.6)	9 (20.0)	
	e3e4	3 (8.33)	2 (4.44)	
	e4e4	0 (0.00)	1 (2.22)	
	Unknown	19 (52.8)	12 (26.7)	
TCX	Sample Size, n	34	34	NA
	Age of Death (years)	85.5 (79.3-88.8)	83.0 (77.25-88.0)	0.597 [1]

	Sex, n (%)			0.131 [2]
	Male	16 (47.1)	9 (26.5)	
	Female	18 (52.9)	25 (73.5)	
	Ethnicity, n (%)			NA
	White	34 (100)	34 (100)	
	APOE Genotype, n (%)			< 0.001 [3]
	e2e3	5 (14.7)	2 (5.88)	
	e2e4	0 (0.00)	0 (0.00)	
	e3e3	27 (79.4)	13 (38.2)	
	e3e4	2 (5.88)	16 (47.1)	
	e4e4	0 (0.00)	3 (8.82)	
	Unknown	0 (0.00)	0 (0.00)	
CER	Sample Size, n	37	38	NA
	Age of Death (years)	82.0 (78.0-87.0)	84.50 (79.3-89.0)	0.330 [1]
	Sex, n (%)			0.028 [2]
	Male	21 (56.8)	11 (29.0)	
	Female	16 (43.2)	27 (71.1)	
	Ethnicity, n (%)			NA
	White	37 (100)	38 (100)	
	APOE Genotype, n (%)			< 0.001 [3]
	e2e3	4 (10.8)	2 (5.26)	
	e2e4	1 (2.70)	0 (0.00)	
	e3e3	29 (78.3)	16 (42.1)	
	e3e4	3 (8.11)	18 (47.4)	
	e4e4	0 (0.00)	2 (5.26)	
	Unknown	0 (0.00)	0 (0.00)	

[1]Mann-Whitney U test, [2] Pearson's chi-squared test, [3] Fisher's exact test

Supplementary Table 5. Summary of data for DGE analysis. This table summarizes the number of samples per brain region used in DGE analysis and downstream predictive gene expression modeling. It also includes absolute LFC and p-value thresholds for identifying differentially expressed genes (DEGs).

Study	Region	Control Sample Size	AD sample Size	Absolute Log2 Fold Change (LFC)	p-value
ROSMAP	DLPFC	64	95	> 0.60	≤ 0.05
MSBB	PHG	27	19	> 0.75	≤ 0.05
	STG	23	33	> 0.80	≤ 0.05
	IFG	36	45	> 0.65	≤ 0.05
Mayo	TCX	34	34	> 1.50	≤ 0.05
	CER	37	38	> 0.90	≤ 0.05

Supplementary Table 6. Metadata categories across datasets. This table outlines the metadata categories from each study that were used to define region-specific control and AD groups using ASSIGN for DGE signature development validation using independent datasets. These categories included Braak stages, amyloid-beta plaque (e.g., CERAD scores and Thal phases), and study-specific diagnostic criteria. Samples meeting all relevant criteria for a given region

formed its own control and AD training sets, which were then used to train a separate ASSIGN model for that region.

Study	Brain Region	Training Filters	Control Criteria	AD Criteria
DGE signature development				
ROSMAP	DLPFC	Braak stages	0	VI
		CERAD scores	No AD	Definite AD
		Cogdx	NCI	AD
MSBB	PHG	Braak stages	≤ I	VI
		CERAD scores	No AD	Definite AD
		CDR	NCD/Questionable	Severe to terminal (CDR≥3)
	IFG	Braak stages	≤ I	≥ IV
		CERAD scores	No AD	Definite AD
		CDR	NCD/Questionable	Severe to terminal (CDR=5)
	STG	Braak stages	≤ I	VI
		CERAD scores	No AD	Definite AD
		CDR	NCD	Severe to terminal (CDR≥4)
Mayo	TCX	Braak stages	≤ I	VI
		Diagnosis	"Control"	"Alzheimer Disease"
		Thal Phases	No Aβ plaques	Pons and cerebellum
	CER	Braak stages	≤ I	VI
		Diagnosis	"Control"	"Alzheimer Disease"
		Thal Phases	No Aβ plaques	Pons and cerebellum
External DGE signature validation (Independent datasets)				
Aging, Dementia and TBI (GSE104687)	PHG	Braak stages	≤ I	≥ V
		CERAD scores	No AD/Possible AD	Definite AD
		DSM IV Clinical Diagnosis	"No Dementia"	"Alzheimer's Disease Type"
	TCX	Braak stages	≤ II	≥ V
		CERAD scores	No AD/Possible AD	Probable AD/Definite AD
		DSM-IV Clinical Diagnosis	"No Dementia"	"Alzheimer's Disease Type"

AD: Alzheimer's Disease; ROSMAP: The Religious Orders Study and Memory and Aging Project; DLPFC: Dorsolateral prefrontal cortex; CERAD: Consortium to Establish a Registry for Alzheimer's Disease; Cogdx: Final consensus cognitive diagnosis; NCI: No cognitive impairment; MSBB: Mount Sinai Brain Bank; PHG: parahippocampal gyrus; IFG: Inferior frontal gyrus; STG: Superior temporal gyrus; CDR: Clinical Dementia Rating; NCD: No cognitive Deficits; TCX: Temporal cortex; Aβ: Amyloid-beta; CER: Cerebellum; DSM-IV: Diagnostic and Statistical Manual of Mental Disorders-Fourth Edition

Supplementary Table 7A. 176 DEGs identified for PHG. The coefficients of each gene contributing to signature predictions are listed. A positive coefficient indicates that the gene is overexpressed in AD patients, while a negative coefficient signifies its downregulation in AD patients compared to control patients.

PHG	
Gene	Coefficients
XIST	2.5651581
NEAT1	2.56408166
FOS	2.35915541
CXCR4	2.30377458
S100A4	1.99546925
CCN1	1.9541989
LINC03082	1.94673374
ZIC4	1.94132618
ZIC1	1.90485475
PLAUR	1.90299423
ITGA10	1.88871835
DUSP1	1.85914866
C5AR1	1.85602343
ITGAX	1.80421774
GEM	1.79077098
KCNE4	1.78662374
COL1A2	1.77263386
FOXJ1	1.75651519
BCYRN1	1.74338025
LINC01736	1.72548841
NFKB2	1.68747784
C10orf105	1.6831796
OLR1	1.67478961
ATF3	1.65520411
BTG2	1.65324324
SERPINE1	1.63435774
TNC	1.61193144
ADAM33	1.59184478
CD44	1.58564099
SLC13A4	1.5797759
TEAD3	1.57925552
HSPB1	1.57709137
ZFP36	1.55909907
AEBP1	1.54968218
MYH11	1.54810628
TRIP10	1.53436171
JUNB	1.53353989
LINC01094	1.5268111
SMTN	1.50707564
MYL9	1.49010431
RGL3	1.48491567

INHBA	1.47894485
SPN	1.4720181
ITGA5	1.47088458
MS4A6A	1.47023093
A4GALT	1.46857739
KANK2	1.46385005
MS4A7	1.44338917
TRIM5	1.44123843
C11orf96	1.43917283
COL27A1	1.43029088
ZFP36L1	1.42763428
CHST6	1.41025415
PPP1R13L	1.39483143
PARVG	1.39268114
SRPX	1.39049667
PIK3R5	1.38928406
TNFRSF11B	1.37894946
CAPS	1.37575623
ITPKB	1.354303
CSF1	1.35375913
SPARC	1.34814847
GLI2	1.3479871
GFAP	1.34674958
COL6A2	1.33503152
ZDHHC11	1.32611497
A2ML1	1.31837528
ARL17A	1.31467867
PTAFR	1.31234144
C4B	1.29994684
TRIM47	1.29841452
NGFR	1.29283625
TGFBI	1.27849629
PRELP	1.2747354
IGF2	1.2719112
WWTR1	1.26721084
LINC01354	1.26330682
SERPINH1	1.26186294
RASL12	1.25341798
SEMA3F	1.2488234
APLNR	1.24596414
GMPR	1.24231928
ICAM1	1.24206668
BAG3	1.2399466
EPHA2	1.23100491
GBP4	1.22704357

MAFF	1.22521933
CIITA	1.21200415
KLF4	1.21007953
AHNAK	1.20607094
IER3	1.20300091
FXYD5	1.19517395
HSPA1A	1.19464264
NPNT	1.19106696
CP	1.18406783
NWD1	1.18210514
NLRC5	1.15691552
COLEC12	1.14923185
ATP2A3	1.14362808
SCIN	1.14227708
ID3	1.13095148
ITGAL	1.12866452
TGIF1	1.12618952
BGN	1.12238066
SPHK1	1.11047959
LATS2	1.10930592
PLEK	1.10481217
MMP14	1.09938981
C4A	1.09929946
SLC47A2	1.0951776
C3AR1	1.09482573
FTCD	1.08776619
CDKN1A	1.07121879
PTPN6	1.06976535
FZD7	1.06917505
COPZ2	1.06869362
SYTL4	1.05081818
CFAP157	1.04116492
PLEKHA4	1.03364158
ALOX15B	1.03301413
APOL3	1.03131083
HLA-DMB	1.02205097
DOK3	1.0162766
SYNDIG1L	1.01507803
EMP3	1.01422313
MYOF	0.99544233
TLR5	0.96225246
STON1	0.95467004
SLC4A11	0.95323636
DSP	0.94631328
GBP1	0.90052684

NUPR1	0.89581156
APOL1	0.89321036
PDLIM4	0.89145094
RAB20	0.885268
MFAP4	0.87446299
VAMP8	0.85378302
GBP2	0.82753659
SERPINA1	0.81249274
EMP1	0.8121656
C1R	0.80408265
CHI3L1	0.7994235
OAS2	0.7911442
ANXA2	0.77765433
CFI	0.73411579
FBLN1	0.72440004
CASP1	0.70114639
GBP3	0.68679951
MARCHF3	0.66727797
CIMAP3	0.63848151
ANXA1	0.61903882
TOGARAM2	0.57203609
PLA1A	0.31529308
PDYN	-0.6268695
CRH	-0.6827758
PCSK1	-0.8364288
FRMPD2B	-1.0541079
LINC02217	-1.1363253
GCNT4	-1.1651372
STAT4	-1.1883883
PART1	-1.2456838
C3orf80	-1.3375566
SOWAHB	-1.354984
VIP	-1.4001068
PPEF1	-1.4249079
TMEM132E-DT	-1.4597804
RGS4	-1.5410855
MCHR2	-1.5747327
LINC01202	-1.6321363
NEUROD6	-1.6735642
PYDC2-AS1	-1.7237535
FREM3	-1.7784803
LNCBRM	-1.8389763
LINC00507	-1.9277443
LINC01007	-2.0449137

HSPB3	-2.3538689

Supplementary Table 7B. 127 DEGs identified for the STG. The coefficients of each gene contributing to signature predictions are listed. A positive coefficient indicates that the gene is overexpressed in AD patients, while a negative coefficient signifies its downregulation in AD patients compared to control patients.

**STG**	
Gene	Coefficients
FOXJ1	1.97655899
HERC2P3	1.89810731
HK3	1.74683644
PLP2	1.70368497
S100A4	1.68504413
GIMAP7	1.6316996
CD74	1.62741325
LINC01094	1.62025658
GPSM3	1.61419375
FXYD5	1.57077287
TYROBP	1.55999306
LINC01736	1.55369425
C3AR1	1.51200136
CD37	1.50299759
GAL3ST4	1.49438534
BIN2	1.47987916
SPN	1.4650755
SCIN	1.45590527
PRELP	1.45301268
CD163	1.43705627
TMT1B	1.42661233
S100A11	1.42065941
LINC03082	1.38608414
MYL9	1.37921926
EMP3	1.37818252
CFI	1.3771397
FCER1G	1.37335889
FCGR2A	1.36695572
TREM2	1.36144511
SIGLEC8	1.35718551
HSPB1	1.3475694
ID3	1.34407634
S100A9	1.33495654
TEAD2	1.32447459
SELPLG	1.32105096

ABI3	1.31117591
CSF3R	1.30052727
SMTN	1.29714
C4A	1.29115481
C3	1.28898927
ITGB2	1.28723215
ITGAL	1.28690686
SASH3	1.28523536
MMP2	1.28403995
NUPR1	1.28348965
CRYAB	1.27083558
MFNG	1.26718993
LAIR1	1.26188081
MYO1F	1.26128144
SERPINE1	1.22799236
OLR1	1.20982034
C1QC	1.20108708
MYH11	1.19918595
ADORA3	1.193499
SERPINA1	1.19312541
HLA-DRA	1.18105726
HLA-DPA1	1.18048307
HLA-DMB	1.17999528
C1QB	1.17103145
LGALS9	1.15614342
MS4A6A	1.1481122
VAMP8	1.14149732
WAS	1.14045159
CD14	1.1332322
C4B	1.12573677
OAS2	1.11858423
PTAFR	1.11692721
HLA-DOA	1.11310648
NGFR	1.10747362
TMEM119	1.10181042
RHBDF2	1.09917862
PLA1A	1.09643564
ANGPT2	1.09121856
TCIRG1	1.08199994
MS4A7	1.07754726
CD84	1.07752462
MT1F	1.07702497
C10orf105	1.07693384
IFITM1	1.07318688
SYK	1.05333352

TLR5	1.04779617
C1QA	1.02896318
DOK3	1.02376642
CSF1R	1.01425558
PARVG	1.00430632
F13A1	0.99981037
CX3CR1	0.9993267
C5AR1	0.9850982
VSIG4	0.97344931
STAB1	0.97084374
S100A10	0.9668426
LILRB1	0.96124706
IER3	0.95522718
NFAM1	0.93999658
GIMAP2	0.9350687
CTSS	0.92561518
ALOX5AP	0.9245869
SLC7A7	0.92423211
LAPTM5	0.92241089
PIK3AP1	0.9129711
CASP1	0.89452576
PTPRC	0.88467533
HLA-DRB1	0.88066282
CD300A	0.84468119
AZGP1	0.83207707
CD44	0.82967509
SERPINH1	0.79269514
CYBB	0.79224445
TGFBI	0.78903051
UCP2	0.7798487
HMOX1	0.74830616
OAS1	0.71093809
SPP1	0.69054977
PLEK	0.65074937
FCGBP	0.63625453
FOS	0.5717386
MT1G	0.54447674
HSPA1A	0.34699134
HSPA1B	-0.1860294
RAB5IF	-0.9961657
FRMPD2B	-1.2682163
CBLN1	-1.2932394
LINC01007	-1.3302417
LINC00507	-1.8925744
CRH	-2.024923

PPEF1	-2.2866625
NEUROD6	-2.3148296

Supplementary Table 7C. 53 DEGs identified for the TCX. The coefficients of each gene contributing to signature predictions are listed. A positive coefficient indicates that the gene is overexpressed in AD patients, while a negative coefficient signifies its downregulation in AD patients compared to control patients.

**TCX**	
Gene	Coefficients
S100A8	3.07628904
S100A4	2.84340243
LINC03082	2.55250862
GEM	2.37651365
LINC01094	2.37460631
TEAD2	2.25290169
DSP	2.17747903
SLC7A2	2.12337111
ECM2	2.09450333
GFAP	2.05025373
APLNR	2.02356009
TGFBI	1.9954687
TNFRSF11B	1.96818953
AEBP1	1.96003407
CFI	1.92484811
CYP1B1	1.85277703
MFAP4	1.82534681
CD44	1.82486295
CCL2	1.8152526
NPNT	1.77929199
PDLIM1	1.73722174
KCNE4	1.72156103
A4GALT	1.72058739
ANXA2	1.71919534
DCN	1.62784728
FOXC1	1.61924885
ANGPT2	1.49280567
IGF2	1.47402076
THSD4	1.45732889
COL1A2	1.44593693
C4B	1.38245468
COL6A2	1.35478994
OLFML2A	1.31861987
SLC26A2	1.24746202

SLC13A4	1.11680438
SLC47A1	1.09003264
IGFBP5	1.05117642
PHLDB2	0.82134229
GAS1	0.76466097
FREM3	-1.3093789
VGF	-1.3944261
LINC01007	-1.4724781
GAD1	-1.6483942
SERTM1	-1.7654422
NEUROD6	-1.9029646
PPEF1	-1.9687602
DLX6-AS1	-1.9824314
SLC32A1	-2.0113568
GAD2	-2.0468926
TAC1	-2.1565133
PCSK1	-2.3311929
SST	-2.436601
CRH	-2.733187

Supplementary Table 7D. 44 DEGs identified for the IFG. The coefficients of each gene contributing to signature predictions are listed. A positive coefficient indicates that the gene is overexpressed in AD patients, while a negative coefficient signifies its downregulation in AD patients compared to control patients.

**IFG**	
Gene	Coefficients
FOXJ1	2.04745801
PACRG-AS3	1.75811862
HSPA1A	1.68101724
CEDORA	1.65861402
SLC5A11	1.54522191
ADAMTS2	1.53961798
AZGP1	1.42301838
C4B	1.39579116
NGFR	1.38503288
TMPRSS5	1.336707
ANGPT2	1.30686514
LINC03082	1.28216676
S100A4	1.27598272
LINC01736	1.21160118
GNRH1	1.18168245
LINC01094	1.16309207
SLC47A2	1.10401753

ZDHHC11	1.07987131
SLC5A3	1.05020192
NEAT1	0.9365916
CAPS	0.9174605
COL27A1	0.86991461
SPN	0.81991868
CFI	0.6995836
ADORA3	0.63535827
CD44	0.59622789
POU3F4	0.44300392
GPR6	0.4427519
APLNR	0.28850455
MTCO1P12	-0.6933937
ABCC12	-0.7097116
CARTPT	-0.7130538
LINC00507	-0.9009251
NEUROD6	-1.0691363
FRMPD2B	-1.1539236
LINC01007	-1.1557515
LINC01202	-1.1887733
PCSK1	-1.2387251
DUSP4	-1.3033148
ADCYAP1	-1.4276539
PPEF1	-1.4942177
CRH	-1.5178691
VGF	-1.5601695
SST	-1.9287685

Supplementary Table 7E. 53 DEGs identified for the DLPFC. The coefficients of each gene contributing to signature predictions are listed. A positive coefficient indicates that the gene is overexpressed in AD patients, while a negative coefficient signifies its downregulation in AD patients compared to control patients.

**DLPFC**	
Gene	Coefficients
CEDORA	2.85739226
ANGPT2	2.59026871
AZGP1	2.38443098
LINC01736	2.13618737
MAFF	2.08977677
GEM	2.07443118
KCNE4	2.05853987
CCL2	2.0356768
S100A4	1.97356093

MT1F	1.96773337
SLC5A11	1.88650575
SPN	1.87259346
SLCO4A1	1.86621745
MT1H	1.85233541
SYTL4	1.83299275
ANLN	1.83289641
HIGD1B	1.77987256
DDIT4L	1.75446034
TMPRSS5	1.71912064
HILPDA	1.7131153
TRIP10	1.69241211
SLC6A12	1.66525516
FCGBP	1.57797423
SMTN	1.56956544
KIF19	1.56084795
GAREM2	1.55945853
APLN	1.54220248
LINC00499	1.53469962
PHYHD1	1.48521032
PRELP	1.47414688
RIPOR3	1.46891446
CPM	1.43831989
IP6K3	1.34369783
PIEZO2	1.28647017
SLC4A11	1.26843687
SPP1	1.0779381
SLC25A48	0.96362867
LINC03082	0.91838961
HERC2P3	0.80725799
CA12	0.68952949
ADAMTS2	0.49990397
GLP2R	-0.7339677
PPEF1	-1.044422
MCHR2	-1.1363875
VGF	-1.727381
FREM3	-1.7749806
LINC01007	-1.8475507
SST	-1.8566036
TMEM132E-DT	-1.8863602
SOWAHB	-2.0551639
CRH	-2.1513586
FRMPD2B	-2.3846828
PCDHGC5	-2.6448717

Supplementary Table 7F. 55 DEGs identified for the CER. The coefficients of each gene contributing to signature predictions are listed. A positive coefficient indicates that the gene is overexpressed in AD patients, while a negative coefficient signifies its downregulation in AD patients compared to control patients.

**CER**	
Gene	Coefficients
KCNE4	2.50468626
H4C8	2.27846478
GLP2R	2.21827966
TNNT2	2.16976577
ALOX15B	2.03466915
RGS1	1.92380009
HSPA1A	1.91962361
GEM	1.8624146
MAFF	1.81193124
PLK5	1.78971909
RANBP3L	1.753869
ANGPT2	1.72388232
SERPINE1	1.66692542
S100A4	1.61564547
SLC5A3	1.61406474
H2BC5	1.57182712
PCDH18	1.55973916
GPR4	1.54741922
PDLIM1	1.47651044
MCM8	1.47138728
GJA4	1.45922829
LINC02607	1.42661007
MT1L	1.3615935
H4C5	1.33441902
HSPA1B	1.31733493
NPTX2	1.31375513
MT1H	1.28571285
CCN1	1.27506018
H1-2	1.2517274
PLA1A	1.21502951
HGF	1.21225977
CP	1.18614705
DUSP1	1.13410445
CCN2	1.12517019
FOS	1.11906945
BIRC3	1.06398719
DSP	1.04354316

S100A8	1.04318635
TM4SF1	0.97791682
CYP1B1	0.87449623
S100A9	0.50947562
PAX8-AS1	0.39697772
ANO3	-0.6340311
CHRNA2	-0.7791647
MEPE	-0.9712986
BCL11B	-0.9893188
KLK7	-1.0563285
EPN3	-1.2892628
P2RY13	-1.2904635
TRABD2A	-1.3638937
TNC	-1.5562268
HBB	-1.8580431
HBA2	-1.9039428
LINC01299	-2.0998101
CX3CR1	-2.6211984

Supplementary Table 8. Summary of the best-fit GLMs. Summary of the best-fit GLMs for each brain region, identifying the predictors most strongly associated with AD status. DGE signature predictions were significant in all regions except TCX. Inclusion of APOE genotype or sex (both region-dependent) further improved predictive accuracy, with APOE e3/e4 status and male sex consistently emerging as strong predictors alongside DGE signatures.

Region	Best Model Fit Predictors	Sample Size (N)	Odds Ratio (OR)	Standard Error (SE)	p-value	95% Confidence Interval (CI)	Leave-one-out-cross-validation (LOOCV)		Pseudo R^2
							Area Under the Curve (AUC)	95% Confidence Interval (CI)	
DLPFC	DGE signature prediction								
	DLPFC 53 DEGs	159	24.7	0.715	7.4×10^{-6} ***	[6.51, 1.10×10^{2}]			
	APOE genotype								
	e2/e3 (Ref)	15	NA	NA	NA	NA			
	e2/e4	3	5.16	1.38	0.235	[0.368, 1.35×10^{2}]			
	e3/e3	102	1.54	0.671	0.518	[0.413, 5.95]			
	e3/e4	38	9.58	0.857	8.35×10^{-3} **	[1.89, 56.5]	0.815	[0.746,0.884]	0.384
	e4/e4	1	1.92×10^{6}	2.40×10^{3}	0.995	[0.00, NA]			
	Age of Death (Years)								
	70-75 (Ref)	5	NA	NA	NA	NA			
	75-80	15	9.87×10^{5}	1.04×10^{3}	0.989	[0.00, 1.17×10^{147}]			
	80-85	23	2.89×10^{6}	1.04×10^{3}	0.989	[0.00, NA]			
	85+	116	1.10×10^{7}	1.04×10^{3}	0.988	[0.00, NA]			
PHG	DGE signature prediction								
	PHG 176 DEGs	34	2.19×10^{4}	4.55	0.0281 *	[60.6, 2.45×10^{10}]	0.995	[0.806,1.00]	0.689

	Sex								
	Female (Ref)	17	NA	NA	NA	NA			
	Male	17	0.098	1.37	0.0884	[4.00×10^{-3},1.21]			
IFG	DGE signature prediction						0.855	[0.720,0.990]	0.465
	IFG 44 DEGs	43	267	1.69	9.15×10^{-4} ***	[15.9, 1.49×10^{4}]			
	Sex								
	Female (Ref)	23	NA	NA	NA	NA			
	Male	20	0.092	1.024	2.01×10^{-2} *	[0.008, 0.565]			
STG	DGE signature prediction						0.729	[0.473,0.985]	0.355
	STG 127 DEGs	23	169	0.732	0.0153 *	[5.88, 3.63×10^{4}]			
CER	DGE signature prediction						0.877	[0.739,0.951]	0.477
	CER 55 DEGs	75	103	1.12	3.58×10^{-5} ***	[14.4, 1.30×10^{3}]			
	APOE genotype								
	e2/e3 (Ref)	6	NA	NA	NA	NA			
	e2/e4	1	0.00	3.96×10^{3}	0.997	[0.00, Inf]			
	e3/e3	45	3.13	1.2	0.341	[0.334, 41.3]			
	e3/e4	21	27.9	1.38	0.0158 *	[2.20,539]			
	e4/e4	2	1.05×10^{7}	2.69×10^{3}	0.995	[0.00, NA]			
	Sex								
	Female (Ref)	43	NA	NA	NA	NA			
	Male	32	0.374	0.702	0.161	[0.087, 1.44]			
TCX	DGE signature prediction						0.936	[0.746,0.884]	0.894
	TCX 53 DEGs	68	5.17×10^{13}	20.4	0.122	[2.30×10^{4}, 1.11×10^{40}]			
	APOE genotype								
	e2/e3 (Ref)	7	NA	NA	NA	NA			
	e2/e4	0	NA	NA	NA	NA			
	e3/e3	40	2.50×10^{-2}	3.39	0.274	[0.00, 4.05]			
	e3/e4	18	4.40×10^{5}	9.20	0.158	[1.00, 1.46×10^{18}]			
	e4/e4	3	1.97×10^{7}	5.01×10^{3}	0.997	[0.00, NA]			

Significance codes: *** $p < 0.001$, ** $p < 0.01$, * $p < 0.05$

Note: N denotes the sample size after excluding samples with missing data. For categorical predictors, dummy coding was applied, with e2/e3 as the reference level (Ref) for APOE genotype and Female as the reference level for Sex. ORs represent the odds of AD status relative to these reference levels.

Abbreviations: DGE: Differential Gene Expression; DEGs: Differentially expressed genes; APOE: Apolipoprotein E; DLPFC: Dorsolateral prefrontal cortex; PHG: parahippocampal gyrus; IFG: Inferior frontal gyrus; STG: Superior temporal gyrus; CER: Cerebellum; TCX: Temporal cortex

Supplementary Table 9. Signature gene overlap. Signature genes showing overlap across regions are represented using a binary system: 1 indicates the presence of the gene in the regional Differential Gene Expression (DGE) signature, while 0 indicates its absence.

Gene	CER	DLPFG	IFG	PHG	STG	TCX
HGF	1	0	0	0	0	0
BIRC3	1	0	0	0	0	0
TNC	1	0	0	1	0	0
CP	1	0	0	1	0	0
EPN3	1	0	0	0	0	0
GLP2R	1	1	0	0	0	0
RGS1	1	0	0	0	0	0
ANGPT2	1	1	1	0	1	1

DSP	1	0	0	1	0	1
NPTX2	1	0	0	0	0	0
SERPINE1	1	0	0	1	1	0
PDLIM1	1	0	0	0	0	1
TNNT2	1	0	0	0	0	0
CCN2	1	0	0	0	0	0
DUSP1	1	0	0	1	0	0
CHRNA2	1	0	0	0	0	0
MCM8	1	0	0	0	0	0
BCL11B	1	0	0	0	0	0
ANO3	1	0	0	0	0	0
CYP1B1	1	0	0	0	0	1
CCN1	1	0	0	1	0	0
S100A8	1	0	0	0	0	1
PLA1A	1	0	0	1	1	0
KCNE4	1	1	0	1	0	1
MEPE	1	0	0	0	0	0
H2BC5	1	0	0	0	0	0
H4C8	1	0	0	0	0	0
S100A9	1	0	0	0	1	0
RANBP3L	1	0	0	0	0	0
GEM	1	1	0	1	0	1
CX3CR1	1	0	0	0	1	0
KLK7	1	0	0	0	0	0
TM4SF1	1	0	0	0	0	0
FOS	1	0	0	1	1	0
GPR4	1	0	0	0	0	0
ALOX15B	1	0	0	1	0	0
P2RY13	1	0	0	0	0	0
MAFF	1	1	0	1	0	0
PLK5	1	0	0	0	0	0
TRABD2A	1	0	0	0	0	0
GJA4	1	0	0	0	0	0
H1-2	1	0	0	0	0	0
HBA2	1	0	0	0	0	0
PCDH18	1	0	0	0	0	0
PAX8-AS1	1	0	0	0	0	0
S100A4	1	1	1	1	1	1
SLC5A3	1	0	1	0	0	0
HSPA1B	1	0	0	0	1	0
HSPA1A	1	0	1	1	1	0
MT1H	1	1	0	0	0	0
LINC02607	1	0	0	0	0	0
HBB	1	0	0	0	0	0
LINC01299	1	0	0	0	0	0
MT1L	1	0	0	0	0	0
H4C5	1	0	0	0	0	0
ANLN	0	1	0	0	0	0
RIPOR3	0	1	0	0	0	0
CA12	0	1	0	0	0	0
PPEF1	0	1	1	1	1	1
ADAMTS2	0	1	1	0	0	0
SLC4A11	0	1	0	1	0	0
SLCO4A1	0	1	0	0	0	0
SYTL4	0	1	0	1	0	0
CCL2	0	1	0	0	0	1

SLC6A12	0	1	0	0	0	0
SPP1	0	1	0	0	1	0
TRIP10	0	1	0	1	0	0
VGF	0	1	1	0	0	1
HIGD1B	0	1	0	0	0	0
HILPDA	0	1	0	0	0	0
CPM	0	1	0	0	0	0
DDIT4L	0	1	0	0	0	0
SLC25A48	0	1	0	0	0	0
CRH	0	1	1	1	1	1
FRMPD2B	0	1	1	1	1	0
MCHR2	0	1	0	1	0	0
PIEZO2	0	1	0	0	0	0
SST	0	1	1	0	0	1
GAREM2	0	1	0	0	0	0
SLC5A11	0	1	1	0	0	0
AZGP1	0	1	1	0	1	0
IP6K3	0	1	0	0	0	0
TMPRSS5	0	1	1	0	0	0
APLN	0	1	0	0	0	0
PHYHD1	0	1	0	0	0	0
HERC2P3	0	1	0	0	1	0
FREM3	0	1	0	1	0	1
SMTN	0	1	0	1	1	0
SOWAHB	0	1	0	1	0	0
PRELP	0	1	0	1	1	0
KIF19	0	1	0	0	0	0
TMEM132E-DT	0	1	0	1	0	0
SPN	0	1	1	1	1	0
MT1F	0	1	0	0	1	0
LINC01736	0	1	1	1	1	0
LINC01007	0	1	1	1	1	1
PCDHGC5	0	1	0	0	0	0
LINC00499	0	1	0	0	0	0
CEDORA	0	1	1	0	0	0
FCGBP	0	1	0	0	1	0
LINC03082	0	1	1	1	1	1
CD44	0	0	1	1	1	1
NGFR	0	0	1	1	1	0
CAPS	0	0	1	1	0	0
DUSP4	0	0	1	0	0	0
FOXJ1	0	0	1	1	1	0
APLNR	0	0	1	1	0	1
ABCC12	0	0	1	0	0	0
ADCYAP1	0	0	1	0	0	0
GPR6	0	0	1	0	0	0
GNRH1	0	0	1	0	0	0
CARTPT	0	0	1	0	0	0
NEUROD6	0	0	1	1	1	1
PCSK1	0	0	1	1	0	1
SLC47A2	0	0	1	1	0	0
ZDHHC11	0	0	1	1	0	0
COL27A1	0	0	1	1	0	0
POU3F4	0	0	1	0	0	0
CFI	0	0	1	1	1	1

C4B	0	0	1	1	1	1
PACRG-AS3	0	0	1	0	0	0
MTCO1P12	0	0	1	0	0	0
NEAT1	0	0	1	1	0	0
LINC01094	0	0	1	1	1	1
LINC00507	0	0	1	1	1	0
LINC01202	0	0	1	1	0	0
ADORA3	0	0	1	0	1	0
SEMA3F	0	0	0	1	0	0
COPZ2	0	0	0	1	0	0
ITGAL	0	0	0	1	1	0
SCIN	0	0	0	1	1	0
TEAD3	0	0	0	1	0	0
PLAUR	0	0	0	1	0	0
WWTR1	0	0	0	1	0	0
GLI2	0	0	0	1	0	0
ATP2A3	0	0	0	1	0	0
NFKB2	0	0	0	1	0	0
FBLN1	0	0	0	1	0	0
FXYD5	0	0	0	1	1	0
ICAM1	0	0	0	1	0	0
APOL1	0	0	0	1	0	0
PDYN	0	0	0	1	0	0
MYL9	0	0	0	1	1	0
SRPX	0	0	0	1	0	0
RASL12	0	0	0	1	0	0
PPP1R13L	0	0	0	1	0	0
PLEKHA4	0	0	0	1	0	0
HSPB1	0	0	0	1	1	0
AEBP1	0	0	0	1	0	1
MS4A6A	0	0	0	1	1	0
OAS2	0	0	0	1	1	0
PTPN6	0	0	0	1	0	0
SPARC	0	0	0	1	0	0
PLEK	0	0	0	1	1	0
RGS4	0	0	0	1	0	0
GBP3	0	0	0	1	0	0
GBP1	0	0	0	1	0	0
ID3	0	0	0	1	1	0
VAMP8	0	0	0	1	1	0
TGFBI	0	0	0	1	1	1
CXCR4	0	0	0	1	0	0
INHBA	0	0	0	1	0	0
CDKN1A	0	0	0	1	0	0
AHNAK	0	0	0	1	0	0
ZFP36	0	0	0	1	0	0
A4GALT	0	0	0	1	0	1
APOL3	0	0	0	1	0	0
GFAP	0	0	0	1	0	1
PDLIM4	0	0	0	1	0	0
TRIM5	0	0	0	1	0	0
TRIM47	0	0	0	1	0	0
CHI3L1	0	0	0	1	0	0
MYH11	0	0	0	1	1	0
EMP1	0	0	0	1	0	0
ANXA1	0	0	0	1	0	0

KLF4	0	0	0	1	0	0
GMPR	0	0	0	1	0	0
IER3	0	0	0	1	1	0
CASP1	0	0	0	1	1	0
MYOF	0	0	0	1	0	0
STAT4	0	0	0	1	0	0
PARVG	0	0	0	1	1	0
RAB20	0	0	0	1	0	0
ITGAX	0	0	0	1	0	0
NLRC5	0	0	0	1	0	0
PIK3R5	0	0	0	1	0	0
COL6A2	0	0	0	1	0	1
EMP3	0	0	0	1	1	0
EPHA2	0	0	0	1	0	0
ITGA10	0	0	0	1	0	0
ITPKB	0	0	0	1	0	0
DOK3	0	0	0	1	1	0
VIP	0	0	0	1	0	0
SERPINH1	0	0	0	1	1	0
ADAM33	0	0	0	1	0	0
LATS2	0	0	0	1	0	0
BAG3	0	0	0	1	0	0
PART1	0	0	0	1	0	0
ZIC1	0	0	0	1	0	0
FZD7	0	0	0	1	0	0
MMP14	0	0	0	1	0	0
COLEC12	0	0	0	1	0	0
BTG2	0	0	0	1	0	0
C1R	0	0	0	1	0	0
FTCD	0	0	0	1	0	0
CFAP157	0	0	0	1	0	0
ITGA5	0	0	0	1	0	0
GBP2	0	0	0	1	0	0
GBP4	0	0	0	1	0	0
ATF3	0	0	0	1	0	0
COL1A2	0	0	0	1	0	1
SLC13A4	0	0	0	1	0	1
TNFRSF11B	0	0	0	1	0	1
MFAP4	0	0	0	1	0	1
A2ML1	0	0	0	1	0	0
MS4A7	0	0	0	1	1	0
IGF2	0	0	0	1	0	1
NPNT	0	0	0	1	0	1
HSPB3	0	0	0	1	0	0
PTAFR	0	0	0	1	1	0
JUNB	0	0	0	1	0	0
C3AR1	0	0	0	1	1	0
OLR1	0	0	0	1	1	0
MARCHF3	0	0	0	1	0	0
CIMAP3	0	0	0	1	0	0
ZIC4	0	0	0	1	0	0
NUPR1	0	0	0	1	1	0
SPHK1	0	0	0	1	0	0
GCNT4	0	0	0	1	0	0
TGIF1	0	0	0	1	0	0
CIITA	0	0	0	1	0	0

C3orf80	0	0	0	1	0	0
BGN	0	0	0	1	0	0
ANXA2	0	0	0	1	0	1
CHST6	0	0	0	1	0	0
SYNDIG1L	0	0	0	1	0	0
CSF1	0	0	0	1	0	0
ZFP36L1	0	0	0	1	0	0
ARL17A	0	0	0	1	0	0
C11orf96	0	0	0	1	0	0
TLR5	0	0	0	1	1	0
NWD1	0	0	0	1	0	0
TOGARAM2	0	0	0	1	0	0
SERPINA1	0	0	0	1	1	0
KANK2	0	0	0	1	0	0
C5AR1	0	0	0	1	1	0
RGL3	0	0	0	1	0	0
C10orf105	0	0	0	1	1	0
PYDC2-AS1	0	0	0	1	0	0
XIST	0	0	0	1	0	0
LINC01354	0	0	0	1	0	0
BCYRN1	0	0	0	1	0	0
HLA-DMB	0	0	0	1	1	0
STON1	0	0	0	1	0	0
C4A	0	0	0	1	1	0
LINC02217	0	0	0	1	0	0
LNCBRM	0	0	0	1	0	0
STAB1	0	0	0	0	1	0
TYROBP	0	0	0	0	1	0
WAS	0	0	0	0	1	0
CD74	0	0	0	0	1	0
CD84	0	0	0	0	1	0
TEAD2	0	0	0	0	1	1
PTPRC	0	0	0	0	1	0
MMP2	0	0	0	0	1	0
OAS1	0	0	0	0	1	0
TREM2	0	0	0	0	1	0
MFNG	0	0	0	0	1	0
HMOX1	0	0	0	0	1	0
RAB5IF	0	0	0	0	1	0
PLP2	0	0	0	0	1	0
CBLN1	0	0	0	0	1	0
CD37	0	0	0	0	1	0
LILRB1	0	0	0	0	1	0
SIGLEC8	0	0	0	0	1	0
GIMAP2	0	0	0	0	1	0
ABI3	0	0	0	0	1	0
CRYAB	0	0	0	0	1	0
TCIRG1	0	0	0	0	1	0
SELPLG	0	0	0	0	1	0
BIN2	0	0	0	0	1	0
CSF3R	0	0	0	0	1	0
SASH3	0	0	0	0	1	0
F13A1	0	0	0	0	1	0
MT1G	0	0	0	0	1	0
C3	0	0	0	0	1	0
RHBDF2	0	0	0	0	1	0

ALOX5AP	0	0	0	0	1	0
MYO1F	0	0	0	0	1	0
FCGR2A	0	0	0	0	1	0
SLC7A7	0	0	0	0	1	0
PIK3AP1	0	0	0	0	1	0
VSIG4	0	0	0	0	1	0
FCER1G	0	0	0	0	1	0
C1QC	0	0	0	0	1	0
ITGB2	0	0	0	0	1	0
HK3	0	0	0	0	1	0
LAPTM5	0	0	0	0	1	0
CTSS	0	0	0	0	1	0
S100A11	0	0	0	0	1	0
SYK	0	0	0	0	1	0
CYBB	0	0	0	0	1	0
LAIR1	0	0	0	0	1	0
CD300A	0	0	0	0	1	0
LGALS9	0	0	0	0	1	0
TMT1B	0	0	0	0	1	0
CD14	0	0	0	0	1	0
C1QB	0	0	0	0	1	0
C1QA	0	0	0	0	1	0
UCP2	0	0	0	0	1	0
CD163	0	0	0	0	1	0
GIMAP7	0	0	0	0	1	0
CSF1R	0	0	0	0	1	0
TMEM119	0	0	0	0	1	0
IFITM1	0	0	0	0	1	0
HLA-DRB1	0	0	0	0	1	0
GAL3ST4	0	0	0	0	1	0
S100A10	0	0	0	0	1	0
HLA-DOA	0	0	0	0	1	0
HLA-DRA	0	0	0	0	1	0
GPSM3	0	0	0	0	1	0
HLA-DPA1	0	0	0	0	1	0
NFAM1	0	0	0	0	1	0
SLC7A2	0	0	0	0	0	1
TAC1	0	0	0	0	0	1
DCN	0	0	0	0	0	1
FOXC1	0	0	0	0	0	1
SLC32A1	0	0	0	0	0	1
ECM2	0	0	0	0	0	1
IGFBP5	0	0	0	0	0	1
GAD1	0	0	0	0	0	1
GAD2	0	0	0	0	0	1
SLC47A1	0	0	0	0	0	1
PHLDB2	0	0	0	0	0	1
SLC26A2	0	0	0	0	0	1
SERTM1	0	0	0	0	0	1
GAS1	0	0	0	0	0	1
OLFML2A	0	0	0	0	0	1
THSD4	0	0	0	0	0	1
DLX6-AS1	0	0	0	0	0	1

Supplementary Table 10. Identification of hub genes across region. Summary of hub genes identified from signature lists using degree, betweenness, and closeness centrality via CytoHubba. Genes identified across all three metrics were considered key regulators and potential therapeutic targets. The mapped column shows the number of DEGs mapped to GeneMANIA or other databases, highlighting genes with known interactions.

Region	Input DEGs	Mapped DEGs	Hub Genes
PHG	176	159	*AEBP1, C1R, EMP3, SPARC, ZFP36L1*
TCX	53	49	*AEBP1, ANGPT2, COL1A2, COL6A2, CYP1B1, DCN, ECM2, GEM, IGFBP5, TGFBI*
STG	127	119	*FCER1G, HLA-DRA, LAPTM5, TYROBP*
IFG	44	33	*ANGPT2, CRH, GPR6, PPEF1, VGF*
DLPFC	53	46	*CA12, GEM, MAFF, MT1H, PIEZO2, PRELP, SMTN, SPP1, VGF*
CER	55	51	*FOS, GEM, S100A4, TM4SF1*

PHG: parahippocampal gyrus; TCX: Temporal cortex; STG: Superior temporal gyrus; IFG: Inferior frontal gyrus; DLPFC: Dorsolateral prefrontal cortex; CER: Cerebellum

Supplementary Table 11A. PHG significant enrichment terms from g:Profiler.
Terms were derived using gene lists split by upregulated and downregulated genes.

Source	Term Name	Term ID	Adjusted p-value
	**Upregulated**		
GO:MF	extracellular matrix structural constituent	GO:0005201	5.44E-10
GO:MF	cell adhesion molecule binding	GO:0050839	6.24E-09
GO:MF	integrin binding	GO:0005178	5.90E-08
GO:MF	protein-containing complex binding	GO:0044877	1.1857E-06
GO:MF	collagen binding	GO:0005518	0.00039775
GO:MF	peptidase regulator activity	GO:0061134	0.00048176
GO:MF	molecular function inhibitor activity	GO:0140678	0.00313877
GO:MF	molecular function regulator activity	GO:0098772	0.00430544
GO:MF	endopeptidase inhibitor activity	GO:0004866	0.01587413
GO:MF	protein binding	GO:0005515	0.01640651
GO:MF	structural molecule activity	GO:0005198	0.01986412
GO:MF	peptidase inhibitor activity	GO:0030414	0.02130749
GO:MF	endopeptidase regulator activity	GO:0061135	0.03422268
GO:MF	signaling receptor binding	GO:0005102	0.03566105
GO:BP	response to stress	GO:0006950	6.45E-16
GO:BP	response to stimulus	GO:0050896	1.17E-14
GO:BP	tissue development	GO:0009888	1.68E-13
GO:BP	cell adhesion	GO:0007155	4.50E-13
GO:BP	inflammatory response	GO:0006954	4.79E-13
GO:BP	defense response	GO:0006952	6.23E-13
GO:BP	response to external stimulus	GO:0009605	1.82E-12
GO:BP	regulation of response to stimulus	GO:0048583	2.13E-12

GO:BP	regulation of multicellular organismal process	GO:0051239	6.61E-12
GO:BP	regulation of multicellular organismal development	GO:2000026	3.27E-11
GO:BP	circulatory system development	GO:0072359	4.55E-11
GO:BP	positive regulation of biological process	GO:0048518	5.63E-10
GO:BP	animal organ development	GO:0048513	7.18E-10
GO:BP	cell surface receptor signaling pathway	GO:0007166	1.07E-09
GO:BP	response to wounding	GO:0009611	1.16E-09
GO:BP	intracellular signal transduction	GO:0035556	2.09E-09
GO:BP	immune system process	GO:0002376	2.21E-09
GO:BP	intracellular signaling cassette	GO:0141124	4.84E-09
GO:BP	regulation of immune system process	GO:0002682	4.96E-09
GO:BP	positive regulation of multicellular organismal process	GO:0051240	5.43E-09
GO:BP	regulation of developmental process	GO:0050793	5.48E-09
GO:BP	apoptotic process	GO:0006915	5.84E-09
GO:BP	regulation of cell adhesion	GO:0030155	5.86E-09
GO:BP	signaling	GO:0023052	5.90E-09
GO:BP	cell communication	GO:0007154	7.39E-09
GO:BP	positive regulation of cellular process	GO:0048522	9.27E-09
GO:BP	regulation of response to external stimulus	GO:0032101	9.72E-09
GO:BP	positive regulation of response to stimulus	GO:0048584	1.33E-08
GO:BP	positive regulation of immune system process	GO:0002684	1.45E-08
GO:BP	tube development	GO:0035295	1.52E-08
GO:BP	programmed cell death	GO:0012501	1.69E-08
GO:BP	cell death	GO:0008219	1.81E-08
GO:BP	wound healing	GO:0042060	2.34E-08
GO:BP	cell population proliferation	GO:0008283	2.66E-08
GO:BP	response to chemical	GO:0042221	2.89E-08
GO:BP	response to biotic stimulus	GO:0009607	5.91E-08
GO:BP	tube morphogenesis	GO:0035239	5.99E-08
GO:BP	epithelium development	GO:0060429	6.08E-08
GO:BP	blood vessel morphogenesis	GO:0048514	8.24E-08
GO:BP	biological process involved in interspecies interaction between organisms	GO:0044419	1.19E-07
GO:BP	multicellular organism development	GO:0007275	1.20E-07
GO:BP	anatomical structure formation involved in morphogenesis	GO:0048646	1.30E-07
GO:BP	anatomical structure development	GO:0048856	1.84E-07
GO:BP	signal transduction	GO:0007165	1.92E-07
GO:BP	angiogenesis	GO:0001525	2.16E-07
GO:BP	blood vessel development	GO:0001568	2.30E-07
GO:BP	cellular response to stimulus	GO:0051716	2.84E-07
GO:BP	response to cytokine	GO:0034097	3.18E-07
GO:BP	positive regulation of metabolic process	GO:0009893	3.70E-07
GO:BP	response to peptide	GO:1901652	4.50E-07
GO:BP	positive regulation of developmental process	GO:0051094	4.84E-07
GO:BP	response to other organism	GO:0051707	5.14E-07
GO:BP	vasculature development	GO:0001944	5.18E-07
GO:BP	response to external biotic stimulus	GO:0043207	5.41E-07
GO:BP	regulation of cell population proliferation	GO:0042127	5.88E-07
GO:BP	cell-cell adhesion	GO:0098609	6.19E-07
GO:BP	anatomical structure morphogenesis	GO:0009653	6.56E-07

GO:BP	cellular response to cytokine stimulus	GO:0071345	9.63E-07
GO:BP	regulation of signaling	GO:0023051	1.0512E-06
GO:BP	system development	GO:0048731	1.3536E-06
GO:BP	positive regulation of macromolecule metabolic process	GO:0010604	1.3536E-06
GO:BP	multicellular organismal process	GO:0032501	1.5872E-06
GO:BP	regulation of biological process	GO:0050789	1.9604E-06
GO:BP	biological regulation	GO:0065007	2.6518E-06
GO:BP	regulation of cell communication	GO:0010646	3.5817E-06
GO:BP	cell-substrate adhesion	GO:0031589	4.4183E-06
GO:BP	positive regulation of macromolecule biosynthetic process	GO:0010557	5.7855E-06
GO:BP	developmental process	GO:0032502	5.971E-06
GO:BP	regulation of signal transduction	GO:0009966	8.3875E-06
GO:BP	cell adhesion mediated by integrin	GO:0033627	8.396E-06
GO:BP	defense response to other organism	GO:0098542	9.0271E-06
GO:BP	regulation of phosphate metabolic process	GO:0019220	9.5379E-06
GO:BP	regulation of phosphorus metabolic process	GO:0051174	9.7395E-06
GO:BP	MAPK cascade	GO:0000165	9.8645E-06
GO:BP	extracellular matrix organization	GO:0030198	1.0882E-05
GO:BP	extracellular structure organization	GO:0043062	1.1338E-05
GO:BP	external encapsulating structure organization	GO:0045229	1.1812E-05
GO:BP	positive regulation of cell migration	GO:0030335	1.3079E-05
GO:BP	regulation of inflammatory response	GO:0050727	1.6142E-05
GO:BP	positive regulation of biosynthetic process	GO:0009891	1.9151E-05
GO:BP	negative regulation of cell adhesion	GO:0007162	2.1405E-05
GO:BP	regulation of angiogenesis	GO:0045765	2.1405E-05
GO:BP	regulation of cytokine production	GO:0001817	2.3662E-05
GO:BP	regulation of cell differentiation	GO:0045595	2.4289E-05
GO:BP	cytokine production	GO:0001816	2.7135E-05
GO:BP	regulation of vasculature development	GO:1901342	2.7645E-05
GO:BP	positive regulation of cell motility	GO:2000147	2.8669E-05
GO:BP	positive regulation of angiogenesis	GO:0045766	3.0008E-05
GO:BP	cellular response to tumor necrosis factor	GO:0071356	3.1112E-05
GO:BP	regulation of programmed cell death	GO:0043067	3.1424E-05
GO:BP	positive regulation of vasculature development	GO:1904018	3.6141E-05
GO:BP	positive regulation of signaling	GO:0023056	4.3906E-05
GO:BP	positive regulation of locomotion	GO:0040017	4.3988E-05
GO:BP	regulation of defense response	GO:0031347	4.4337E-05
GO:BP	regulation of cell migration	GO:0030334	4.7429E-05
GO:BP	cellular response to chemical stimulus	GO:0070887	5.6246E-05
GO:BP	cellular response to lipid	GO:0071396	5.7078E-05
GO:BP	cell activation	GO:0001775	6.3752E-05
GO:BP	response to tumor necrosis factor	GO:0034612	9.0154E-05
GO:BP	positive regulation of signal transduction	GO:0009967	9.1523E-05
GO:BP	regulation of molecular function	GO:0065009	0.00010019
GO:BP	regulation of MAPK cascade	GO:0043408	0.00010444
GO:BP	cell-matrix adhesion	GO:0007160	0.00013134
GO:BP	immune response	GO:0006955	0.00013373
GO:BP	positive regulation of cell communication	GO:0010647	0.00014885
GO:BP	regulation of cell motility	GO:2000145	0.00015325
GO:BP	ERK1 and ERK2 cascade	GO:0070371	0.00021847

GO:BP	regulation of apoptotic process	GO:0042981	0.0002201
GO:BP	positive regulation of gene expression	GO:0010628	0.00024581
GO:BP	leukocyte cell-cell adhesion	GO:0007159	0.00025171
GO:BP	apoptotic signaling pathway	GO:0097190	0.00025506
GO:BP	regulation of phosphorylation	GO:0042325	0.00031919
GO:BP	innate immune response	GO:0045087	0.00032056
GO:BP	regulation of anatomical structure morphogenesis	GO:0022603	0.00032562
GO:BP	regulation of intracellular signal transduction	GO:1902531	0.00033266
GO:BP	regulation of locomotion	GO:0040012	0.00033884
GO:BP	regulation of cellular process	GO:0050794	0.00034077
GO:BP	regulation of hemopoiesis	GO:1903706	0.00034182
GO:BP	locomotion	GO:0040011	0.00048328
GO:BP	cell migration	GO:0016477	0.00060409
GO:BP	skin development	GO:0043588	0.00060954
GO:BP	regulation of ERK1 and ERK2 cascade	GO:0070372	0.00062964
GO:BP	odontogenesis	GO:0042476	0.00063198
GO:BP	cellular response to type II interferon	GO:0071346	0.00067481
GO:BP	mononuclear cell migration	GO:0071674	0.00072553
GO:BP	response to bacterium	GO:0009617	0.00081458
GO:BP	regulation of localization	GO:0032879	0.00081464
GO:BP	positive regulation of cell differentiation	GO:0045597	0.0008184
GO:BP	cell differentiation	GO:0030154	0.00082832
GO:BP	cellular developmental process	GO:0048869	0.00083471
GO:BP	animal organ morphogenesis	GO:0009887	0.00087596
GO:BP	epidermis development	GO:0008544	0.00092882
GO:BP	regulation of response to stress	GO:0080134	0.00093826
GO:BP	regulation of protein metabolic process	GO:0051246	0.00102392
GO:BP	chemotaxis	GO:0006935	0.00113002
GO:BP	taxis	GO:0042330	0.00119227
GO:BP	leukocyte activation	GO:0045321	0.00120814
GO:BP	negative regulation of response to stimulus	GO:0048585	0.00121967
GO:BP	defense response to symbiont	GO:0140546	0.00167789
GO:BP	negative regulation of developmental process	GO:0051093	0.00186297
GO:BP	regulation of cell development	GO:0060284	0.00189287
GO:BP	positive regulation of cell adhesion	GO:0045785	0.00195728
GO:BP	response to lipid	GO:0033993	0.00203046
GO:BP	regulation of immune response	GO:0050776	0.00270838
GO:BP	response to protozoan	GO:0001562	0.00280746
GO:BP	positive regulation of response to external stimulus	GO:0032103	0.00282486
GO:BP	positive regulation of cell population proliferation	GO:0008284	0.00304328
GO:BP	response to growth factor	GO:0070848	0.00314749
GO:BP	response to type II interferon	GO:0034341	0.00327848
GO:BP	regulation of apoptotic signaling pathway	GO:2001233	0.00327948
GO:BP	humoral immune response mediated by circulating immunoglobulin	GO:0002455	0.00361845
GO:BP	embryo development	GO:0009790	0.00377831
GO:BP	hemopoiesis	GO:0030097	0.00435736
GO:BP	regulation of leukocyte differentiation	GO:1902105	0.00443584
GO:BP	cell motility	GO:0048870	0.004676
GO:BP	epithelial cell differentiation	GO:0030855	0.00507643

GO:BP	regulation of response to cytokine stimulus	GO:0060759	0.00545327
GO:BP	embryonic organ development	GO:0048568	0.00549551
GO:BP	regulation of metabolic process	GO:0019222	0.0057649
GO:BP	leukocyte migration	GO:0050900	0.00607735
GO:BP	positive regulation of immune response	GO:0050778	0.00657404
GO:BP	skeletal system development	GO:0001501	0.00677562
GO:BP	tissue morphogenesis	GO:0048729	0.00716159
GO:BP	cellular response to oxygen-containing compound	GO:1901701	0.00743167
GO:BP	regulation of catalytic activity	GO:0050790	0.00756674
GO:BP	regulation of leukocyte migration	GO:0002685	0.00764124
GO:BP	muscle structure development	GO:0061061	0.00786197
GO:BP	response to virus	GO:0009615	0.00836306
GO:BP	positive regulation of intracellular signal transduction	GO:1902533	0.0096235
GO:BP	response to molecule of bacterial origin	GO:0002237	0.00991498
GO:BP	negative regulation of cell population proliferation	GO:0008285	0.01004666
GO:BP	cytolysis in another organism	GO:0051715	0.010246
GO:BP	response to oxygen-containing compound	GO:1901700	0.01025782
GO:BP	leukocyte differentiation	GO:0002521	0.01116492
GO:BP	positive regulation of cytokine production	GO:0001819	0.01154113
GO:BP	response to endogenous stimulus	GO:0009719	0.01157156
GO:BP	negative regulation of phosphate metabolic process	GO:0045936	0.01235392
GO:BP	negative regulation of phosphorus metabolic process	GO:0010563	0.01235392
GO:BP	regulation of macromolecule metabolic process	GO:0060255	0.01240296
GO:BP	regulation of transferase activity	GO:0051338	0.01264636
GO:BP	regulation of kinase activity	GO:0043549	0.01289691
GO:BP	morphogenesis of an epithelium	GO:0002009	0.01415944
GO:BP	response to abiotic stimulus	GO:0009628	0.01485497
GO:BP	epidermal cell differentiation	GO:0009913	0.01518837
GO:BP	epithelial cell proliferation	GO:0050673	0.01647685
GO:BP	regulation of body fluid levels	GO:0050878	0.01672422
GO:BP	negative regulation of cellular process	GO:0048523	0.01725961
GO:BP	myeloid cell differentiation	GO:0030099	0.01728977
GO:BP	regulation of cellular component organization	GO:0051128	0.0174527
GO:BP	regulation of plasma membrane repair	GO:1905684	0.01784167
GO:BP	positive regulation of pyroptotic inflammatory response	GO:0140639	0.01784167
GO:BP	positive regulation of leukocyte migration	GO:0002687	0.0182658
GO:BP	negative regulation of signaling	GO:0023057	0.01863777
GO:BP	negative regulation of cell communication	GO:0010648	0.01886125
GO:BP	defense response to virus	GO:0051607	0.01942531
GO:BP	negative regulation of programmed cell death	GO:0043069	0.01944238
GO:BP	notochord development	GO:0030903	0.02287894
GO:BP	negative regulation of response to external stimulus	GO:0032102	0.02288707
GO:BP	regulation of myeloid cell differentiation	GO:0045637	0.02552282
GO:BP	cartilage development	GO:0051216	0.02552282
GO:BP	negative regulation of biological process	GO:0048519	0.02701513
GO:BP	growth	GO:0040007	0.02801023
GO:BP	defense response to bacterium	GO:0042742	0.02817472
GO:BP	regulation of cytokine-mediated signaling pathway	GO:0001959	0.02878814
GO:BP	regulation of cell-substrate adhesion	GO:0010810	0.02959108
GO:BP	cellular response to growth factor stimulus	GO:0071363	0.03009287

GO:BP	regulation of lymphocyte differentiation	GO:0045619	0.03419911
GO:BP	response to mechanical stimulus	GO:0009612	0.03544206
GO:BP	regulation of protein phosphorylation	GO:0001932	0.0358775
GO:BP	regulation of interleukin-8 production	GO:0032677	0.03879872
GO:BP	interleukin-8 production	GO:0032637	0.03879872
GO:BP	biological process involved in symbiotic interaction	GO:0044403	0.03940407
GO:BP	negative regulation of molecular function	GO:0044092	0.0407206
GO:BP	cytolysis	GO:0019835	0.04239695
GO:BP	negative regulation of apoptotic process	GO:0043066	0.04352549
GO:BP	regulation of cell activation	GO:0050865	0.0435538
GO:BP	muscle contraction	GO:0006936	0.04464083
GO:BP	regulation of gene expression	GO:0010468	0.04592933
GO:BP	activation of immune response	GO:0002253	0.04674632
GO:BP	negative regulation of signal transduction	GO:0009968	0.04732196
GO:BP	formation of primary germ layer	GO:0001704	0.04793039
GO:BP	blood coagulation	GO:0007596	0.04846683
GO:BP	keratinocyte differentiation	GO:0030216	0.0496987
GO:CC	vesicle	GO:0031982	8.02E-13
GO:CC	cell periphery	GO:0071944	1.22E-12
GO:CC	extracellular matrix	GO:0031012	1.29E-12
GO:CC	external encapsulating structure	GO:0030312	1.35E-12
GO:CC	collagen-containing extracellular matrix	GO:0062023	6.14E-12
GO:CC	endomembrane system	GO:0012505	9.24E-08
GO:CC	extracellular space	GO:0005615	1.41E-07
GO:CC	vesicle membrane	GO:0012506	2.65E-07
GO:CC	cytoplasmic vesicle	GO:0031410	3.25E-07
GO:CC	intracellular vesicle	GO:0097708	3.50E-07
GO:CC	extracellular region	GO:0005576	6.89E-07
GO:CC	cytoplasmic vesicle membrane	GO:0030659	8.24E-07
GO:CC	plasma membrane	GO:0005886	2.055E-06
GO:CC	bounding membrane of organelle	GO:0098588	3.4299E-06
GO:CC	extracellular exosome	GO:0070062	6.8406E-06
GO:CC	secretory granule membrane	GO:0030667	9.2199E-06
GO:CC	extracellular vesicle	GO:1903561	9.9115E-06
GO:CC	extracellular organelle	GO:0043230	1.0033E-05
GO:CC	extracellular membrane-bounded organelle	GO:0065010	1.0033E-05
GO:CC	membrane	GO:0016020	1.0652E-05
GO:CC	cytoplasm	GO:0005737	2.0139E-05
GO:CC	secretory granule	GO:0030141	0.0001805
GO:CC	secretory vesicle	GO:0099503	0.0002829
GO:CC	endoplasmic reticulum lumen	GO:0005788	0.00040256
GO:CC	anchoring junction	GO:0070161	0.0007983
GO:CC	protein complex involved in cell adhesion	GO:0098636	0.00088989
GO:CC	basement membrane	GO:0005604	0.00097723
GO:CC	focal adhesion	GO:0005925	0.00176767
GO:CC	cell-substrate junction	GO:0030055	0.00227019
GO:CC	integrin complex	GO:0008305	0.02034992
GO:CC	specific granule	GO:0042581	0.03088299
GO:CC	tertiary granule	GO:0070820	0.03753862
GO:CC	cell surface	GO:0009986	0.03844877

KEGG	Complement and coagulation cascades	KEGG:04610	1.5688E-06
KEGG	Staphylococcus aureus infection	KEGG:05150	1.5688E-06
KEGG	ECM-receptor interaction	KEGG:04512	0.00333364
KEGG	Pertussis	KEGG:05133	0.01203285
REAC	Interferon gamma signaling	REAC:R-HSA-877300	7.57E-07
REAC	Extracellular matrix organization	REAC:R-HSA-1474244	3.1107E-06
REAC	Integrin cell surface interactions	REAC:R-HSA-216083	7.2014E-05
REAC	Cytokine Signaling in Immune system	REAC:R-HSA-1280215	7.9524E-05
REAC	Interferon Signaling	REAC:R-HSA-913531	0.00108589
REAC	Immune System	REAC:R-HSA-168256	0.00293513
REAC	ECM proteoglycans	REAC:R-HSA-3000178	0.00510804
REAC	Hemostasis	REAC:R-HSA-109582	0.0054868
REAC	Neutrophil degranulation	REAC:R-HSA-6798695	0.01009065
REAC	SMAD2/SMAD3:SMAD4 heterotrimer regulates transcription	REAC:R-HSA-2173796	0.01322168
REAC	Regulation of Insulin-like Growth Factor (IGF) transport and uptake by Insulin-like Growth Factor Binding Proteins (IGFBPs)	REAC:R-HSA-381426	0.01637218
REAC	Post-translational protein phosphorylation	REAC:R-HSA-8957275	0.04715443
WP	Complement and coagulation cascades	WP:WP558	2.9563E-05
WP	Dengue 2 interactions with complement and coagulation cascades	WP:WP3896	3.3899E-05
WP	Hippo Merlin signaling dysregulation	WP:WP4541	0.00010271
WP	Burn wound healing	WP:WP5055	0.00260162
WP	Complement system in neuronal development and plasticity	WP:WP5090	0.00303823
WP	Oxidative damage response	WP:WP3941	0.01111783
WP	Focal adhesion PI3K Akt mTOR signaling	WP:WP3932	0.01619865
WP	Pleural mesothelioma	WP:WP5087	0.03885272
WP	Spinal cord injury	WP:WP2431	0.0403831
WP	TGFB Smad signaling	WP:WP5382	0.04274686
TF	Factor: IRF-7; motif: NNGAAAGTGAAANTR	TF:M11678	0.00313758
TF	Factor: IRF-1; motif: NNRAAANNGAAASN	TF:M07045	0.00734568
TF	Factor: IRF-1; motif: STTTCACTTTCNNT	TF:M04788	0.00769451
TF	Factor: SRF; motif: TTNCCWTWTWTGGNCWNN	TF:M10076	0.01900045
TF	Factor: GKLF; motif: NNRRGRRNGNSNNN	TF:M07040	0.02857584
TF	Factor: IRF7; motif: NCGAAARYGAAANT	TF:M04018	0.03727198
HPA	Skin 2; endothelial cells[≥Low]	HPA:0470201	0.00173333
HPA	Skin 1; extracellular matrix[≥Medium]	HPA:0461432	0.01070979
HPA	Skin 2; extracellular matrix[≥Medium]	HPA:0471432	0.0144116
HPA	Skin 2; langerhans cells[High]	HPA:0471463	0.01517933
HPA	Skin 1; langerhans cells[High]	HPA:0461463	0.01771354
HPA	Skin 2; extracellular matrix[High]	HPA:0471433	0.01858339
HPA	Skin 2; langerhans cells[≥Medium]	HPA:0471462	0.02012446
HPA	Skin 2; fibrohistiocytic cells[High]	HPA:0471443	0.02514879
HPA	Skin 1; fibrohistiocytic cells[High]	HPA:0461443	0.02837214
HPA	Skin 1; extracellular matrix[≥Low]	HPA:0461431	0.03162301
HPA	Skin 2; endothelial cells[≥Medium]	HPA:0470202	0.04612073
HPA	Skin 2; langerhans cells≥Low]	HPA:0471461	0.04783353
HP	Poor wound healing	HP:0001058	0.00910725
**Downregulated**			

GO:MF	peptide hormone receptor binding	GO:0051428	0.0150017
GO:MF	neuropeptide hormone activity	GO:0005184	0.02892697
GO:MF	neuropeptide activity	GO:0160041	0.03098298
GO:MF	hormone receptor binding	GO:0051427	0.04230929
GO:BP	epinephrine secretion	GO:0048242	0.00802634
GO:BP	epinephrine transport	GO:0048241	0.0209947
GO:BP	organic cation transport	GO:0015695	0.04924899
GO:CC	neuronal dense core vesicle	GO:0098992	0.01589978
GO:CC	dense core granule	GO:0031045	0.03134582
KEGG	Neuroactive ligand-receptor interaction	KEGG:04080	0.00231366
REAC	GPCR downstream signalling	REAC:R-HSA-388396	0.01661571
REAC	Signaling by GPCR	REAC:R-HSA-372790	0.02832474

Supplementary Table 11B. STG significant enrichment terms from g:Profiler.
Terms were derived using gene lists split by upregulated and downregulated genes.

Source	Term Name	Term ID	Adjusted_p-value
	**Upregulated**		
GO:MF	immune receptor activity	GO:0140375	2.63E-08
GO:MF	protein-containing complex binding	GO:0044877	1.0882E-06
GO:MF	MHC class II protein complex binding	GO:0023026	1.7071E-06
GO:MF	transmembrane signaling receptor activity	GO:0004888	4.5362E-06
GO:MF	MHC protein complex binding	GO:0023023	1.3491E-05
GO:MF	MHC class II receptor activity	GO:0032395	3.3553E-05
GO:MF	molecular transducer activity	GO:0060089	7.6253E-05
GO:MF	signaling receptor activity	GO:0038023	7.6253E-05
GO:MF	peptide binding	GO:0042277	9.2156E-05
GO:MF	opsonin receptor activity	GO:0001847	0.00089824
GO:MF	protein binding	GO:0005515	0.00144391
GO:MF	peptide antigen binding	GO:0042605	0.00188
GO:MF	amyloid-beta binding	GO:0001540	0.00342333
GO:MF	complement binding	GO:0001848	0.00663578
GO:MF	peptidase regulator activity	GO:0061134	0.02399215
GO:MF	signaling receptor binding	GO:0005102	0.04466252
GO:MF	complement component C5a receptor activity	GO:0004878	0.04981473
GO:MF	2'-5'-oligoadenylate synthetase activity	GO:0001730	0.04981473
GO:MF	ICAM-3 receptor activity	GO:0030369	0.04981473
GO:BP	defense response	GO:0006952	3.67E-30
GO:BP	inflammatory response	GO:0006954	1.18E-26
GO:BP	regulation of immune system process	GO:0002682	3.75E-25
GO:BP	response to stress	GO:0006950	1.46E-24
GO:BP	regulation of immune response	GO:0050776	6.86E-24
GO:BP	immune system process	GO:0002376	1.32E-23
GO:BP	positive regulation of immune system process	GO:0002684	1.52E-23
GO:BP	immune response	GO:0006955	1.18E-20
GO:BP	positive regulation of immune response	GO:0050778	1.35E-20
GO:BP	positive regulation of cytokine production	GO:0001819	1.48E-20
GO:BP	response to external stimulus	GO:0009605	3.29E-20
GO:BP	regulation of cytokine production	GO:0001817	4.14E-19

GO:BP	cytokine production	GO:0001816	5.28E-19
GO:BP	immune effector process	GO:0002252	6.66E-19
GO:BP	positive regulation of multicellular organismal process	GO:0051240	2.89E-18
GO:BP	regulation of multicellular organismal process	GO:0051239	1.21E-17
GO:BP	positive regulation of response to stimulus	GO:0048584	2.01E-17
GO:BP	leukocyte mediated immunity	GO:0002443	2.80E-17
GO:BP	response to stimulus	GO:0050896	3.42E-17
GO:BP	cell activation	GO:0001775	6.55E-17
GO:BP	response to biotic stimulus	GO:0009607	8.65E-17
GO:BP	leukocyte activation	GO:0045321	5.22E-16
GO:BP	response to other organism	GO:0051707	1.95E-15
GO:BP	response to external biotic stimulus	GO:0043207	2.08E-15
GO:BP	cell adhesion	GO:0007155	3.55E-15
GO:BP	biological process involved in interspecies interaction between organisms	GO:0044419	1.00E-14
GO:BP	regulation of immune effector process	GO:0002697	1.71E-14
GO:BP	activation of immune response	GO:0002253	1.71E-14
GO:BP	defense response to other organism	GO:0098542	2.03E-14
GO:BP	regulation of response to stimulus	GO:0048583	2.84E-14
GO:BP	leukocyte cell-cell adhesion	GO:0007159	3.64E-14
GO:BP	regulation of response to external stimulus	GO:0032101	9.23E-14
GO:BP	multicellular organismal process	GO:0032501	1.78E-13
GO:BP	leukocyte migration	GO:0050900	2.57E-13
GO:BP	positive regulation of tumor necrosis factor production	GO:0032760	4.71E-13
GO:BP	tumor necrosis factor production	GO:0032640	7.18E-13
GO:BP	regulation of tumor necrosis factor production	GO:0032680	7.18E-13
GO:BP	positive regulation of tumor necrosis factor superfamily cytokine production	GO:1903557	8.13E-13
GO:BP	regulation of tumor necrosis factor superfamily cytokine production	GO:1903555	1.16E-12
GO:BP	tumor necrosis factor superfamily cytokine production	GO:0071706	1.16E-12
GO:BP	regulation of leukocyte mediated immunity	GO:0002703	2.33E-12
GO:BP	positive regulation of gene expression	GO:0010628	2.53E-12
GO:BP	innate immune response	GO:0045087	3.42E-12
GO:BP	positive regulation of response to external stimulus	GO:0032103	4.73E-12
GO:BP	chemotaxis	GO:0006935	5.78E-12
GO:BP	taxis	GO:0042330	6.31E-12
GO:BP	leukocyte activation involved in immune response	GO:0002366	6.72E-12
GO:BP	cell activation involved in immune response	GO:0002263	8.45E-12
GO:BP	positive regulation of biological process	GO:0048518	1.89E-11
GO:BP	myeloid leukocyte activation	GO:0002274	1.90E-11
GO:BP	cell-cell adhesion	GO:0098609	2.17E-11
GO:BP	adaptive immune response	GO:0002250	2.71E-11
GO:BP	regulation of multicellular organismal development	GO:2000026	3.51E-11
GO:BP	defense response to symbiont	GO:0140546	4.22E-11
GO:BP	T cell activation	GO:0042110	5.30E-11
GO:BP	antigen processing and presentation of peptide antigen via MHC class II	GO:0002495	9.53E-11
GO:BP	immune response-regulating signaling pathway	GO:0002764	1.12E-10
GO:BP	lymphocyte activation	GO:0046649	1.26E-10

GO:BP	lymphocyte mediated immunity	GO:0002449	1.97E-10
GO:BP	cell surface receptor signaling pathway	GO:0007166	3.03E-10
GO:BP	locomotion	GO:0040011	3.11E-10
GO:BP	antigen processing and presentation of peptide or polysaccharide antigen via MHC class II	GO:0002504	3.14E-10
GO:BP	response to chemical	GO:0042221	6.53E-10
GO:BP	regulation of defense response	GO:0031347	8.65E-10
GO:BP	regulation of myeloid leukocyte mediated immunity	GO:0002886	9.16E-10
GO:BP	positive regulation of immune effector process	GO:0002699	1.05E-09
GO:BP	regulation of leukocyte cell-cell adhesion	GO:1903037	1.94E-09
GO:BP	immune response-activating signaling pathway	GO:0002757	1.99E-09
GO:BP	antigen processing and presentation of exogenous peptide antigen via MHC class II	GO:0019886	2.20E-09
GO:BP	adaptive immune response based on somatic recombination of immune receptors built from immunoglobulin superfamily domains	GO:0002460	4.07E-09
GO:BP	regulation of cell adhesion	GO:0030155	4.38E-09
GO:BP	immune response-regulating cell surface receptor signaling pathway	GO:0002768	4.39E-09
GO:BP	regulation of developmental process	GO:0050793	5.91E-09
GO:BP	mononuclear cell differentiation	GO:1903131	8.25E-09
GO:BP	myeloid cell activation involved in immune response	GO:0002275	9.24E-09
GO:BP	cell migration	GO:0016477	1.08E-08
GO:BP	immune response-activating cell surface receptor signaling pathway	GO:0002429	1.21E-08
GO:BP	regulation of cell-cell adhesion	GO:0022407	1.90E-08
GO:BP	positive regulation of cellular process	GO:0048522	1.96E-08
GO:BP	macrophage activation	GO:0042116	2.15E-08
GO:BP	regulation of cell activation	GO:0050865	2.16E-08
GO:BP	cell chemotaxis	GO:0060326	2.25E-08
GO:BP	humoral immune response mediated by circulating immunoglobulin	GO:0002455	2.32E-08
GO:BP	leukocyte differentiation	GO:0002521	2.43E-08
GO:BP	response to cytokine	GO:0034097	2.69E-08
GO:BP	positive regulation of biosynthetic process	GO:0009891	2.72E-08
GO:BP	antigen processing and presentation of exogenous peptide antigen	GO:0002478	3.32E-08
GO:BP	positive regulation of macromolecule biosynthetic process	GO:0010557	3.35E-08
GO:BP	response to peptide	GO:1901652	3.76E-08
GO:BP	negative regulation of immune system process	GO:0002683	3.89E-08
GO:BP	myeloid leukocyte mediated immunity	GO:0002444	4.70E-08
GO:BP	leukocyte chemotaxis	GO:0030595	4.84E-08
GO:BP	myeloid leukocyte migration	GO:0097529	4.84E-08
GO:BP	hemopoiesis	GO:0030097	5.13E-08
GO:BP	phagocytosis	GO:0006909	5.73E-08
GO:BP	leukocyte proliferation	GO:0070661	9.21E-08
GO:BP	immunoglobulin mediated immune response	GO:0016064	1.29E-07
GO:BP	regulation of response to stress	GO:0080134	1.31E-07
GO:BP	cell motility	GO:0048870	1.31E-07
GO:BP	cellular response to cytokine stimulus	GO:0071345	1.36E-07
GO:BP	regulation of hemopoiesis	GO:1903706	1.41E-07

GO:BP	antigen processing and presentation of peptide antigen	GO:0048002	1.43E-07
GO:BP	synapse pruning	GO:0098883	1.54E-07
GO:BP	B cell mediated immunity	GO:0019724	1.54E-07
GO:BP	regulation of transport	GO:0051049	1.84E-07
GO:BP	antigen processing and presentation of exogenous antigen	GO:0019884	2.05E-07
GO:BP	regulation of leukocyte proliferation	GO:0070663	2.23E-07
GO:BP	positive regulation of defense response	GO:0031349	2.51E-07
GO:BP	regulation of leukocyte activation	GO:0002694	2.62E-07
GO:BP	cellular response to chemical stimulus	GO:0070887	3.34E-07
GO:BP	positive regulation of leukocyte cell-cell adhesion	GO:1903039	3.62E-07
GO:BP	regulation of inflammatory response	GO:0050727	3.69E-07
GO:BP	positive regulation of metabolic process	GO:0009893	4.43E-07
GO:BP	regulation of leukocyte migration	GO:0002685	5.25E-07
GO:BP	signaling	GO:0023052	5.27E-07
GO:BP	regulation of lymphocyte proliferation	GO:0050670	6.14E-07
GO:BP	cell communication	GO:0007154	6.32E-07
GO:BP	negative regulation of cell adhesion	GO:0007162	7.57E-07
GO:BP	regulation of localization	GO:0032879	7.60E-07
GO:BP	regulation of mononuclear cell proliferation	GO:0032944	7.94E-07
GO:BP	antigen processing and presentation	GO:0019882	9.29E-07
GO:BP	positive regulation of leukocyte migration	GO:0002687	9.94E-07
GO:BP	positive regulation of macromolecule metabolic process	GO:0010604	1.1011E-06
GO:BP	response to wounding	GO:0009611	1.5966E-06
GO:BP	response to bacterium	GO:0009617	1.9889E-06
GO:BP	negative regulation of cell activation	GO:0050866	2.1343E-06
GO:BP	regulation of lymphocyte activation	GO:0051249	2.2345E-06
GO:BP	regulation of apoptotic signaling pathway	GO:2001233	2.4069E-06
GO:BP	complement activation	GO:0006956	2.6869E-06
GO:BP	regulation of cell population proliferation	GO:0042127	2.7228E-06
GO:BP	regulation of T cell activation	GO:0050863	3.0871E-06
GO:BP	positive regulation of cell-cell adhesion	GO:0022409	3.2682E-06
GO:BP	regulation of leukocyte differentiation	GO:1902105	3.5342E-06
GO:BP	complement activation, classical pathway	GO:0006958	3.663E-06
GO:BP	T cell activation involved in immune response	GO:0002286	4.13E-06
GO:BP	regulation of vesicle-mediated transport	GO:0060627	4.2252E-06
GO:BP	regulation of phagocytosis	GO:0050764	4.3978E-06
GO:BP	regulation of cell motility	GO:2000145	4.4483E-06
GO:BP	lymphocyte activation involved in immune response	GO:0002285	4.6408E-06
GO:BP	anatomical structure development	GO:0048856	5.0183E-06
GO:BP	cellular response to stimulus	GO:0051716	5.3937E-06
GO:BP	regulation of secretion	GO:0051046	5.8804E-06
GO:BP	regulation of cell migration	GO:0030334	7.5448E-06
GO:BP	positive regulation of developmental process	GO:0051094	7.9223E-06
GO:BP	peptide antigen assembly with MHC class II protein complex	GO:0002503	7.948E-06
GO:BP	MHC class II protein complex assembly	GO:0002399	7.948E-06
GO:BP	T cell activation via T cell receptor contact with antigen bound to MHC molecule on antigen presenting cell	GO:0002291	7.948E-06
GO:BP	negative regulation of leukocyte activation	GO:0002695	8.5857E-06
GO:BP	positive regulation of chemotaxis	GO:0050921	8.8479E-06

GO:BP	microglial cell activation	GO:0001774	9.7428E-06
GO:BP	regulation of locomotion	GO:0040012	1.0056E-05
GO:BP	cell junction disassembly	GO:0150146	1.0944E-05
GO:BP	cell population proliferation	GO:0008283	1.1067E-05
GO:BP	regulation of leukocyte degranulation	GO:0043300	1.1318E-05
GO:BP	cell development	GO:0048468	1.1879E-05
GO:BP	negative regulation of immune response	GO:0050777	1.3213E-05
GO:BP	vesicle-mediated transport	GO:0016192	1.4516E-05
GO:BP	neuroinflammatory response	GO:0150076	1.4844E-05
GO:BP	leukocyte activation involved in inflammatory response	GO:0002269	1.5124E-05
GO:BP	positive regulation of endocytosis	GO:0045807	1.5768E-05
GO:BP	signal transduction	GO:0007165	1.6249E-05
GO:BP	lymphocyte proliferation	GO:0046651	1.6553E-05
GO:BP	granulocyte migration	GO:0097530	1.7839E-05
GO:BP	anatomical structure formation involved in morphogenesis	GO:0048646	1.8604E-05
GO:BP	biological regulation	GO:0065007	1.8764E-05
GO:BP	cytokine-mediated signaling pathway	GO:0019221	2.163E-05
GO:BP	positive regulation of cell activation	GO:0050867	2.2033E-05
GO:BP	mononuclear cell proliferation	GO:0032943	2.239E-05
GO:BP	regulation of chemotaxis	GO:0050920	2.3182E-05
GO:BP	regulation of interleukin-8 production	GO:0032677	2.4274E-05
GO:BP	interleukin-8 production	GO:0032637	2.4274E-05
GO:BP	regulation of cell development	GO:0060284	2.5871E-05
GO:BP	neutrophil chemotaxis	GO:0030593	2.6681E-05
GO:BP	regulation of biological process	GO:0050789	2.7689E-05
GO:BP	positive regulation of angiogenesis	GO:0045766	3.0446E-05
GO:BP	glial cell activation	GO:0061900	3.3659E-05
GO:BP	positive regulation of vasculature development	GO:1904018	3.6127E-05
GO:BP	regulation of response to biotic stimulus	GO:0002831	3.8668E-05
GO:BP	granulocyte chemotaxis	GO:0071621	4.5716E-05
GO:BP	multicellular organism development	GO:0007275	4.6492E-05
GO:BP	negative regulation of cell population proliferation	GO:0008285	4.7049E-05
GO:BP	positive regulation of cell communication	GO:0010647	4.8541E-05
GO:BP	positive regulation of signaling	GO:0023056	4.914E-05
GO:BP	humoral immune response	GO:0006959	5.0375E-05
GO:BP	peptide antigen assembly with MHC protein complex	GO:0002501	5.1821E-05
GO:BP	MHC protein complex assembly	GO:0002396	5.1821E-05
GO:BP	innate immune response-activating signaling pathway	GO:0002758	5.6627E-05
GO:BP	regulation of B cell activation	GO:0050864	5.9663E-05
GO:BP	B cell activation	GO:0042113	6.1173E-05
GO:BP	positive regulation of cell migration	GO:0030335	6.2465E-05
GO:BP	positive regulation of mononuclear cell migration	GO:0071677	6.4541E-05
GO:BP	developmental process	GO:0032502	6.7073E-05
GO:BP	positive regulation of cell adhesion	GO:0045785	6.8667E-05
GO:BP	regulation of T cell proliferation	GO:0042129	6.9358E-05
GO:BP	chemokine production	GO:0032602	7.0105E-05
GO:BP	regulation of chemokine production	GO:0032642	7.0105E-05
GO:BP	positive regulation of leukocyte chemotaxis	GO:0002690	7.0105E-05
GO:BP	negative regulation of multicellular organismal process	GO:0051241	7.0358E-05
GO:BP	positive regulation of interleukin-8 production	GO:0032757	7.6746E-05

GO:BP	amyloid-beta clearance	GO:0097242	7.686E-05
GO:BP	positive regulation of apoptotic cell clearance	GO:2000427	8.722E-05
GO:BP	neutrophil activation involved in immune response	GO:0002283	9.2994E-05
GO:BP	positive regulation of leukocyte activation	GO:0002696	0.00010236
GO:BP	response to lipid	GO:0033993	0.00012071
GO:BP	positive regulation of cell motility	GO:2000147	0.00012237
GO:BP	activation of innate immune response	GO:0002218	0.00012371
GO:BP	positive regulation of leukocyte differentiation	GO:1902107	0.00012712
GO:BP	positive regulation of hemopoiesis	GO:1903708	0.00012712
GO:BP	negative regulation of leukocyte mediated immunity	GO:0002704	0.00013046
GO:BP	regulation of lymphocyte mediated immunity	GO:0002706	0.00013345
GO:BP	lymphocyte differentiation	GO:0030098	0.00014009
GO:BP	cell differentiation	GO:0030154	0.00015297
GO:BP	cellular developmental process	GO:0048869	0.00015409
GO:BP	positive regulation of phagocytosis	GO:0050766	0.00015944
GO:BP	neutrophil activation	GO:0042119	0.00016617
GO:BP	regulation of innate immune response	GO:0045088	0.00016993
GO:BP	positive regulation of T cell activation	GO:0050870	0.00017093
GO:BP	positive regulation of chemokine production	GO:0032722	0.00017585
GO:BP	positive regulation of locomotion	GO:0040017	0.00017666
GO:BP	apoptotic signaling pathway	GO:0097190	0.000189
GO:BP	neutrophil migration	GO:1990266	0.00018989
GO:BP	regulation of inflammatory response to antigenic stimulus	GO:0002861	0.00019159
GO:BP	positive regulation of inflammatory response	GO:0050729	0.00020028
GO:BP	T cell differentiation	GO:0030217	0.00020145
GO:BP	regulation of cell differentiation	GO:0045595	0.00020298
GO:BP	positive regulation of signal transduction	GO:0009967	0.00020813
GO:BP	positive regulation of leukocyte mediated immunity	GO:0002705	0.00021179
GO:BP	negative regulation of response to external stimulus	GO:0032102	0.00022148
GO:BP	regulation of apoptotic cell clearance	GO:2000425	0.00025955
GO:BP	endocytosis	GO:0006897	0.00028765
GO:BP	positive regulation of lymphocyte activation	GO:0051251	0.00030017
GO:BP	angiogenesis	GO:0001525	0.00030219
GO:BP	positive regulation of response to biotic stimulus	GO:0002833	0.00031436
GO:BP	inflammatory response to antigenic stimulus	GO:0002437	0.0003358
GO:BP	T cell proliferation	GO:0042098	0.00034855
GO:BP	blood vessel morphogenesis	GO:0048514	0.00035581
GO:BP	transport	GO:0006810	0.00036188
GO:BP	positive regulation of leukocyte proliferation	GO:0070665	0.0003626
GO:BP	negative regulation of lymphocyte activation	GO:0051250	0.0003626
GO:BP	negative regulation of myeloid leukocyte mediated immunity	GO:0002887	0.00040623
GO:BP	programmed cell death	GO:0012501	0.00043529
GO:BP	regulation of secretion by cell	GO:1903530	0.00043709
GO:BP	regulation of lymphocyte differentiation	GO:0045619	0.00044983
GO:BP	cell death	GO:0008219	0.0004542
GO:BP	positive regulation of macrophage migration	GO:1905523	0.00047031
GO:BP	granulocyte activation	GO:0036230	0.0004745
GO:BP	regulation of leukocyte chemotaxis	GO:0002688	0.00051577
GO:BP	leukocyte degranulation	GO:0043299	0.00055726

GO:BP	mononuclear cell migration	GO:0071674	0.00057608
GO:BP	negative regulation of immune effector process	GO:0002698	0.00058333
GO:BP	response to molecule of bacterial origin	GO:0002237	0.00067443
GO:BP	negative regulation of inflammatory response to antigenic stimulus	GO:0002862	0.00068609
GO:BP	negative regulation of cytokine production	GO:0001818	0.00069152
GO:BP	negative regulation of lymphocyte proliferation	GO:0050672	0.00070758
GO:BP	positive regulation of transport	GO:0051050	0.00070978
GO:BP	regulation of angiogenesis	GO:0045765	0.00071538
GO:BP	negative regulation of T cell activation	GO:0050868	0.00078629
GO:BP	regulation of endocytosis	GO:0030100	0.00081827
GO:BP	negative regulation of mononuclear cell proliferation	GO:0032945	0.00082564
GO:BP	production of molecular mediator involved in inflammatory response	GO:0002532	0.00082564
GO:BP	regulation of vasculature development	GO:1901342	0.00087446
GO:BP	coagulation	GO:0050817	0.00088992
GO:BP	innate immune response activating cell surface receptor signaling pathway	GO:0002220	0.00089062
GO:BP	myeloid leukocyte differentiation	GO:0002573	0.00096079
GO:BP	regulation of mast cell degranulation	GO:0043304	0.0009735
GO:BP	complement-mediated synapse pruning	GO:0150062	0.00099139
GO:BP	myeloid cell differentiation	GO:0030099	0.00100329
GO:BP	positive regulation of innate immune response	GO:0045089	0.00110429
GO:BP	regulation of T cell differentiation	GO:0045580	0.00120223
GO:BP	positive regulation of monocyte chemotactic protein-1 production	GO:0071639	0.00121738
GO:BP	interleukin-10 production	GO:0032613	0.00126966
GO:BP	regulation of interleukin-10 production	GO:0032653	0.00126966
GO:BP	regulation of programmed cell death	GO:0043067	0.00129928
GO:BP	regulation of interleukin-4 production	GO:0032673	0.00134854
GO:BP	interleukin-4 production	GO:0032633	0.00134854
GO:BP	negative regulation of leukocyte proliferation	GO:0070664	0.00137764
GO:BP	macrophage migration	GO:1905517	0.00140222
GO:BP	alpha-beta T cell activation	GO:0046631	0.00143249
GO:BP	negative regulation of leukocyte cell-cell adhesion	GO:1903038	0.00145403
GO:BP	positive regulation of lymphocyte proliferation	GO:0050671	0.00153356
GO:BP	regulation of mononuclear cell migration	GO:0071675	0.00153356
GO:BP	secretion	GO:0046903	0.00156625
GO:BP	negative regulation of defense response	GO:0031348	0.00161093
GO:BP	negative regulation of cell-cell adhesion	GO:0022408	0.00162935
GO:BP	positive regulation of mononuclear cell proliferation	GO:0032946	0.00179484
GO:BP	positive regulation of T cell proliferation	GO:0042102	0.00193842
GO:BP	regulation of signaling	GO:0023051	0.0020607
GO:BP	positive regulation of myeloid cell differentiation	GO:0045639	0.00207087
GO:BP	positive regulation of intracellular signal transduction	GO:1902533	0.00209167
GO:BP	regulation of adaptive immune response based on somatic recombination of immune receptors built from immunoglobulin superfamily domains	GO:0002822	0.00209423
GO:BP	regulation of cell communication	GO:0010646	0.00218609
GO:BP	response to oxygen-containing compound	GO:1901700	0.00227041
GO:BP	blood vessel development	GO:0001568	0.00233886

GO:BP	interleukin-6 production	GO:0032635	0.00243283
GO:BP	regulation of interleukin-6 production	GO:0032675	0.00243283
GO:BP	cellular response to cadmium ion	GO:0071276	0.00243587
GO:BP	defense response to bacterium	GO:0042742	0.00244222
GO:BP	microglial cell activation involved in immune response	GO:0002282	0.00246902
GO:BP	pattern recognition receptor signaling pathway	GO:0002221	0.00253043
GO:BP	negative regulation of response to stimulus	GO:0048585	0.00255906
GO:BP	regulation of anatomical structure morphogenesis	GO:0022603	0.0026094
GO:BP	apoptotic process	GO:0006915	0.00269786
GO:BP	regulation of cell killing	GO:0031341	0.00303526
GO:BP	acute inflammatory response	GO:0002526	0.00322769
GO:BP	regulation of myeloid cell differentiation	GO:0045637	0.00324863
GO:BP	anatomical structure morphogenesis	GO:0009653	0.00336149
GO:BP	secretion by cell	GO:0032940	0.00377727
GO:BP	regulation of adaptive immune response	GO:0002819	0.00378694
GO:BP	vasculature development	GO:0001944	0.00393429
GO:BP	regulation of phosphate metabolic process	GO:0019220	0.00402011
GO:BP	regulation of phosphorus metabolic process	GO:0051174	0.00407967
GO:BP	regulation of molecular function	GO:0065009	0.0042763
GO:BP	positive regulation of alpha-beta T cell activation	GO:0046635	0.00442755
GO:BP	regulation of cytokine-mediated signaling pathway	GO:0001959	0.00450186
GO:BP	macrophage activation involved in immune response	GO:0002281	0.00461968
GO:BP	negative regulation of mast cell degranulation	GO:0043305	0.00491921
GO:BP	wound healing	GO:0042060	0.00508091
GO:BP	cellular extravasation	GO:0045123	0.00518098
GO:BP	tube morphogenesis	GO:0035239	0.00556637
GO:BP	positive regulation of cell differentiation	GO:0045597	0.00556637
GO:BP	positive regulation of myeloid leukocyte mediated immunity	GO:0002888	0.00575136
GO:BP	regulation of monocyte chemotactic protein-1 production	GO:0071637	0.00575136
GO:BP	monocyte chemotactic protein-1 production	GO:0071605	0.00575136
GO:BP	regulation of antigen processing and presentation	GO:0002577	0.00575136
GO:BP	cell killing	GO:0001906	0.00588813
GO:BP	positive regulation of T cell differentiation	GO:0045582	0.00610958
GO:BP	regulation of regulated secretory pathway	GO:1903305	0.00610958
GO:BP	type II interferon production	GO:0032609	0.00610958
GO:BP	blood coagulation	GO:0007596	0.00632137
GO:BP	regulation of alpha-beta T cell activation	GO:0046634	0.00645377
GO:BP	regulated exocytosis	GO:0045055	0.00654808
GO:BP	regulation of macrophage migration	GO:1905521	0.00660398
GO:BP	cellular response to lipid	GO:0071396	0.00690761
GO:BP	regulation of response to cytokine stimulus	GO:0060759	0.00727388
GO:BP	regulation of humoral immune response	GO:0002920	0.00737861
GO:BP	regulation of intrinsic apoptotic signaling pathway	GO:2001242	0.00758543
GO:BP	hemostasis	GO:0007599	0.00806072
GO:BP	regulation of body fluid levels	GO:0050878	0.00809005
GO:BP	intracellular signal transduction	GO:0035556	0.00837268
GO:BP	positive regulation of CD4-positive, CD25-positive, alpha-beta regulatory T cell differentiation	GO:0032831	0.00857577
GO:BP	regulation of exocytosis	GO:0017157	0.00894735

GO:BP	negative regulation of cell motility	GO:2000146	0.00904257
GO:BP	leukocyte adhesion to vascular endothelial cell	GO:0061756	0.00914081
GO:BP	establishment of localization	GO:0051234	0.01029564
GO:BP	regulation of apoptotic process	GO:0042981	0.01043802
GO:BP	import into cell	GO:0098657	0.01049775
GO:BP	response to amyloid-beta	GO:1904645	0.01121697
GO:BP	apoptotic cell clearance	GO:0043277	0.01121697
GO:BP	positive regulation of ERK1 and ERK2 cascade	GO:0070374	0.01137577
GO:BP	export from cell	GO:0140352	0.01143402
GO:BP	regulation of catalytic activity	GO:0050790	0.01143402
GO:BP	circulatory system development	GO:0072359	0.01273714
GO:BP	cellular defense response	GO:0006968	0.01364532
GO:BP	regulation of CD4-positive, CD25-positive, alpha-beta regulatory T cell differentiation	GO:0032829	0.0136689
GO:BP	regulation of neutrophil degranulation	GO:0043313	0.0136689
GO:BP	positive regulation of lymphocyte differentiation	GO:0045621	0.01389695
GO:BP	negative regulation of locomotion	GO:0040013	0.01488049
GO:BP	positive regulation of cell population proliferation	GO:0008284	0.0164203
GO:BP	phagocytosis, engulfment	GO:0006911	0.01646672
GO:BP	regulation of leukocyte mediated cytotoxicity	GO:0001910	0.01680994
GO:BP	interleukin-1 beta production	GO:0032611	0.01680994
GO:BP	regulation of interleukin-1 beta production	GO:0032651	0.01680994
GO:BP	positive regulation of interleukin-4 production	GO:0032753	0.0173225
GO:BP	positive regulation of alpha-beta T cell differentiation	GO:0046638	0.0180383
GO:BP	leukocyte mediated cytotoxicity	GO:0001909	0.01840597
GO:BP	positive regulation of leukocyte degranulation	GO:0043302	0.02025332
GO:BP	response to lipopolysaccharide	GO:0032496	0.02090673
GO:BP	mast cell degranulation	GO:0043303	0.02153162
GO:BP	regulation of ERK1 and ERK2 cascade	GO:0070372	0.02500838
GO:BP	intracellular signaling cassette	GO:0141124	0.02518982
GO:BP	mast cell activation involved in immune response	GO:0002279	0.0255317
GO:BP	positive regulation of cytokine-mediated signaling pathway	GO:0001961	0.0255317
GO:BP	regulation of cellular process	GO:0050794	0.02603973
GO:BP	regulation of macrophage activation	GO:0043030	0.02773734
GO:BP	response to cadmium ion	GO:0046686	0.02773734
GO:BP	mast cell mediated immunity	GO:0002448	0.02773734
GO:BP	tube development	GO:0035295	0.02801566
GO:BP	cellular response to low-density lipoprotein particle stimulus	GO:0071404	0.03124635
GO:BP	regulation of T cell mediated immunity	GO:0002709	0.03194179
GO:BP	positive regulation of myeloid leukocyte differentiation	GO:0002763	0.03259244
GO:BP	exocytosis	GO:0006887	0.03508903
GO:BP	plasma membrane invagination	GO:0099024	0.03525547
GO:BP	system development	GO:0048731	0.0355438
GO:BP	response to lipoprotein particle	GO:0055094	0.03573121
GO:BP	regulation of canonical NF-kappaB signal transduction	GO:0043122	0.03609713
GO:BP	regulation of signal transduction	GO:0009966	0.03759079
GO:BP	CD4-positive, CD25-positive, alpha-beta regulatory T cell differentiation	GO:0002361	0.03981559
GO:BP	cellular component disassembly	GO:0022411	0.0399653

GO:BP	negative regulation of B cell activation	GO:0050869	0.04067153
GO:BP	cell surface toll-like receptor signaling pathway	GO:0140895	0.04108762
GO:BP	negative regulation of cell migration	GO:0030336	0.0413558
GO:BP	interleukin-1 production	GO:0032612	0.04395792
GO:BP	regulation of interleukin-1 production	GO:0032652	0.04395792
GO:BP	regulation of B cell proliferation	GO:0030888	0.04427132
GO:BP	positive regulation of response to cytokine stimulus	GO:0060760	0.04427132
GO:BP	negative regulation of inflammatory response	GO:0050728	0.04654537
GO:BP	antigen processing and presentation of endogenous peptide antigen via MHC class II	GO:0002491	0.04843268
GO:BP	myeloid dendritic cell antigen processing and presentation	GO:0002469	0.04843268
GO:BP	positive regulation of MAPK cascade	GO:0043410	0.04897137
GO:BP	ERK1 and ERK2 cascade	GO:0070371	0.04980303
GO:CC	cell periphery	GO:0071944	2.16E-20
GO:CC	plasma membrane	GO:0005886	6.78E-15
GO:CC	vesicle	GO:0031982	1.95E-14
GO:CC	secretory granule	GO:0030141	3.94E-14
GO:CC	secretory granule membrane	GO:0030667	6.25E-14
GO:CC	membrane	GO:0016020	2.47E-12
GO:CC	cytoplasmic vesicle	GO:0031410	3.64E-12
GO:CC	intracellular vesicle	GO:0097708	3.97E-12
GO:CC	secretory vesicle	GO:0099503	6.59E-12
GO:CC	vesicle membrane	GO:0012506	4.49E-11
GO:CC	cytoplasmic vesicle membrane	GO:0030659	1.93E-10
GO:CC	extracellular region	GO:0005576	4.69E-10
GO:CC	endomembrane system	GO:0012505	2.38E-09
GO:CC	MHC class II protein complex	GO:0042613	5.92E-08
GO:CC	cell surface	GO:0009986	6.05E-08
GO:CC	collagen-containing extracellular matrix	GO:0062023	6.21E-08
GO:CC	tertiary granule	GO:0070820	2.41E-07
GO:CC	bounding membrane of organelle	GO:0098588	2.94E-07
GO:CC	extracellular space	GO:0005615	4.91E-07
GO:CC	MHC protein complex	GO:0042611	6.25E-07
GO:CC	extracellular exosome	GO:0070062	9.22E-07
GO:CC	tertiary granule membrane	GO:0070821	1.0578E-06
GO:CC	extracellular matrix	GO:0031012	1.1737E-06
GO:CC	external encapsulating structure	GO:0030312	1.2054E-06
GO:CC	extracellular vesicle	GO:1903561	1.314E-06
GO:CC	extracellular membrane-bounded organelle	GO:0065010	1.3294E-06
GO:CC	extracellular organelle	GO:0043230	1.3294E-06
GO:CC	external side of plasma membrane	GO:0009897	7.0595E-06
GO:CC	endocytic vesicle	GO:0030139	9.5476E-06
GO:CC	ficolin-1-rich granule	GO:0101002	1.1789E-05
GO:CC	endocytic vesicle membrane	GO:0030666	3.2614E-05
GO:CC	organelle membrane	GO:0031090	4.2684E-05
GO:CC	cytoplasm	GO:0005737	6.4971E-05
GO:CC	lysosome	GO:0005764	0.00012031
GO:CC	lytic vacuole	GO:0000323	0.00012031
GO:CC	specific granule membrane	GO:0035579	0.00012761
GO:CC	lytic vacuole membrane	GO:0098852	0.00017109

GO:CC	lysosomal membrane	GO:0005765	0.00017109
GO:CC	side of membrane	GO:0098552	0.0004011
GO:CC	vacuolar membrane	GO:0005774	0.00051718
GO:CC	vacuole	GO:0005773	0.00061408
GO:CC	extrinsic component of presynaptic membrane	GO:0098888	0.00344845
GO:CC	ficolin-1-rich granule membrane	GO:0101003	0.00448289
GO:CC	specific granule	GO:0042581	0.00547778
GO:CC	lumenal side of endoplasmic reticulum membrane	GO:0098553	0.00589934
GO:CC	membrane raft	GO:0045121	0.00674529
GO:CC	membrane microdomain	GO:0098857	0.00711381
GO:CC	macrophage migration inhibitory factor receptor complex	GO:0035692	0.00837639
GO:CC	integrin alphaL-beta2 complex	GO:0034687	0.00837639
GO:CC	complement component C1q complex	GO:0062167	0.00837639
GO:CC	complement component C1 complex	GO:0005602	0.00837639
GO:CC	clathrin-coated endocytic vesicle membrane	GO:0030669	0.01093934
GO:CC	late endosome	GO:0005770	0.01305533
GO:CC	extrinsic component of postsynaptic membrane	GO:0098890	0.01466749
GO:CC	lumenal side of membrane	GO:0098576	0.01825542
GO:CC	autolysosome membrane	GO:0120281	0.02504522
GO:CC	blood microparticle	GO:0072562	0.02593035
GO:CC	clathrin-coated endocytic vesicle	GO:0045334	0.03029505
GO:CC	COPII-coated ER to Golgi transport vesicle	GO:0030134	0.03194765
GO:CC	phagocytic vesicle	GO:0045335	0.03531226
KEGG	Staphylococcus aureus infection	KEGG:05150	1.00E-19
KEGG	Complement and coagulation cascades	KEGG:04610	3.16E-12
KEGG	Coronavirus disease - COVID-19	KEGG:05171	2.77E-08
KEGG	Tuberculosis	KEGG:05152	6.15E-08
KEGG	Phagosome	KEGG:04145	7.70E-08
KEGG	Leishmaniasis	KEGG:05140	9.29E-08
KEGG	Pertussis	KEGG:05133	1.60E-07
KEGG	Systemic lupus erythematosus	KEGG:05322	2.65E-07
KEGG	Hematopoietic cell lineage	KEGG:04640	1.0738E-06
KEGG	Rheumatoid arthritis	KEGG:05323	1.0368E-05
KEGG	Asthma	KEGG:05310	1.3075E-05
KEGG	Antigen processing and presentation	KEGG:04612	2.0466E-05
KEGG	Cell adhesion molecules	KEGG:04514	0.0001346
KEGG	Viral myocarditis	KEGG:05416	0.00016353
KEGG	Alcoholic liver disease	KEGG:04936	0.0006016
KEGG	Legionellosis	KEGG:05134	0.00115399
KEGG	Allograft rejection	KEGG:05330	0.00131303
KEGG	Epstein-Barr virus infection	KEGG:05169	0.00135109
KEGG	Graft-versus-host disease	KEGG:05332	0.0020119
KEGG	Inflammatory bowel disease	KEGG:05321	0.00208896
KEGG	Type I diabetes mellitus	KEGG:04940	0.00297066
KEGG	Intestinal immune network for IgA production	KEGG:04672	0.00476056
KEGG	Neutrophil extracellular trap formation	KEGG:04613	0.00565406
KEGG	Autoimmune thyroid disease	KEGG:05320	0.00805681
KEGG	Influenza A	KEGG:05164	0.01473628
KEGG	Th1 and Th2 cell differentiation	KEGG:04658	0.01613704
KEGG	Osteoclast differentiation	KEGG:04380	0.02069517

KEGG	Chagas disease	KEGG:05142	0.03217787
KEGG	Th17 cell differentiation	KEGG:04659	0.0396601
KEGG	Toxoplasmosis	KEGG:05145	0.04611361
REAC	Immune System	REAC:R-HSA-168256	9.89E-17
REAC	Innate Immune System	REAC:R-HSA-168249	2.69E-14
REAC	Neutrophil degranulation	REAC:R-HSA-6798695	9.08E-14
REAC	Adaptive Immune System	REAC:R-HSA-1280218	5.0402E-06
REAC	Cell surface interactions at the vascular wall	REAC:R-HSA-202733	0.00014798
REAC	Regulation of Complement cascade	REAC:R-HSA-977606	0.00058089
REAC	Complement cascade	REAC:R-HSA-166658	0.00118941
REAC	Interferon gamma signaling	REAC:R-HSA-877300	0.00335646
REAC	Cytokine Signaling in Immune system	REAC:R-HSA-1280215	0.00424463
REAC	Immunoregulatory interactions between a Lymphoid and a non-Lymphoid cell	REAC:R-HSA-198933	0.00613866
REAC	Activation of C3 and C5	REAC:R-HSA-174577	0.00734879
REAC	Hemostasis	REAC:R-HSA-109582	0.00886791
REAC	Phosphorylation of CD3 and TCR zeta chains	REAC:R-HSA-202427	0.01308982
REAC	Cell recruitment (pro-inflammatory response)	REAC:R-HSA-9664424	0.01847278
REAC	Purinergic signaling in leishmaniasis infection	REAC:R-HSA-9660826	0.01847278
REAC	Parasitic Infection Pathways	REAC:R-HSA-9824443	0.02208395
REAC	Leishmania infection	REAC:R-HSA-9658195	0.02208395
REAC	MHC class II antigen presentation	REAC:R-HSA-2132295	0.02396322
WP	TYROBP causal network in microglia	WP:WP3945	1.94E-12
WP	Microglia pathogen phagocytosis pathway	WP:WP3937	2.08E-08
WP	Complement and coagulation cascades	WP:WP558	3.06E-08
WP	Dengue 2 interactions with complement and coagulation cascades	WP:WP3896	3.66E-08
WP	Allograft rejection	WP:WP2328	1.30E-07
WP	Complement system in neuronal development and plasticity	WP:WP5090	9.79E-07
WP	Complement system	WP:WP2806	3.9856E-06
WP	Complement activation	WP:WP545	8.7086E-06
WP	Oxidative damage response	WP:WP3941	0.00038891
WP	Cells and molecules involved in local acute inflammatory response	WP:WP4493	0.00329159
WP	Fibrin complement receptor 3 signaling	WP:WP4136	0.01148469
WP	Macrophage markers	WP:WP4146	0.01293718
WP	Extrafollicular and follicular B cell activation by SARS CoV 2	WP:WP5218	0.01437696
WP	Complement mediated inflammation of pulmonary alveolus in COVID 19 hypothetical pathway	WP:WP5148	0.03316692
WP	Ebola virus infection in host	WP:WP4217	0.04389965
TF	Factor: PU.1; motif: NRAAAGAGGAAGTGRNN	TF:M10073	6.3567E-06
TF	Factor: Spi-B; motif: NRAAAGAGGAAGTGARA	TF:M10074	1.3516E-05
TF	Factor: SREBP-1; motif: CACSCCA; match class: 1	TF:M00749_1	4.9346E-05
TF	Factor: PU.1; motif: NRAAAGAGGAAGTGARA	TF:M09659	0.00028305
TF	Factor: PU.1; motif: NNNNYYYACTTCCTCTTTY	TF:M01172	0.00131034
TF	Factor: GATAD2A; motif: CCTKTG; match class: 1	TF:M09726_1	0.00264403
TF	Factor: SPI1; motif: NNNAAAGAGGAAGTGANNNN	TF:M12497	0.00414019
TF	Factor: DREF; motif: CTYYCWCTTCCY	TF:M09725	0.00427437
TF	Factor: c-Ets-1; motif: RCAGGAAGTGNNTNS; match class: 1	TF:M00339_1	0.00482982

TF	Factor: SPI1; motif: NGRGGAAGTN	TF:M02078	0.00707778
TF	Factor: p53; motif: NGRCWTGYCY	TF:M00272	0.00988729
TF	Factor: SPIB; motif: NNTCACTTCCTCTTTN	TF:M12498	0.01083671
TF	Factor: Elf-1; motif: RNWMBAGGAART	TF:M00746	0.01225758
TF	Factor: DREF; motif: CTYYCWCTTCCY; match class: 1	TF:M09725_1	0.01871231
TF	Factor: PEA3; motif: ACWTCCK; match class: 1	TF:M00655_1	0.02322329
TF	Factor: c-Ets-1; motif: RCAGGAAGTGNNTNS	TF:M00339	0.02786481
HPA	Skin 2; fibrohistiocytic cells[≥Medium]	HPA:0471442	2.47E-09
HPA	Skin 2; fibrohistiocytic cells[High]	HPA:0471443	4.41E-09
HPA	Skin 2; fibrohistiocytic cells[≥Low]	HPA:0471441	6.69E-08
HPA	Rectum; mucosal lymphoid cells[≥Medium]	HPA:0401332	1.14E-07
HPA	Skin 1; fibrohistiocytic cells[High]	HPA:0461443	1.60E-07
HPA	Colon; mucosal lymphoid cells[≥Low]	HPA:0131331	1.0943E-06
HPA	Skin 1; fibrohistiocytic cells[≥Low]	HPA:0461441	1.5173E-06
HPA	Skin 1; fibrohistiocytic cells[≥Medium]	HPA:0461442	1.7186E-06
HPA	Rectum; mucosal lymphoid cells[≥Low]	HPA:0401331	3.2768E-06
HPA	Appendix; non-germinal center cells[≥Medium]	HPA:0030442	6.8828E-06
HPA	Skin 2; langerhans cells[High]	HPA:0471463	8.3307E-06
HPA	Colon; mucosal lymphoid cells[≥Medium]	HPA:0131332	1.079E-05
HPA	Skin 2; langerhans cells[≥Low]	HPA:0471461	1.6555E-05
HPA	Appendix; non-germinal center cells[≥Low]	HPA:0030441	2.1934E-05
HPA	Skin 2; lymphocytes[≥Medium]	HPA:0471472	5.5902E-05
HPA	Spleen; cells in red pulp[High]	HPA:0530713	0.00014997
HPA	Skin 1; langerhans cells[≥Low]	HPA:0461461	0.00028018
HPA	Skin 2; langerhans cells[≥Medium]	HPA:0471462	0.0003652
HPA	Skin 1; langerhans cells[High]	HPA:0461463	0.00041972
HPA	Endometrium; macrophages[≥Medium]	HPA:0640412	0.00049098
HPA	Endometrium; macrophages[≥Low]	HPA:0640411	0.00049098
HPA	Skin 1; extracellular matrix[≥Medium]	HPA:0461432	0.000622
HPA	Skin 2; extracellular matrix[≥Medium]	HPA:0471432	0.00089484
HPA	Skin 1; langerhans cells[≥Medium]	HPA:0461462	0.00102843
HPA	Skin 2; lymphocytes[High]	HPA:0471473	0.00106144
HPA	Endometrium	HPA:0640000	0.0015657
HPA	Skin 2; lymphocytes[≥Low]	HPA:0471471	0.00159766
HPA	Skin 1; extracellular matrix[≥Low]	HPA:0461431	0.00234343
HPA	Rectum; mucosal lymphoid cells[High]	HPA:0401333	0.00368249
HPA	Appendix; germinal center cells[≥Medium]	HPA:0030432	0.00407593
HPA	Cerebellum; molecular layer cells - cytoplasm/membrane[High]	HPA:0091003	0.00421273
HPA	Endometrium; macrophages[High]	HPA:0640413	0.0047348
HPA	Skin 2; extracellular matrix[≥Low]	HPA:0471431	0.00485885
HPA	Appendix; germinal center cells[≥Low]	HPA:0030431	0.00559121
HPA	Appendix; non-germinal center cells[High]	HPA:0030443	0.00574701
HPA	Lung; macrophages[High]	HPA:0300413	0.00744193
HPA	Skin 1; lymphocytes[High]	HPA:0461473	0.02279157
HPA	Cerebellum; granular cells - cytoplasm/membrane[High]	HPA:0090973	0.02287754
HPA	Cerebellum; granular cells - cytoplasm/membrane[≥Medium]	HPA:0090972	0.02761715
HPA	Spleen; cells in red pulp[≥Low]	HPA:0530711	0.027857
HPA	Endometrium; nonciliated luminal epithelial cells[≥Low]	HPA:0641521	0.03013909

HPA	Cerebellum; granular cells - cytoplasm/membrane[≥Low]	HPA:0090971	0.03851718
CORUM	C1q complex	CORUM:6418	0.0166065
HP	Reduced circulating complement concentration	HP:0004431	2.71E-08
HP	Abnormality of complement system	HP:0005339	4.64E-08
HP	Autoimmunity	HP:0002960	1.77E-07
HP	Antinuclear antibody positivity	HP:0003493	4.07E-07
HP	Autoimmune antibody positivity	HP:0030057	1.3294E-06
HP	Nephritis	HP:0000123	1.9479E-06
HP	Discoid lupus rash	HP:0007417	2.6257E-05
HP	Glomerulonephritis	HP:0000099	3.2989E-05
HP	Abnormality of humoral immunity	HP:0005368	8.9221E-05
HP	Skin rash	HP:0000988	0.00012318
HP	Unusual infection	HP:0032101	0.00022053
HP	Abnormal leukocyte morphology	HP:0001881	0.00034486
HP	Abnormal cellular immune system morphology	HP:0010987	0.00034486
HP	Inflammatory abnormality of the skin	HP:0011123	0.00037957
HP	Antineutrophil antibody positivity	HP:0003453	0.00037967
HP	Abnormality of skin physiology	HP:0011122	0.00045277
HP	Abnormal immune system morphology	HP:0032251	0.00066749
HP	Malar rash	HP:0025300	0.00078182
HP	Abnormal renal glomerulus morphology	HP:0000095	0.00092712
HP	Abnormal renal corpuscle morphology	HP:0031263	0.00097333
HP	Sinusitis	HP:0000246	0.00142199
HP	Decreased circulating complement C3 concentration	HP:0005421	0.00144875
HP	Abnormal inflammatory response	HP:0012647	0.00151745
HP	Increased inflammatory response	HP:0012649	0.00151745
HP	Recurrent infections	HP:0002719	0.00152563
HP	Abnormality of immune system physiology	HP:0010978	0.00162136
HP	Abnormal oral mucosa morphology	HP:0011830	0.00169687
HP	Oral ulcer	HP:0000155	0.00272385
HP	Vasculitis	HP:0002633	0.00272385
HP	Erosion of oral mucosa	HP:0031446	0.00274208
HP	Unusual CNS infection	HP:0011450	0.00316603
HP	Abnormal pleura morphology	HP:0002103	0.00323116
HP	Abnormal paranasal sinus morphology	HP:0000245	0.00391737
HP	Abnormality of the immune system	HP:0002715	0.00454293
HP	Unusual infection by anatomical site	HP:0032158	0.00511808
HP	Abnormal renal cortex morphology	HP:0011035	0.0051669
HP	Arthritis	HP:0001369	0.00629116
HP	Lupus nephritis	HP:0033726	0.00667554
HP	Anti-Sm antibody positivity	HP:0033040	0.00667554
HP	Recurrent lower respiratory tract infections	HP:0002783	0.00706425
HP	Elevated circulating C-reactive protein concentration	HP:0011227	0.00706808
HP	Recurrent meningitis	HP:0006946	0.00754724
HP	Abnormality of blood and blood-forming tissues	HP:0001871	0.00803197
HP	Acute phase response	HP:0033331	0.00845384
HP	Abnormal circulating C-reactive protein concentration	HP:0032436	0.00894883
HP	Extractable nuclear antigen positivity	HP:0033476	0.00920861
HP	Anti-dsDNA antibody positivity	HP:0020151	0.00920861
HP	Systemic lupus erythematosus	HP:0002725	0.00920861

HP	Hemolytic anemia	HP:0001878	0.00953892
HP	Recurrent bacterial infections	HP:0002718	0.00960934
HP	Hemoptysis	HP:0002105	0.00966148
HP	Abnormal nephron morphology	HP:0012575	0.01105467
HP	Anemia due to reduced life span of red cells	HP:0011895	0.0110574
HP	Abnormal sputum	HP:0032016	0.01618997
HP	Recurrent gram-negative bacterial infections	HP:0005420	0.01649476
HP	Chronic pulmonary obstruction	HP:0006510	0.01649476
HP	Recurrent respiratory infections	HP:0002205	0.01786054
HP	Hematuria	HP:0000790	0.02526861
HP	Abnormal urine cytology	HP:0012614	0.02526861
HP	Serositis	HP:0045073	0.02744858
HP	Abnormality of the common coagulation pathway	HP:0010990	0.02794282
**Downregulated**			
GO:MF	corticotropin-releasing hormone activity	GO:0017045	0.04989139

Supplementary Table 11C. TCX significant enrichment terms from g:Profiler. Terms were derived using gene lists split by upregulated and downregulated genes.

Source	Term Name	Term ID	Adjusted p-value
**Upregulated**			
GO:MF	extracellular matrix structural constituent	GO:0005201	2.44E-07
GO:MF	collagen binding	GO:0005518	7.3037E-05
GO:MF	cell adhesion molecule binding	GO:0050839	0.00025118
GO:MF	integrin binding	GO:0005178	0.00413614
GO:MF	structural molecule activity	GO:0005198	0.00873773
GO:MF	signaling receptor binding	GO:0005102	0.02438259
GO:BP	extracellular matrix organization	GO:0030198	2.62E-13
GO:BP	extracellular structure organization	GO:0043062	2.73E-13
GO:BP	external encapsulating structure organization	GO:0045229	2.85E-13
GO:BP	cell adhesion	GO:0007155	2.5911E-05
GO:BP	animal organ development	GO:0048513	4.1204E-05
GO:BP	collagen fibril organization	GO:0030199	0.00021282
GO:BP	supramolecular fiber organization	GO:0097435	0.00026438
GO:BP	circulatory system development	GO:0072359	0.00053934
GO:BP	blood vessel development	GO:0001568	0.00058933
GO:BP	vasculature development	GO:0001944	0.00085163
GO:BP	anatomical structure morphogenesis	GO:0009653	0.00142733
GO:BP	animal organ morphogenesis	GO:0009887	0.00176344
GO:BP	blood vessel morphogenesis	GO:0048514	0.00185905
GO:BP	extracellular matrix assembly	GO:0085029	0.00240907
GO:BP	tube development	GO:0035295	0.00302357
GO:BP	tube morphogenesis	GO:0035239	0.00311192
GO:BP	system development	GO:0048731	0.00406459
GO:BP	angiogenesis	GO:0001525	0.006383
GO:BP	tissue development	GO:0009888	0.00683537
GO:BP	response to endogenous stimulus	GO:0009719	0.0085974
GO:BP	multicellular organism development	GO:0007275	0.01064258
GO:BP	regulation of cell adhesion	GO:0030155	0.01156251

GO:BP	regulation of developmental process	GO:0050793	0.01205008
GO:BP	tissue morphogenesis	GO:0048729	0.01488227
GO:BP	regulation of anatomical structure morphogenesis	GO:0022603	0.01668052
GO:BP	negative regulation of cell migration	GO:0030336	0.01778987
GO:BP	negative regulation of cell motility	GO:2000146	0.02407287
GO:BP	negative regulation of locomotion	GO:0040013	0.03317264
GO:BP	cellular response to growth factor stimulus	GO:0071363	0.03424503
GO:BP	cellular component organization	GO:0016043	0.04415806
GO:BP	response to growth factor	GO:0070848	0.04739664
GO:BP	anatomical structure formation involved in morphogenesis	GO:0048646	0.04778375
GO:BP	L-arginine import across plasma membrane	GO:0097638	0.04843655
GO:CC	extracellular matrix	GO:0031012	1.19E-12
GO:CC	external encapsulating structure	GO:0030312	1.22E-12
GO:CC	collagen-containing extracellular matrix	GO:0062023	9.27E-12
GO:CC	extracellular space	GO:0005615	1.50E-07
GO:CC	extracellular region	GO:0005576	2.7509E-06
GO:CC	cell periphery	GO:0071944	7.0519E-06
GO:CC	extracellular exosome	GO:0070062	0.00085355
GO:CC	extracellular vesicle	GO:1903561	0.00100827
GO:CC	extracellular membrane-bounded organelle	GO:0065010	0.00101384
GO:CC	extracellular organelle	GO:0043230	0.00101384
GO:CC	basal part of cell	GO:0045178	0.03776256
GO:CC	microfibril	GO:0001527	0.04202146
KEGG	ECM-receptor interaction	KEGG:04512	0.00475116
MIRNA	hsa-mir-4665-5p	MIRNA:hsa-mir-4665-5p	0.00203096
MIRNA	hsa-mir-1275	MIRNA:hsa-mir-1275	0.00208098
MIRNA	hsa-mir-4646-5p	MIRNA:hsa-mir-4646-5p	0.03371536
HPA	Skin 1; extracellular matrix[≥Medium]	HPA:0461432	2.7236E-05
HPA	Skin 2; extracellular matrix[≥Medium]	HPA:0471432	3.7161E-05
HPA	Skin 1; extracellular matrix[≥Low]	HPA:0461431	8.4992E-05
HPA	Skin 2; extracellular matrix[≥Low]	HPA:0471431	0.00015987
HPA	Skin 1; extracellular matrix[High]	HPA:0461433	0.00896255
HPA	Skin 2; extracellular matrix[High]	HPA:0471433	0.01002027
CORUM	IGF2-IGFBP2 complex	CORUM:6191	0.04981949
HP	Scarring	HP:0100699	0.0012707
**Downregulated**			
GO:MF	glutamate decarboxylase activity	GO:0004351	7.1885E-05
GO:MF	hormone activity	GO:0005179	0.01303282
GO:MF	neuropeptide hormone activity	GO:0005184	0.02892697
GO:MF	neuropeptide activity	GO:0160041	0.03098298
GO:MF	neuropeptide receptor binding	GO:0071855	0.03990476
GO:MF	carboxy-lyase activity	GO:0016831	0.04732678
GO:BP	chemical synaptic transmission	GO:0007268	7.773E-05
GO:BP	anterograde trans-synaptic signaling	GO:0098916	7.773E-05
GO:BP	trans-synaptic signaling	GO:0099537	8.1953E-05
GO:BP	synaptic signaling	GO:0099536	0.00010258
GO:BP	cell-cell signaling	GO:0007267	0.0001359
GO:BP	gamma-aminobutyrate shunt	GO:0006540	0.00038281

GO:BP	forebrain development	GO:0030900	0.00290886
GO:BP	hormone-mediated apoptotic signaling pathway	GO:0008628	0.00382449
GO:BP	gamma-aminobutyric acid biosynthetic process	GO:0009449	0.00573491
GO:BP	succinate metabolic process	GO:0006105	0.00802634
GO:BP	gamma-aminobutyric acid metabolic process	GO:0009448	0.01069839
GO:BP	glutamate catabolic process	GO:0006538	0.01069839
GO:BP	response to steroid hormone	GO:0048545	0.03847696
GO:BP	brain development	GO:0007420	0.04816595
GO:CC	clathrin-sculpted gamma-aminobutyric acid transport vesicle membrane	GO:0061202	7.57E-07
GO:CC	clathrin-sculpted gamma-aminobutyric acid transport vesicle	GO:0061200	7.57E-07
GO:CC	clathrin-sculpted vesicle	GO:0060198	2.9684E-06
GO:CC	inhibitory synapse	GO:0060077	7.5468E-06
GO:CC	somatodendritic compartment	GO:0036477	9.6574E-06
GO:CC	neuronal cell body	GO:0043025	1.1777E-05
GO:CC	cell body	GO:0044297	2.4998E-05
GO:CC	GABA-ergic synapse	GO:0098982	3.1926E-05
GO:CC	synapse	GO:0045202	4.2036E-05
GO:CC	axon	GO:0030424	5.9888E-05
GO:CC	cell junction	GO:0030054	0.00069504
GO:CC	axon terminus	GO:0043679	0.00339921
GO:CC	neuron projection	GO:0043005	0.00387292
GO:CC	neuron projection terminus	GO:0044306	0.00498438
GO:CC	clathrin-coated vesicle membrane	GO:0030665	0.00521122
GO:CC	transport vesicle	GO:0030133	0.00835544
GO:CC	neuronal dense core vesicle	GO:0098992	0.0129252
GO:CC	cytoplasmic vesicle	GO:0031410	0.01406203
GO:CC	intracellular vesicle	GO:0097708	0.01427632
GO:CC	coated vesicle membrane	GO:0030662	0.01673285
GO:CC	secretory vesicle	GO:0099503	0.01961415
GO:CC	clathrin-coated vesicle	GO:0030136	0.02216607
GO:CC	dense core granule	GO:0031045	0.02549074
GO:CC	presynapse	GO:0098793	0.03182562
GO:CC	distal axon	GO:0150034	0.04547024
KEGG	GABAergic synapse	KEGG:04727	0.00183946
KEGG	Taurine and hypotaurine metabolism	KEGG:00430	0.00338082
KEGG	Neuroactive ligand-receptor interaction	KEGG:04080	0.00516456
KEGG	Butanoate metabolism	KEGG:00650	0.00984622
KEGG	beta-Alanine metabolism	KEGG:00410	0.01302362
KEGG	Alanine, aspartate and glutamate metabolism	KEGG:00250	0.01860919
KEGG	Type I diabetes mellitus	KEGG:04940	0.02176883
REAC	Transcriptional Regulation by MECP2	REAC:R-HSA-8986944	2.4114E-05
REAC	GABA synthesis, release, reuptake and degradation	REAC:R-HSA-888590	8.6193E-05
REAC	MECP2 regulates transcription of genes involved in GABA signaling	REAC:R-HSA-9022927	0.00016704
REAC	GABA synthesis	REAC:R-HSA-888568	0.00016704
REAC	Neurotransmitter release cycle	REAC:R-HSA-112310	0.00204335
REAC	MECP2 regulates transcription of neuronal ligands	REAC:R-HSA-9022702	0.00250135
WP	GABA receptor signaling	WP:WP4159	0.00016529

WP	GABA metabolism aka GHB	WP:WP4157	0.00356135
WP	Alanine and aspartate metabolism	WP:WP106	0.00427199
WP	Biogenic amine synthesis	WP:WP550	0.00588569
TF	Factor: NRSF; motif: TTCAGCACCACGGACAGMGCC	TF:M00256	0.03014929
HPA	Hypothalamus; neuronal projections[High]	HPA:0260143	0.00597302
HPA	Hypothalamus; neuronal projections[≥Medium]	HPA:0260142	0.018733
HPA	Hypothalamus; neuronal projections[≥Low]	HPA:0260141	0.0201133
HPA	Adrenal gland; medullary cells[≥Medium]	HPA:0020062	0.0230194
HPA	Adrenal gland; medullary cells[≥Low]	HPA:0020061	0.02774147
HPA	Hypothalamus	HPA:0260000	0.0365758

Supplementary Table 11D. IFG significant enrichment terms from g:Profiler.
Terms were derived using gene lists split by upregulated and downregulated genes.

Source	Term Name	Term ID	Adjusted p-value
	**Upregulated**		
GO:MF	myo-inositol transmembrane transporter activity	GO:0005365	0.002515918
GO:MF	D-glucose:sodium symporter activity	GO:0005412	0.008787461
GO:MF	signaling receptor activity	GO:0038023	0.012629775
GO:MF	molecular transducer activity	GO:0060089	0.012629775
GO:MF	polyol transmembrane transporter activity	GO:0015166	0.022951199
GO:MF	carbohydrate:monoatomic cation symporter activity	GO:0005402	0.049902526
GO:MF	death receptor activity	GO:0005035	0.049902526
GO:BP	myo-inositol transport	GO:0015798	0.017848842
GO:BP	humoral immune response mediated by circulating immunoglobulin	GO:0002455	0.047681533
GO:CC	cell periphery	GO:0071944	0.000660059
GO:CC	extracellular matrix	GO:0031012	0.004471785
GO:CC	external encapsulating structure	GO:0030312	0.004517023
GO:CC	collagen-containing extracellular matrix	GO:0062023	0.01126345
REAC	Inositol transporters	REAC:R-HSA-429593	0.002229598
CORUM	Neurotrophin-3-p75 complex	CORUM:5408	0.049931693
HP	Reticular pigmentary degeneration	HP:0007937	0.014132697
HP	Hyperpigmentation of the fundus	HP:0011512	0.049347622
	**Downregulated**		
GO:MF	neuropeptide hormone activity	GO:0005184	1.28E-07
GO:MF	neuropeptide activity	GO:0160041	1.48E-07
GO:MF	hormone activity	GO:0005179	5.03E-07
GO:MF	receptor ligand activity	GO:0048018	0.000534637
GO:MF	signaling receptor activator activity	GO:0030546	0.000569566
GO:MF	signaling receptor regulator activity	GO:0030545	0.000785334
GO:MF	peptide hormone receptor binding	GO:0051428	0.008281724
GO:MF	hormone receptor binding	GO:0051427	0.023378478
GO:MF	molecular function activator activity	GO:0140677	0.02341639
GO:BP	protein secretion	GO:0009306	0.000504204
GO:BP	establishment of protein localization to extracellular region	GO:0035592	0.000524271
GO:BP	protein localization to extracellular region	GO:0071692	0.000566306
GO:BP	negative regulation of glucagon secretion	GO:0070093	0.000710007
GO:BP	insulin secretion	GO:0030073	0.002295813

GO:BP	hormone-mediated apoptotic signaling pathway	GO:0008628	0.002365488
GO:BP	regulation of hormone levels	GO:0010817	0.003334016
GO:BP	regulation of epinephrine secretion	GO:0014060	0.003547333
GO:BP	peptide hormone secretion	GO:0030072	0.004872213
GO:BP	epinephrine secretion	GO:0048242	0.004965006
GO:BP	peptide secretion	GO:0002790	0.005342752
GO:BP	glucagon secretion	GO:0070091	0.006618328
GO:BP	regulation of glucagon secretion	GO:0070092	0.006618328
GO:BP	peptide transport	GO:0015833	0.007888917
GO:BP	positive regulation of secretion by cell	GO:1903532	0.009028553
GO:BP	positive regulation of secretion	GO:0051047	0.012258324
GO:BP	hormone secretion	GO:0046879	0.012561391
GO:BP	epinephrine transport	GO:0048241	0.012990396
GO:BP	cell-cell signaling	GO:0007267	0.013061873
GO:BP	amide transport	GO:0042886	0.013829422
GO:BP	hormone transport	GO:0009914	0.014328516
GO:BP	secretion by cell	GO:0032940	0.026997449
GO:BP	export from cell	GO:0140352	0.040327965
GO:CC	neuronal cell body	GO:0043025	0.000145883
GO:CC	cell body	GO:0044297	0.000273078
GO:CC	somatodendritic compartment	GO:0036477	0.00206636
GO:CC	perikaryon	GO:0043204	0.004304058
GO:CC	neuronal dense core vesicle	GO:0098992	0.008768914
GO:CC	dense core granule	GO:0031045	0.017306307
GO:CC	synapse	GO:0045202	0.04826044
KEGG	Neuroactive ligand-receptor interaction	KEGG:04080	0.002313659
KEGG	cAMP signaling pathway	KEGG:04024	0.016566685
REAC	MECP2 regulates transcription of neuronal ligands	REAC:R-HSA-9022702	0.001197465
REAC	Signaling by NTRK1 (TRKA)	REAC:R-HSA-187037	0.00782271
REAC	Signaling by NTRKs	REAC:R-HSA-166520	0.012551428
TF	Factor: ATF-6; motif: GRTGACGTCAYC; match class: 1	TF:M11226_1	0.001459547
TF	Factor: ZNF295; motif: RTGACGTCAN; match class: 1	TF:M12700_1	0.001500111
TF	Factor: CREBL1; motif: GRTGACGTCAYC; match class: 1	TF:M11231_1	0.00161648
TF	Factor: ATF-6; motif: GRTGACGTCAYC	TF:M11226	0.001705704
TF	Factor: CREB1; motif: TGACGTCA; match class: 1	TF:M02279_1	0.001763501
TF	Factor: NRSF; motif: GYRCTGTCCRYGGTGCTGA; match class: 1	TF:M01028_1	0.001850904
TF	Factor: jdp2; motif: NRTGACGTCAYN; match class: 1	TF:M11298_1	0.001933982
TF	Factor: XBP-1; motif: NNTGACGTCAYN; match class: 1	TF:M11277_1	0.002301185
TF	Factor: AIBZIP; motif: NRTGACGTCAYN; match class: 1	TF:M11257_1	0.002360166
TF	Factor: REST; motif: NGGCGCTGTCCRTGGTGCTGAA; match class: 1	TF:M12658_1	0.002535881
TF	Factor: REST; motif: TTCAGCACCAYGGACAGCKCCN; match class: 1	TF:M10047_1	0.00355743
TF	Factor: jdp2; motif: NRTGAYGTCAYN; match class: 1	TF:M11296_1	0.003633096
TF	Factor: AIBZIP; motif: NRTGACGTCAYN	TF:M11257	0.003873137
TF	Factor: XBP-1; motif: NNTGACGTCAYN	TF:M11277	0.003873137
TF	Factor: CREB; motif: NNTKACGTCANNNS; match class: 1	TF:M00916_1	0.004631774
TF	Factor: ATF-2; motif: NNNTGACGTNAN; match class: 1	TF:M01862_1	0.004699
TF	Factor: CREM; motif: NRTGATGTCAYN; match class: 1	TF:M11224_1	0.005078665

TF	Factor: CREBL1; motif: GRTGACGTCAYC	TF:M11231	0.006009647
TF	Factor: CREB1; motif: NRTGAYGTCAYN; match class: 1	TF:M11222_1	0.006894638
TF	Factor: CREB1; motif: NNNTGATGTCANN; match class: 1	TF:M12486_1	0.007224831
TF	Factor: CREB1; motif: NRTGACGTCANN; match class: 1	TF:M11223_1	0.007394114
TF	Factor: ATF; motif: NTGACGTCANYS; match class: 1	TF:M00338_1	0.008591726
TF	Factor: ATF-1; motif: TNACGTCAN; match class: 1	TF:M01861_1	0.009616146
TF	Factor: CREM; motif: TGACGTCASYN; match class: 1	TF:M01820_1	0.009649741
TF	Factor: CREB1; motif: NRTGACGTCANN	TF:M11223	0.010026653
TF	Factor: ATF-2; motif: NNTGACGTCAN; match class: 1	TF:M07312_1	0.010668991
TF	Factor: ATF-1; motif: NNNTGACGTNNN; match class: 1	TF:M07034_1	0.011247965
TF	Factor: NRSF; motif: TTCAGCACCACGGACAGMGCC	TF:M00256	0.011266834
TF	Factor: ATF-2; motif: NNTGACGTMANN; match class: 1	TF:M11307_1	0.011895675
TF	Factor: CREB1; motif: NNNNACGTCANN; match class: 1	TF:M03544_1	0.011908714
TF	Factor: CREB1; motif: NRTGATGTCAYN; match class: 1	TF:M11216_1	0.012898973
TF	Factor: ATF-2; motif: NRTGAYGTMAYN; match class: 1	TF:M11308_1	0.012959262
TF	Factor: CREB1; motif: NNNNTGACGTNANNN; match class: 1	TF:M07248_1	0.013264186
TF	Factor: ATF2:c-Jun; motif: TGACGTYA; match class: 1	TF:M00041_1	0.01421445
TF	Factor: ATF-4; motif: KACGTCAKS; match class: 1	TF:M01864_1	0.014946189
TF	Factor: CREBPA; motif: NRTGACGTMANN; match class: 1	TF:M11313_1	0.015148987
TF	Factor: ATF-1; motif: NRTGACGTMAN; match class: 1	TF:M09592_1	0.015993499
TF	Factor: ATF-2; motif: NNTGACGTMANN	TF:M11307	0.019003381
TF	Factor: ZNF295; motif: RTGACGTCAN	TF:M12700	0.021342447
TF	Factor: REST; motif: TTCAGCACCAYGGACAGCKCCN; match class: 1	TF:M09650_1	0.023156212
TF	Factor: ATF-4; motif: GGATGACGTCATCC; match class: 1	TF:M11335_1	0.026199133
TF	Factor: NRSE; motif: TTYAGCWCCDCGGASAGYRCC; match class: 1	TF:M00325_1	0.027314297
TF	Factor: ATF; motif: CNSTGACGTNNNYC; match class: 1	TF:M00017_1	0.029658669
TF	Factor: NRSF; motif: TTCAGCACCACGGACAGMGCC; match class: 1	TF:M00256_1	0.032402278
TF	Factor: ATF-4; motif: GGATGACGTCATCC	TF:M11335	0.033399067
TF	Factor: ATF-2; motif: NRTGACGTMANN; match class: 1	TF:M11309_1	0.036690905
TF	Factor: CREB; motif: TGACGTMA; match class: 1	TF:M00039_1	0.041658967
TF	Factor: CREB; motif: NSTGACGTMANN; match class: 1	TF:M00178_1	0.048339524
HPA	Hypothalamus; neuronal projections[≥Low]	HPA:0260141	5.34E-08
HPA	Hypothalamus	HPA:0260000	1.85E-07
HPA	Hypothalamus; synapses[≥Low]	HPA:0260151	1.81599E-06
HPA	Hypothalamus; neuronal projections[High]	HPA:0260143	4.61874E-06
HPA	Hypothalamus; neuronal projections[≥Medium]	HPA:0260142	2.69532E-05
HPA	Adrenal gland; medullary cells[≥Medium]	HPA:0020062	3.69598E-05
HPA	Adrenal gland; medullary cells[≥Low]	HPA:0020061	4.91727E-05
HPA	Hypothalamus; synapses[≥Medium]	HPA:0260152	0.001020745
HPA	Pituitary gland; cells in anterior [High]	HPA:0370493	0.014200499
HPA	Pituitary gland; cells in anterior [≥Medium]	HPA:0370492	0.032114643
HPA	Pituitary gland	HPA:0370000	0.035791478
HPA	Pituitary gland; cells in anterior [≥Low]	HPA:0370491	0.035791478

Supplementary Table 11E. DLPFC significant enrichment terms from g:Profiler. Terms were derived using gene lists split by upregulated and downregulated genes.

Source	Term Name	Term ID	Adjusted p-value
	**Upregulated**		
GO:CC	extracellular matrix	GO:0031012	0.027159637
GO:CC	external encapsulating structure	GO:0030312	0.027425895
TF	Factor: NF1B; motif: CYTGGCNYNCWGCCAN	TF:M09762	0.0256336
CORUM	KCNQ1-KCNE4 complex	CORUM:6708	0.049931693
	**Downregulated**		
GO:MF	hormone activity	GO:0005179	0.003807714
GO:MF	neuropeptide hormone activity	GO:0005184	0.011931949
GO:MF	neuropeptide activity	GO:0160041	0.012781284
GO:BP	hormone-mediated apoptotic signaling pathway	GO:0008628	0.001318792
GO:CC	neuronal dense core vesicle	GO:0098992	0.006824224
GO:CC	dense core granule	GO:0031045	0.013478
KEGG	Neuroactive ligand-receptor interaction	KEGG:04080	7.03712E-06
REAC	MECP2 regulates transcription of neuronal ligands	REAC:R-HSA-9022702	0.00083627
REAC	GPCR ligand binding	REAC:R-HSA-500792	0.009524038
REAC	GPCR downstream signalling	REAC:R-HSA-388396	0.03117468
REAC	Signaling by GPCR	REAC:R-HSA-372790	0.048104952
TF	Factor: NRSF; motif: TTCAGCACCACGGACAGMGCC; match class: 1	TF:M00256_1	0.025232116
HPA	Hypothalamus; neuronal projections[High]	HPA:0260143	0.001695551
HPA	Hypothalamus; neuronal projections[≥Medium]	HPA:0260142	0.005329326
HPA	Hypothalamus; neuronal projections[≥Low]	HPA:0260141	0.005723048
HPA	Adrenal gland; medullary cells[≥Medium]	HPA:0020062	0.006552335
HPA	Adrenal gland; medullary cells[≥Low]	HPA:0020061	0.007900755
HPA	Hypothalamus	HPA:0260000	0.010426253

Supplementary Table 11F. CER significant enrichment terms from g:Profiler. Terms were derived using gene lists split by upregulated and downregulated genes.

Source	Term Name	Term ID	Adjusted p-value
	**Upregulated**		
GO:MF	RAGE receptor binding	GO:0050786	0.000309305
GO:MF	Toll-like receptor 4 binding	GO:0035662	0.0085202
GO:MF	C3HC4-type RING finger domain binding	GO:0055131	0.014183035
GO:MF	structural constituent of chromatin	GO:0030527	0.016519548
GO:MF	ATP-dependent protein disaggregase activity	GO:0140545	0.021248639
GO:MF	icosatetraenoic acid binding	GO:0050543	0.029711864
GO:MF	arachidonate binding	GO:0050544	0.029711864
GO:MF	icosanoid binding	GO:0050542	0.039567576
GO:BP	multicellular organism development	GO:0007275	3.07767E-06
GO:BP	anatomical structure development	GO:0048856	0.000104571
GO:BP	developmental process	GO:0032502	0.000134684
GO:BP	regulation of multicellular organismal process	GO:0051239	0.000228981
GO:BP	regulation of multicellular organismal development	GO:2000026	0.000972326
GO:BP	tissue development	GO:0009888	0.001594282
GO:BP	multicellular organismal process	GO:0032501	0.00181104
GO:BP	chemotaxis	GO:0006935	0.002373745

GO:BP	taxis	GO:0042330	0.002450631
GO:BP	regulation of nucleotide-binding domain, leucine rich repeat containing receptor signaling pathway	GO:0070424	0.003814183
GO:BP	neutrophil aggregation	GO:0070488	0.0049695
GO:BP	regulation of molecular function	GO:0065009	0.011951781
GO:BP	cell adhesion	GO:0007155	0.015036249
GO:BP	regulation of developmental process	GO:0050793	0.01720864
GO:BP	regulation of cell differentiation	GO:0045595	0.020318701
GO:BP	positive regulation of signal transduction	GO:0009967	0.020581486
GO:BP	response to stress	GO:0006950	0.020911142
GO:BP	regulation of pattern recognition receptor signaling pathway	GO:0062207	0.021782265
GO:BP	biological regulation	GO:0065007	0.022122943
GO:BP	regulation of response to external stimulus	GO:0032101	0.024641456
GO:BP	positive regulation of developmental process	GO:0051094	0.026186081
GO:BP	cell differentiation	GO:0030154	0.028495974
GO:BP	cellular developmental process	GO:0048869	0.028596183
GO:BP	nucleotide-binding domain, leucine rich repeat containing receptor signaling pathway	GO:0035872	0.030100138
GO:BP	system development	GO:0048731	0.030926365
GO:BP	positive regulation of intracellular signal transduction	GO:1902533	0.031473723
GO:BP	positive regulation of cell differentiation	GO:0045597	0.031942528
GO:BP	circulatory system development	GO:0072359	0.040186405
GO:BP	positive regulation of defense response	GO:0031349	0.046310629
GO:BP	negative regulation of extrinsic apoptotic signaling pathway	GO:2001237	0.047161378
GO:BP	regulation of toll-like receptor signaling pathway	GO:0034121	0.048987124
GO:BP	heat acclimation	GO:0010286	0.049525023
GO:BP	cellular heat acclimation	GO:0070370	0.049525023
GO:CC	calprotectin complex	GO:1990660	0.000651091
GO:CC	collagen-containing extracellular matrix	GO:0062023	0.001139479
GO:CC	extracellular space	GO:0005615	0.003652706
GO:CC	extracellular matrix	GO:0031012	0.010326896
GO:CC	external encapsulating structure	GO:0030312	0.010444258
GO:CC	protein-DNA complex	GO:0032993	0.013688915
GO:CC	nucleosome	GO:0000786	0.020990412
GO:CC	extracellular region	GO:0005576	0.025811113
GO:CC	secretory granule	GO:0030141	0.032825564
GO:CC	extracellular exosome	GO:0070062	0.039596175
GO:CC	extracellular vesicle	GO:1903561	0.045247742
GO:CC	extracellular organelle	GO:0043230	0.045447523
GO:CC	extracellular membrane-bounded organelle	GO:0065010	0.045447523
KEGG	MAPK signaling pathway	KEGG:04010	0.005803538
REAC	DNA Damage/Telomere Stress Induced Senescence	REAC:R-HSA-2559586	0.026370471
REAC	Metal sequestration by antimicrobial proteins	REAC:R-HSA-6799990	0.042363194
WP	Hypothesized pathways in pathogenesis of cardiovascular disease	WP:WP3668	0.012261174
MIRNA	hsa-mir-6874-3p	MIRNA:hsa-mir-6874-3p	0.044622041
HPA	Skin 2; cells in corneal layer[≥Medium]	HPA:0471402	0.014616972
CORUM	Calprotectin heterotetramer	CORUM:6826	0.002391614
CORUM	iNOS-S100A8/A9 complex	CORUM:6827	0.007162108

Downregulated			
GO:MF	haptoglobin binding	GO:0031720	0.003226317
GO:MF	oxygen carrier activity	GO:0005344	0.007515687
GO:MF	oxygen binding	GO:0019825	0.039904757
GO:BP	nitric oxide transport	GO:0030185	0.003824489
GO:BP	carbon dioxide transport	GO:0015670	0.040029986
GO:BP	oxygen transport	GO:0015671	0.04573405
GO:CC	haptoglobin-hemoglobin complex	GO:0031838	0.001643595
GO:CC	hemoglobin complex	GO:0005833	0.002329514
GO:CC	endocytic vesicle lumen	GO:0071682	0.007533275
KEGG	African trypanosomiasis	KEGG:05143	0.04731037
REAC	Erythrocytes take up oxygen and release carbon dioxide	REAC:R-HSA-1247673	0.005995612
REAC	O2/CO2 exchange in erythrocytes	REAC:R-HSA-1480926	0.012968462
REAC	Erythrocytes take up carbon dioxide and release oxygen	REAC:R-HSA-1237044	0.012968462
CORUM	CHRNA2-CHRNB4 complex	CORUM:6440	0.049931693
HP	Heinz body anemia	HP:0005511	0.003418002
HP	Imbalanced hemoglobin synthesis	HP:0005560	0.006832413
HP	Reduced alpha/beta synthesis ratio	HP:0011907	0.006832413
HP	Heinz bodies	HP:0020082	0.017063095
HP	Nonspherocytic hemolytic anemia	HP:0001930	0.040886919

Supplementary Table 12. Identification of functional modules for each region. Using MCODE (version 2.0.3) functional clusters of highly interconnected genes were identified for each region to quantify the extent of dysregulation occurring across regions. For each module, the number of clusters, nodes, edges, and the MCODE score, which measures cluster density and interconnectedness, were recorded.

Region	Cluster ID	Number of Nodes	Number of Edges	MCODE score	Genes
PHG	1	29	212	12.571	*HSPB1, SPHK1, ITPKB, COLEC12, MFAP4, STON1, COL1A2, AEBP1, AHNAK, EPHA2, MYOF, KLF4, CDKN1A, SPARC, CCN1, BGN, TRIP10, ANXA2, ANXA1, ID3, SERPINH1, JUNB, MYH11, TGIF1, MMP14, C11orf96, DUSP1, FBLN1, RGS4*
	2	18	130	11.647	*ZFP36, FOS, MAFF, TNC, BTG2, MYL9, ZFP36L1, ITGA5, C1R, RASL12, SERPINE1, COL6A2, TGFBI, EMP3, ATF3, GEM, CD44 IER3*
	3	32	157	8.258	*SRPX, ITGAL, FZD7, NFKB2, CFI, PCSK1, APOL1, LATS2, NLRC5, CHI3L1, INHBA, GBP3, APLNR, GBP4, TNFRSF11B, TEAD3, SMTN, ITGAX, GBP1, NGFR, S100A4, WWTR1, EMP1, GBP2, PRELP, PDYN, PDLIM4, PTAFR, PLAUR, MCHR2, OLR1, CSF1*
	4	20	41	4.00	*C3AR1, TRIM47, ICAM1, VIP, PLEKHA4, TLR5, KCNE4, SLC13A4, COPZ2, STAT4, APOL3, VAMP8, KANK2, PIK3R5, SYTL4, PTPN6, RAB20, ITGA10, PPP1R13L, C5AR1*
	5	6	11	3.60	*PLA1A, OAS2, PLEK, MS4A7, HLA-DMB, CASP1*
	6	8	12	3.143	*PARVG, SLC4A11, CXCR4, COL27A1, SPN, SERPINA1, CIITA, ZIC4*

TCX	1	8	28	5.429	*GAS1, IGFBP5, GEM, COL1A2, ANGPT2, ECM2, DCN, IGF2*
	2	12	30	5.091	*S100A4, FOXC1, PCSK1, ANXA2, MFAP4, TGFBI, CYP1B1, COL6A2, OLFML2A, KCNE4, AEBP1, PDLIM1*
	3	4	6	4.00	*CRH, GFAP, NEUROD6, SLC13A4*
	4	3	6	3.00	*GAD1, GAD2, TAC1*
STG	1	39	787	34.211	*CSF1R, CSF3R, UCP2, CTSS, PARVG, ALOX5AP, LGALS9, MS4A7, LAIR1, HLA-DRB1, FCER1G, PTPRC, ITGB2, CD84, MYO1F, SLC7A7, SELPLG, CYBB, LILRB1, VSIG4, C1QC, HLA-DMB, MS4A6A, C1QA, HLA-DPA1, CD163, C3AR1, C1QB, BIN2, TYROBP, PTAFR, PLEK, LAPTM5, HLA-DRA, CD37, CD74, FCGR2A, CD300A, HK3*
	2	13	41	6.33	*GPSM3, TREM2, CD14, MFNG, VAMP8, IFITM1, OLR1, HMOX1, ITGAL, ABI3, SASH3, CASP1, SYK*
	3	12	38	5.250	*C3, SERPINH1, NGFR, C4A, SERPINE1, PLP2, NUPR1, MT1F, MT1G, CFI, S100A10, IER3*
	4	9	22	5.250	*F13A1, S100A4, HLA-DOA, EMP3, WAS, CX3CR1, SERPINA1, TCIRG1, S100A11*
IFG	1	5	8	3.500	*CARTPT, ADCYAP1, PCSK1, CRH, NGFR*
	2	3	4	3.000	*AZGP1, CFI, C4B*
DLPFC	1	6	8	3.200	*MAFF, KCNE4, SLC6A12, SPP1, CA12, PIEZO2*
	2	6	6	2.400	*HILPDA, CRH, ANGPT2, VGF, SLCO4A1, MT1H*
CER	1	8	26	6.286	*DUSP1, SERPINE1, CCN1, GEM, CCN2, PDLIM1, MAFF, RGS1*
	2	4	5	3.333	*CP, DSP, HGF, S100A8*
	3	12	18	3.091	*MT1H, TM4SF1, PLA1A, H4C5, GJA4, BIRC3, S100A9, KLK7, P2RY13, H4C8, HBB, H2BC5*
	4	3	3	3.000	*CX3CR1, ANGPT2, GPR4*

Supplementary Table 13A. ClueGO functional enrichment analysis of PHG. This was performed using genes identified from functional clusters.

Source	Term Name	Term ID	Number of Genes	% Associated Genes	Adjusted p-value
GO:BP	response to stress	GO:0006950	66	1.5691869	1.54E-15
GO:BP	response to wounding	GO:0009611	26	4.200323	2.85E-13
GO:BP	cell adhesion	GO:0007155	37	2.3241205	1.21E-11
GO:BP	defense response	GO:0006952	40	2.1253984	1.44E-11
GO:CC	extracellular matrix	GO:0031012	24	3.9215686	1.97E-11
GO:BP	tissue development	GO:0009888	41	1.9711539	7.55E-11
GO:CC	collagen-containing extracellular matrix	GO:0062023	20	4.3010755	6.11E-10
GO:BP	animal organ development	GO:0048513	53	1.4054627	3.72E-09
GO:BP	circulatory system development	GO:0072359	29	2.445194	4.46E-09
GO:CC	cell periphery	GO:0071944	73	1.0970844	4.59E-09

GO:BP	wound healing	GO:0042060	19	4.0339704	7.07E-09
GO:BP	anatomical structure development	GO:0048856	69	1.1285574	7.61E-09
GO:BP	anatomical structure morphogenesis	GO:0009653	45	1.5619576	1.09E-08
GO:BP	anatomical structure formation involved in morphogenesis	GO:0048646	29	2.3368251	1.32E-08
GO:BP	response to external stimulus	GO:0009605	46	1.5216672	1.42E-08
GO:BP	response to organic substance	GO:0010033	47	1.4728925	2.40E-08
GO:BP	regulation of multicellular organismal development	GO:2000026	31	2.120383	2.70E-08
GO:BP	blood vessel development	GO:0001568	22	2.9333334	7.13E-08
GO:CC	vesicle	GO:0031982	54	1.277804	1.14E-07
GO:BP	regulation of developmental process	GO:0050793	41	1.5630957	1.26E-07
GO:BP	angiogenesis	GO:0001525	19	3.3687944	1.47E-07
GO:BP	inflammatory response	GO:0006954	23	2.655889	1.79E-07
GO:BP	response to cytokine	GO:0034097	24	2.534319	1.80E-07
GO:BP	response to biotic stimulus	GO:0009607	32	1.9002376	1.97E-07
GO:BP	cellular response to stimulus	GO:0051716	77	0.981642	2.31E-07
GO:BP	regulation of multicellular organismal process	GO:0051239	43	1.469583	2.64E-07
GO:BP	cell surface receptor signaling pathway	GO:0007166	44	1.4393196	2.92E-07
GO:BP	regulation of response to stimulus	GO:0048583	53	1.2502949	3.58E-07
GO:BP	regulation of cell adhesion	GO:0030155	22	2.676399	3.87E-07
GO:BP	cellular response to chemical stimulus	GO:0070887	45	1.399689	4.14E-07
GO:BP	cellular response to organic substance	GO:0071310	39	1.5372487	6.65E-07
GO:BP	cell population proliferation	GO:0008283	35	1.6666666	7.78E-07
GO:BP	multicellular organism development	GO:0007275	58	1.1457921	1.03E-06
GO:BP	regulation of immune system process	GO:0002682	30	1.8598884	1.30E-06
GO:BP	positive regulation of biological process	GO:0048518	68	1.0346926	1.30E-06
GO:BP	positive regulation of multicellular organismal process	GO:0051240	30	1.8564357	1.35E-06
GO:BP	cell death	GO:0008219	36	1.5887026	1.51E-06
GO:BP	response to other organism	GO:0051707	30	1.8382353	1.68E-06
GO:BP	cell communication	GO:0007154	69	1.0026156	2.40E-06
GO:BP	positive regulation of angiogenesis	GO:0045766	11	5.978261	2.84E-06
GO:BP	tube morphogenesis	GO:0035239	22	2.3887079	3.03E-06
GO:BP	cellular response to cytokine stimulus	GO:0071345	21	2.4561403	4.30E-06
GO:BP	positive regulation of response to stimulus	GO:0048584	36	1.5157895	5.18E-06
GO:BP	positive regulation of cellular process	GO:0048522	62	1.0533469	6.04E-06
GO:BP	positive regulation of cell migration	GO:0030335	17	3.0141845	6.65E-06
GO:CC	cytoplasmic vesicle	GO:0031410	38	1.4426727	6.66E-06
GO:BP	tube development	GO:0035295	24	2.0797226	8.13E-06
GO:BP	regulation of angiogenesis	GO:0045765	13	4.180064	8.39E-06
GO:BP	regulation of anatomical structure morphogenesis	GO:0022603	22	2.2357724	9.71E-06
GO:BP	response to chemical	GO:0042221	53	1.1474345	1.01E-05
GO:BP	system development	GO:0048731	53	1.1444613	1.06E-05
GO:BP	regulation of cell population proliferation	GO:0042127	30	1.6806723	1.26E-05

GO:BP	epithelium development	GO:0060429	25	1.9516003	1.34E-05
GO:BP	signal transduction	GO:0007165	64	1.010101	1.37E-05
GO:BP	cell-substrate adhesion	GO:0031589	14	3.617571	1.41E-05
GO:BP	cell adhesion mediated by integrin	GO:0033627	8	8.888889	1.53E-05
GO:BP	extracellular matrix organization	GO:0030198	13	3.7790697	2.66E-05
GO:BP	regulation of cell motility	GO:2000145	22	2.1093001	2.66E-05
GO:BP	positive regulation of metabolic process	GO:0009893	48	1.1851852	3.18E-05
GO:BP	regulation of cell death	GO:0010941	29	1.6533637	3.19E-05
GO:BP	defense response to other organism	GO:0098542	24	1.9215373	3.47E-05
GO:BP	cell-cell adhesion	GO:0098609	21	2.1298175	4.63E-05
GO:BP	positive regulation of macromolecule metabolic process	GO:0010604	45	1.2080537	4.89E-05
GO:BP	regulation of programmed cell death	GO:0043067	27	1.7023959	5.48E-05
GO:CC	vesicle membrane	GO:0012506	24	1.8706157	5.65E-05
GO:BP	apoptotic process	GO:0006915	31	1.5240904	6.22E-05
GO:BP	regulation of body fluid levels	GO:0050878	14	3.1674209	6.96E-05
GO:CC	extracellular region	GO:0005576	51	1.1043742	7.80E-05
GO:BP	immune response	GO:0006955	31	1.5048543	8.18E-05
GO:BP	positive regulation of gene expression	GO:0010628	23	1.8760196	1.01E-04
GO:BP	cell activation	GO:0001775	23	1.8533441	1.24E-04
GO:BP	positive regulation of immune system process	GO:0002684	21	2.0038168	1.24E-04
GO:BP	regulation of cytokine production	GO:0001817	18	2.2727273	1.57E-04
GO:BP	mononuclear cell migration	GO:0071674	10	4.5662103	1.59E-04
GO:MF	integrin binding	GO:0005178	9	5.357143	1.61E-04
GO:MF	protein-containing complex binding	GO:0044877	24	1.7569547	1.71E-04
GO:CC	secretory granule membrane	GO:0030667	12	3.5294118	1.72E-04
GO:BP	cytokine production	GO:0001816	18	2.255639	1.73E-04
GO:BP	cellular response to interferon-gamma	GO:0071346	8	6.2992125	2.07E-04
GO:BP	cellular developmental process	GO:0048869	49	1.0939941	2.08E-04
GO:BP	positive regulation of developmental process	GO:0051094	24	1.7021277	2.99E-04
GO:BP	negative regulation of cell adhesion	GO:0007162	11	3.618421	4.06E-04
GO:BP	negative regulation of biological process	GO:0048519	56	0.99750626	4.55E-04
GO:MF	cell adhesion molecule binding	GO:0050839	15	2.508361	4.56E-04
GO:BP	regulation of leukocyte migration	GO:0002685	10	4	5.14E-04
GO:BP	cell-matrix adhesion	GO:0007160	10	4	5.14E-04
GO:BP	negative regulation of cellular process	GO:0048523	52	1.0387535	5.35E-04
GO:BP	intracellular signal transduction	GO:0035556	36	1.2793177	5.57E-04
GO:CC	cytoplasmic vesicle membrane	GO:0030659	22	1.7446471	6.09E-04
GO:BP	positive regulation of signaling	GO:0023056	27	1.4786419	8.09E-04
GO:BP	regulation of phosphate metabolic process	GO:0019220	24	1.6032064	8.33E-04
GO:CC	endomembrane system	GO:0012505	51	1.033016	8.36E-04
GO:CC	plasma membrane	GO:0005886	59	0.957326	8.66E-04
GO:BP	regulation of signaling	GO:0023051	41	1.1523328	8.67E-04
GO:BP	positive regulation of leukocyte migration	GO:0002687	8	5.0314465	0.001081578

GO:CC	extracellular space	GO:0005615	41	1.1297878	0.001235659
GO:BP	regulation of response to external stimulus	GO:0032101	19	1.8554688	0.001387482
GO:BP	leukocyte cell-cell adhesion	GO:0007159	12	2.8639617	0.001416534
GO:BP	MAPK cascade	GO:0000165	17	2.0262218	0.001478843
GO:BP	skeletal system development	GO:0001501	14	2.4263432	0.001479149
GO:BP	regulation of apoptotic process	GO:0042981	24	1.5463917	0.001510513
GO:BP	ERK1 and ERK2 cascade	GO:0070371	11	3.133903	0.001531915
GO:BP	regulation of cell differentiation	GO:0045595	25	1.4997001	0.0015665
GO:BP	leukocyte migration	GO:0050900	12	2.7972028	0.001761076
GO:BP	cell motility	GO:0048870	26	1.4508928	0.001761117
GO:BP	regulation of cell communication	GO:0010646	40	1.1280316	0.001779075
GO:BP	tissue morphogenesis	GO:0048729	15	2.218935	0.001894132
GO:BP	regulation of macromolecule metabolic process	GO:0060255	60	0.9226511	0.001998154
GO:BP	regulation of signal transduction	GO:0009966	37	1.1719987	0.002001763
GO:BP	positive regulation of cell communication	GO:0010647	26	1.427787	0.002307458
GO:BP	muscle structure development	GO:0061061	15	2.1645021	0.002516178
GO:BP	regulation of biological process	GO:0050789	91	0.74743325	0.002699703
GO:BP	innate immune response	GO:0045087	18	1.8367347	0.002798079
GO:BP	leukocyte activation	GO:0045321	19	1.7560074	0.002888171
GO:BP	regulation of metabolic process	GO:0019222	63	0.8909631	0.003051828
GO:BP	negative regulation of developmental process	GO:0051093	18	1.8145162	0.003268402
GO:CC	focal adhesion	GO:0005925	12	2.6200874	0.00327245
GO:BP	leukocyte differentiation	GO:0002521	14	2.2435896	0.003381247
GO:BP	blood coagulation	GO:0007596	9	3.6144578	0.003570452
GO:BP	hematopoietic or lymphoid organ development	GO:0048534	18	1.7699115	0.004511605
GO:BP	negative regulation of cell communication	GO:0010648	22	1.5256588	0.004701447
GO:BP	negative regulation of signaling	GO:0023057	22	1.520387	0.004944353
GO:BP	regulation of gene expression	GO:0010468	50	0.98058444	0.004965437
GO:MF	protein binding	GO:0005515	100	0.696767	0.005134216
GO:BP	animal organ morphogenesis	GO:0009887	19	1.6799293	0.005182009
GO:CC	secretory vesicle	GO:0099503	19	1.6799293	0.005182009
GO:CC	endoplasmic reticulum lumen	GO:0005788	10	2.9940119	0.005783933
GO:BP	negative regulation of response to stimulus	GO:0048585	24	1.4101057	0.006513428
GO:BP	chemotaxis	GO:0006935	14	2.0989506	0.006856767
GO:BP	gastrulation	GO:0007369	8	3.8095238	0.007383813
GO:BP	regulation of hemopoiesis	GO:1903706	11	2.5700934	0.008869673
GO:BP	regulation of immune response	GO:0050776	17	1.7435898	0.009317263
GO:BP	myeloid cell differentiation	GO:0030099	11	2.5522041	0.009387004

Supplementary Table 13B. ClueGO functional enrichment analysis of STG. This was performed using genes identified from functional clusters.

Source	Term Name	Term ID	Number of Genes	% Associated Genes	Adjusted p-value
GO:BP	microglial cell activation	GO:0001774	6	12.5	5.46E-07
GO:BP	positive regulation of cytokine production	GO:0001819	22	4.238921	4.57E-16
GO:BP	cell killing	GO:0001906	9	4.455446	1.03E-06
GO:BP	leukocyte mediated cytotoxicity	GO:0001909	7	4.861111	6.01E-06
GO:BP	immune effector process	GO:0002252	31	4.0522876	4.63E-23
GO:BP	myeloid leukocyte activation	GO:0002274	17	6.589147	2.88E-15
GO:BP	myeloid cell activation involved in immune response	GO:0002275	12	11.009174	3.28E-13
GO:BP	lymphocyte activation involved in immune response	GO:0002285	10	4.6296296	1.95E-07
GO:BP	T cell activation involved in immune response	GO:0002286	8	6.6115704	3.80E-07
GO:BP	leukocyte activation involved in immune response	GO:0002366	20	6.3694267	6.95E-18
GO:BP	leukocyte mediated immunity	GO:0002443	26	5.189621	1.45E-21
GO:BP	myeloid leukocyte mediated immunity	GO:0002444	11	8.870968	5.01E-11
GO:BP	lymphocyte mediated immunity	GO:0002449	17	4.336735	2.77E-12
GO:BP	humoral immune response mediated by circulating immunoglobulin	GO:0002455	8	5.633803	1.03E-06
GO:BP	T cell mediated immunity	GO:0002456	6	4.878049	1.22E-05
GO:BP	antigen processing and presentation of peptide antigen via MHC class II	GO:0002495	9	25.714285	2.86E-13
GO:BP	regulation of leukocyte migration	GO:0002685	11	4.4	6.57E-08
GO:BP	positive regulation of leukocyte migration	GO:0002687	9	5.6603775	1.97E-07
GO:BP	regulation of immune effector process	GO:0002697	22	5.378973	3.01E-18
GO:BP	negative regulation of immune effector process	GO:0002698	8	6.25	5.47E-07
GO:BP	positive regulation of immune effector process	GO:0002699	19	6.6901407	2.54E-17
GO:BP	regulation of leukocyte mediated immunity	GO:0002703	18	7.142857	7.48E-17
GO:BP	positive regulation of leukocyte mediated immunity	GO:0002705	9	6	1.30E-07
GO:BP	regulation of lymphocyte mediated immunity	GO:0002706	9	4.864865	5.86E-07
GO:BP	positive regulation of lymphocyte mediated immunity	GO:0002708	6	4.83871	6.38E-06
GO:BP	regulation of T cell mediated immunity	GO:0002709	6	6.451613	5.96E-06
GO:BP	regulation of adaptive immune response based on somatic recombination of immune receptors built from immunoglobulin superfamily domains	GO:0002822	8	4.188482	3.51E-06
GO:BP	positive regulation of adaptive immune response based on somatic recombination of immune receptors built from immunoglobulin superfamily domains	GO:0002824	6	5.042017	1.51E-05
GO:BP	regulation of myeloid leukocyte mediated immunity	GO:0002886	11	16.666666	4.18E-14
GO:BP	complement activation	GO:0006956	8	5.16129	1.62E-06
GO:BP	immunoglobulin mediated immune response	GO:0016064	11	4.8245616	2.76E-08

GO:BP	antigen processing and presentation	GO:0019882	10	8.77193	6.02E-10
GO:MF	MHC protein complex binding	GO:0023023	7	18.421053	3.52E-09
GO:BP	leukocyte chemotaxis	GO:0030595	11	4.1984735	1.01E-07
GO:CC	secretory granule membrane	GO:0030667	15	4.4117646	7.45E-11
GO:BP	regulation of cell killing	GO:0031341	8	7.4074073	1.84E-07
GO:BP	tumor necrosis factor production	GO:0032640	12	6.703911	1.19E-10
GO:BP	regulation of chemokine production	GO:0032642	7	6.930693	1.51E-06
GO:BP	regulation of tumor necrosis factor production	GO:0032680	12	6.703911	1.19E-10
GO:BP	positive regulation of tumor necrosis factor production	GO:0032760	9	7.894737	1.45E-08
GO:CC	specific granule membrane	GO:0035579	7	7.216495	1.21E-06
GO:BP	macrophage activation	GO:0042116	10	8.77193	6.02E-10
GO:BP	neutrophil activation	GO:0042119	7	15.217391	1.40E-08
GO:BP	regulation of T cell proliferation	GO:0042129	9	4.6391754	8.07E-07
GO:CC	MHC class II protein complex	GO:0042613	6	33.333332	1.69E-09
GO:BP	leukocyte degranulation	GO:0043299	8	8.888889	5.24E-08
GO:BP	regulation of leukocyte degranulation	GO:0043300	8	16	5.48E-10
GO:BP	mast cell degranulation	GO:0043303	6	9.67742	1.58E-06
GO:BP	regulation of mast cell degranulation	GO:0043304	6	18.75	6.25E-08
GO:BP	regulation of T cell differentiation	GO:0045580	8	4.324324	3.15E-06
GO:BP	regulation of alpha-beta T cell activation	GO:0046634	8	6.779661	3.24E-07
GO:BP	positive regulation of alpha-beta T cell activation	GO:0046635	6	8.219178	2.54E-06
GO:BP	regulation of lymphocyte proliferation	GO:0050670	12	4.7244096	6.06E-09
GO:BP	positive regulation of lymphocyte proliferation	GO:0050671	7	4.516129	6.56E-06
GO:BP	negative regulation of lymphocyte proliferation	GO:0050672	6	6.451613	5.96E-06
GO:BP	negative regulation of cell activation	GO:0050866	12	5.240175	1.91E-09
GO:BP	positive regulation of T cell activation	GO:0050870	11	4.230769	9.63E-08
GO:BP	positive regulation of chemotaxis	GO:0050921	8	5.0955415	1.56E-06
GO:BP	negative regulation of secretion	GO:0051048	8	4.5454545	2.96E-06
GO:BP	negative regulation of lymphocyte activation	GO:0051250	8	4.7058825	2.68E-06
GO:BP	regulation of leukocyte proliferation	GO:0070663	13	4.5454545	1.69E-09
GO:BP	positive regulation of leukocyte proliferation	GO:0070665	8	4.519774	2.81E-06
GO:CC	tertiary granule	GO:0070820	10	5.586592	4.12E-08
GO:CC	tertiary granule membrane	GO:0070821	8	10.526316	1.47E-08
GO:BP	myeloid leukocyte migration	GO:0097529	11	4.1509433	1.11E-07
GO:BP	granulocyte migration	GO:0097530	8	4.597701	2.96E-06
GO:BP	synapse pruning	GO:0098883	6	54.545456	5.04E-11
GO:BP	neuroinflammatory response	GO:0150076	7	9.722222	2.32E-07
GO:BP	positive regulation of leukocyte differentiation	GO:1902107	9	4.736842	7.06E-07
GO:BP	regulation of leukocyte cell-cell adhesion	GO:1903037	16	4.1994753	2.73E-11

GO:BP	positive regulation of leukocyte cell-cell adhesion	GO:1903039	13	4.5774646	1.58E-09

Supplementary Table 13C. ClueGO functional enrichment analysis of TCX. This was performed using genes identified from functional clusters.

Source	Term Name	Term ID	Number of Genes	% Associated Genes	Adjusted p-value
GO:BP	angiogenesis	GO:0001525	6	1.0638298	0.04055427
GO:BP	blood vessel development	GO:0001568	7	0.93333334	0.02125857
GO:MF	glutamate decarboxylase activity	GO:0004351	2	100	9.21E-04
GO:MF	signaling receptor binding	GO:0005102	10	0.5896226	0.01754202
GO:MF	integrin binding	GO:0005178	4	2.3809524	0.035452
GO:MF	extracellular matrix structural constituent	GO:0005201	6	3.2432432	6.81E-05
GO:MF	protein binding	GO:0005515	27	0.18812709	0.04257349
GO:MF	collagen binding	GO:0005518	5	6.9444447	1.92E-05
GO:CC	extracellular region	GO:0005576	16	0.34647033	0.02142263
GO:BP	glutamate decarboxylation to succinate	GO:0006540	2	100	9.21E-04
GO:BP	gamma-aminobutyric acid biosynthetic process	GO:0009449	2	40	0.00913393
GO:BP	extracellular matrix organization	GO:0030198	10	2.9069767	5.43E-09
GO:BP	collagen fibril organization	GO:0030199	5	7.5757575	1.24E-05
GO:CC	extracellular matrix	GO:0031012	11	1.7973856	6.88E-08
GO:BP	tube development	GO:0035295	8	0.6932409	0.04383815
GO:BP	gland development	GO:0048732	6	1.2121212	0.01980764
GO:BP	supramolecular fiber organization	GO:0097435	8	0.9367682	0.00513379
REACTOME	GABA synthesis	R-HSA:888568	2	100	9.21E-04
REACTOME	Transcriptional Regulation by MECP2	R-HSA:8986944	3	4.83871	0.03946661
REACTOME	MECP2 regulates transcription of genes involved in GABA signaling	R-HSA:9022927	2	66.666664	0.00275453

Supplementary Table 13D. ClueGO functional enrichment analysis of IFG. This was performed using genes identified from functional clusters.

Source	Term Name	Term ID	Number of Genes	% Associated Genes	Adjusted p-value
GO:MF	endopeptidase activity	GO:0004175	4	0.45045045	0.04962151
GO:BP	neuropeptide hormone activity	GO:0005184	3	7.6923075	8.99E-05
GO:CC	extracellular region	GO:0005576	8	0.17323516	0.00159987
GO:BP	transport	GO:0006810	8	0.16625103	0.00221535
GO:BP	circadian rhythm	GO:0007623	3	1.3452915	0.01640657
GO:BP	regulation of hormone levels	GO:0010817	4	0.6993007	0.00928849
GO:BP	regulation of epinephrine secretion	GO:0014060	2	22.222221	0.00120392
GO:BP	peptide hormone secretion	GO:0030072	3	1.1952192	0.02302402
GO:BP	regulation of localization	GO:0032879	6	0.270636	0.00881848
GO:BP	secretion by cell	GO:0032940	4	0.456621	0.04727692
GO:CC	somatodendritic compartment	GO:0036477	5	0.53590566	0.00235854
GO:CC	synapse	GO:0045202	5	0.34506556	0.0193953

GO:BP	epinephrine secretion	GO:0048242	2	22.222221	0.00120392
GO:BP	positive regulation of transport	GO:0051050	5	0.51229507	0.00292823
GO:BP	negative regulation of glucagon secretion	GO:0070093	2	50	2.03E-04
GO:BP	organic substance transport	GO:0071702	6	0.23148148	0.02125126
GO:BP	nitrogen compound transport	GO:0071705	6	0.28103045	0.0071133
GO:BP	regulation of peptide hormone secretion	GO:0090276	3	1.4925373	0.01214341
REACTOME	Complement factor I binds to MCP, CR1:C4b, C3b	R-HSA:977602	2	28.571428	7.05E-04
REACTOME	Regulation of Complement cascade	R-HSA:977606	2	4.255319	0.03390747
REACTOME	Factor I inactivates MCP/CR1-bound C4b/C3b	R-HSA:977615	2	28.571428	7.05E-04
REACTOME	Complement factor I inactivates C4BP-bound C4b	R-HSA:981637	2	33.333332	5.06E-04
REACTOME	Complement factor I binds C4BP	R-HSA:981658	2	33.333332	5.06E-04
WikiPathways	Circadian rhythm genes	WP:3594	3	1.4492754	0.01320101

Supplementary Table 13E. ClueGO functional enrichment analysis of DLPFC. This was performed using genes identified from functional clusters.

Source	Term Name	Term ID	Number of Genes	% Associated Genes	Adjusted p-value
GO:BP	neuropeptide hormone activity	GO:0005184	2	5.1282053	0.03686154
GO:BP	ion transmembrane transporter activity	GO:0015075	5	0.4382121	0.0473809
GO:BP	steroid hormone secretion	GO:0035929	2	6.6666665	0.02183643
GO:BP	positive regulation of lipid localization	GO:1905954	3	2.5	0.00672486
GO:BP	positive regulation of steroid hormone secretion	GO:2000833	2	16.666666	0.0033938
WikiPathways	Orexin receptor pathway	WP:5094	3	1.9354838	0.0143214

Supplementary Table 13F. ClueGO functional enrichment analysis of CER. This was performed using genes identified from functional clusters.

Source	Term Name	Term ID	Number of Genes	% Associated Genes	Adjusted p-value
GO:CC	nucleosome	GO:0000786	3	2.189781	0.01251685
GO:BP	mature B cell apoptotic process	GO:0002901	2	28.571428	0.0011562
GO:BP	negative regulation of mature B cell apoptotic process	GO:0002906	2	28.571428	0.0011562
GO:MF	dopachrome isomerase activity	GO:0004167	2	28.571428	0.0011562
GO:BP	DNA replication-dependent chromatin assembly	GO:0006335	2	6.25	0.01207662
GO:BP	DNA replication-independent chromatin assembly	GO:0006336	2	6.060606	0.01192748
GO:BP	nitric oxide biosynthetic process	GO:0006809	2	2.4096386	0.01134179
GO:BP	positive regulation of protein kinase A signaling	GO:0010739	2	13.333333	0.00426039
GO:BP	astrocyte development	GO:0014002	2	4.347826	0.01779011
GO:BP	negative regulation of angiogenesis	GO:0016525	3	2.857143	0.00691397
GO:BP	peptidyl-cysteine S-nitrosylation	GO:0018119	2	10	0.00667564
GO:BP	positive regulation of B cell proliferation	GO:0030890	2	4.1666665	0.01742055
GO:CC	platelet alpha granule lumen	GO:0031093	2	2.739726	0.01324853
GO:BP	positive regulation of lipopolysaccharide-mediated signaling pathway	GO:0031666	2	14.285714	0.00385611

GO:BP	sequestering of zinc ion	GO:0032119	2	25	0.00144093
GO:BP	negative regulation of myeloid cell apoptotic process	GO:0033033	2	8.695652	0.00753676
GO:MF	Toll-like receptor 4 binding	GO:0035662	2	40	6.05E-04
GO:BP	intrinsic apoptotic signaling pathway in response to DNA damage by p53 class mediator	GO:0042771	2	3.9215686	0.01745898
GO:BP	negative regulation of cysteine-type endopeptidase activity involved in apoptotic process	GO:0043154	2	2.2727273	0.00635253
GO:BP	positive regulation of cysteine-type endopeptidase activity involved in apoptotic process	GO:0043280	4	2.9411764	0.00104957
GO:BP	regulation of cysteine-type endopeptidase activity involved in apoptotic process	GO:0043281	6	2.6666667	1.98E-05
GO:BP	negative regulation of DNA damage response, signal transduction by p53 class mediator	GO:0043518	2	11.111111	0.00592315
GO:BP	regulation of nitric oxide biosynthetic process	GO:0045428	2	3.0769231	0.01407509
GO:BP	negative regulation of megakaryocyte differentiation	GO:0045653	2	10.526316	0.00631377
GO:BP	positive regulation of angiogenesis	GO:0045766	4	2.173913	0.00276484
GO:BP	positive regulation of fibroblast proliferation	GO:0048146	2	3.3898306	0.01454551
GO:MF	phenylpyruvate tautomerase activity	GO:0050178	2	33.333332	8.80E-04
GO:BP	arachidonic acid secretion	GO:0050482	2	5.4054055	0.01384108
GO:MF	arachidonic acid binding	GO:0050544	2	33.333332	8.80E-04
GO:BP	defense response to fungus	GO:0050832	3	4.83871	0.00217548
GO:BP	positive chemotaxis	GO:0050918	4	5.4054055	1.08E-04
GO:BP	regulation of chemotaxis	GO:0050920	6	2.419355	3.41E-05
GO:BP	negative regulation of chemotaxis	GO:0050922	4	5.5555553	9.98E-05
GO:BP	positive regulation of prostaglandin secretion involved in immune response	GO:0061078	2	50	3.85E-04
GO:BP	positive regulation of myeloid leukocyte cytokine production involved in immune response	GO:0061081	2	5.1282053	0.0140928
GO:BP	neutrophil aggregation	GO:0070488	2	100	6.96E-05
GO:BP	glomerulus vasculature development	GO:0072012	2	6.060606	0.01192748
GO:BP	regulation of monocyte chemotaxis	GO:0090025	3	9.375	3.80E-04
GO:BP	positive regulation of arachidonic acid secretion	GO:0090238	2	20	0.00223217
GO:BP	prostaglandin secretion involved in immune response	GO:0090323	2	50	3.85E-04
GO:BP	cell-cell adhesion mediator activity	GO:0098632	2	3.508772	0.01902687
GO:BP	negative regulation of extrinsic apoptotic signaling pathway via death domain receptors	GO:1902042	2	6.060606	0.01192748
GO:BP	negative regulation of intrinsic apoptotic signaling pathway in response to DNA damage by p53 class mediator	GO:1902166	2	9.523809	0.00700354
GO:BP	macrophage migration	GO:1905517	3	4.347826	0.00277291
GO:BP	regulation of macrophage migration	GO:1905521	3	5.882353	0.00134434
GO:BP	negative regulation of leukocyte apoptotic process	GO:2000107	2	3.4482758	0.01687706
GO:BP	positive regulation of chemokine (C-X-C motif) ligand 2 production	GO:2000343	2	13.333333	0.00426039
GO:BP	negative regulation of cellular senescence	GO:2000773	2	7.6923075	0.00908919
